# Targeting central thalamus with transcranial ultrasound reduces arousal in healthy volunteers

**DOI:** 10.1038/s42003-026-10637-7

**Published:** 2026-10-26

**Authors:** Amber R. Hopkins, Joshua A. Cain, Elizabeth A. White, Stephanie M. Abakians, Martin M. Monti

**Affiliations:** 1 Department of Psychology, University of California Los Angeles, Los Angeles, CA USA; 2 Brain Injury Research Center, Department of Neurosurgery, University of California Los Angeles, Los Angeles, CA USA

**Keywords:** Consciousness, Perception

## Abstract

Using low-intensity focused transcranial ultrasound stimulation, we test the causal roles of the central thalamus in arousal and the pulvinar in visuospatial attention in the healthy human brain. In a within-subjects, counterbalanced design, 27 healthy volunteers (two females) complete a vigilance task (Psychomotor Vigilance Task) and a visuospatial attention task (Egly-Driver Task) before and after sham, central thalamic, and pulvinar sonication. Targeting the central thalamus slows responses and increases lapses in attention during the vigilance task, indicating reduced arousal. Pulvinar sonication reduces (and slows) responses to visual stimuli presented contralaterally, suggesting a disruption of contralateral visuospatial attention. These results provide causal evidence linking the pulvinar to visuospatial attention and the central thalamus to arousal in the healthy human brain and demonstrate that ultrasound can elicit distinct behavioral changes in healthy volunteers when delivered to close but functionally distinct thalamic regions, underscoring its potential as a tool for noninvasive brain mapping.

## Abstract (web summary)

Transcranial ultrasound stimulation causally links the central thalamus to arousal and the pulvinar to visuospatial attention in the healthy human brain.

## Introduction

The thalamus and its interactions with the cortex play a central role in perception, attention, and consciousness. However, because common neurostimulation techniques that are safe to use with healthy volunteers, such as transcranial magnetic stimulation (TMS), cannot directly target subcortical tissue, current understanding about the functional roles of deep brain structures like the thalamus in the healthy human brain relies on either correlational neuroimaging studies with healthy volunteers or lesion and invasive neurostimulation work in nonhuman animal models and human patient populations. The emerging technique of low-intensity focused transcranial ultrasound stimulation (TUS) addresses this gap by allowing safe, noninvasive modulation of subcortical tissue in the healthy human brain^1^. Using TUS, we aim to test the causal roles of two close but functionally distinct regions of the thalamus in the healthy human brain: the pulvinar and the central thalamus.

A growing body of work implicates the central thalamus, which comprises the intralaminar thalamic nuclei and adjacent tissue, in arousal regulation and the level of consciousness. This region not only bridges the brainstem and cortex along the ascending reticular activating system^2^ but also, through its extensive reciprocal connections with the cortex, is well positioned to facilitate cortical synchrony and electrocortical arousal^3^. Indeed, invasive electrical stimulation of the central thalamus in awake nonhuman primates can increase signs of arousal^4^ or induce lapses in consciousness^5^, depending on the stimulation parameters. Strikingly, behavioral and electrocortical markers of arousal have been restored by targeting the central thalamus with pharmacological activation in anesthetized rodents^6–8^ and invasive electrical stimulation in anesthetized nonhuman primates^9–12^. Moreover, invasive deep brain stimulation and, more recently, TUS to the central thalamus in patients with disorders of consciousness (DOC) can yield improvements in their level of consciousness^13^.

The pulvinar nucleus, which subsumes the posterior third of the thalamus, shows activation and interactions with the cortex that are consistent with a role in visual processing and attention. The pulvinar is reciprocally connected with both visual cortices and higher-order frontal and parietal areas^14^. Visual responses modulated by attention can be recorded from the pulvinar^15^ and greater pulvinar activation is observed under states of engaged visual attention^16^. Beyond showing attention-dependent activation, the pulvinar also appears to play an active, coordinating role in visuospatial attention. Recent work reports that the pulvinar synchronizes cortical activity, regulating the transmission of information between cortical areas according to attentional demands^17^. Another study demonstrated that activity propagates from the pulvinar to the cortex during states of engaged visual attention but from the cortex to the pulvinar during disengaged states^18^. Moreover, lesions and deactivation of the pulvinar have been associated with deficits in visuospatial attention in nonhuman primates^19,20^ and patients^21,22^, especially for the contralateral visual field and when stimuli appear in multiple locations and compete for attention. Indeed, unilateral deactivation of the pulvinar in behaving nonhuman primates caused diminished exploration of the contralesional visual field, particularly when stimuli appeared in both visual fields^19^.

**Fig. 1:**
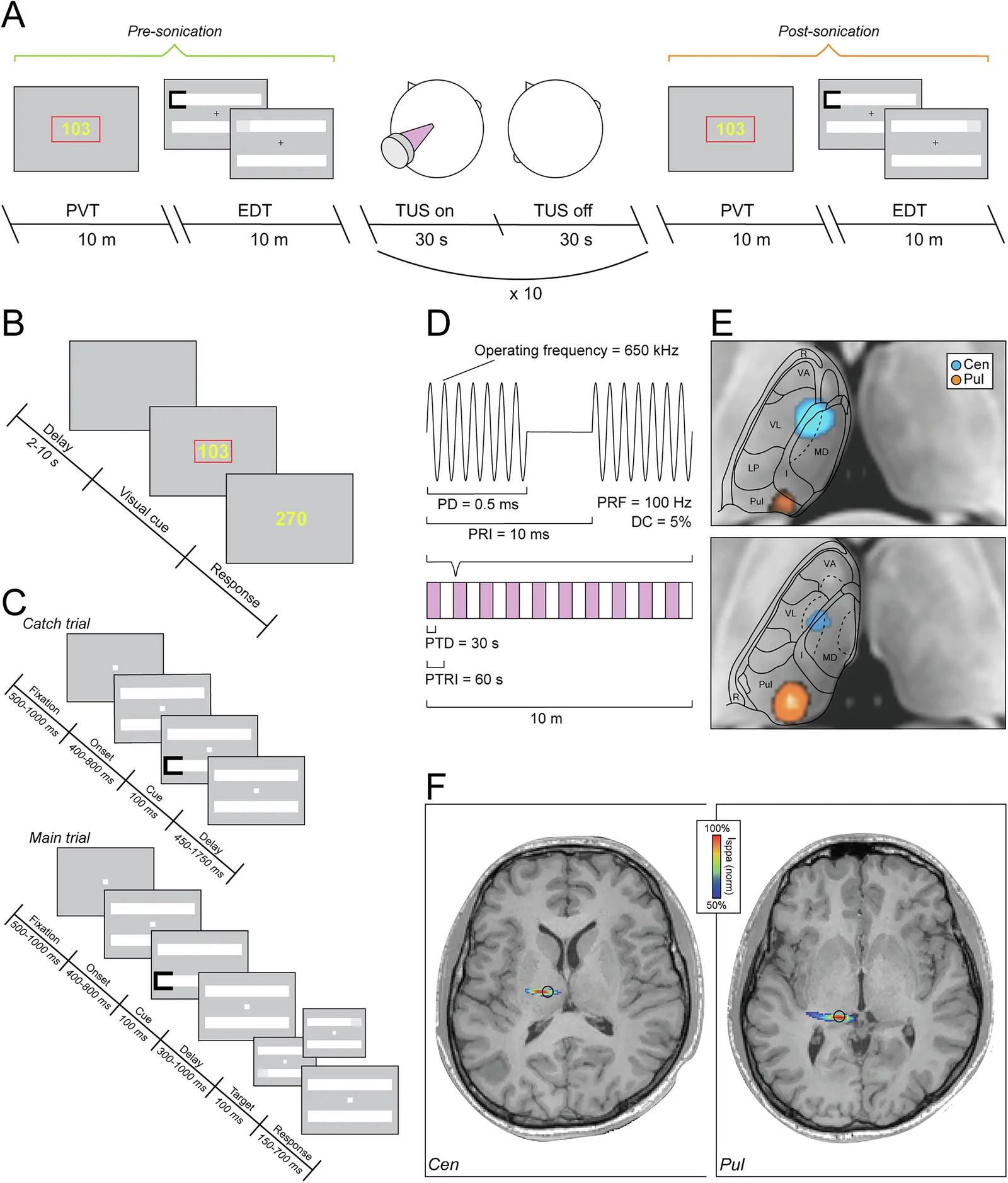
Methods. **A** Session schematic. Participants completed the PVT and EDT before and after sham, pulvinar, or central thalamic sonication with low-intensity and focused TUS in separate sessions on different days. Session and task order were counterbalanced across participants. **B** PVT. Participants maintained central fixation and pressed a button when a visual cue (yellow counter) appeared for 10 min without breaks. **C** EDT. Participants maintained central fixation and pressed a button if they detected a visual target. On main trials, a spatial cue indicated where the target was most likely to appear (with 75% cue validity). On catch trials, no target followed the cue. **D** Sonication parameters. 10 blocks, each with 30 s of rest (white) and 30 s of sonication (purple) with the following parameters: operating frequency = 650 kHz, PD = 0.5 ms, PRI = 10 ms, DC = 5% (PD/PRI), PRF = 100 Hz (1/PRI), I_spta.3_ ≈ 720 mW/cm^2^, and I_sppa.3_ ≈ 14.40 W/cm^2^. **E** Target regions. Heatmap of TUS target regions for the left central thalamus (blue) and left pulvinar (orange) among participants in standard space, ranging from 98 to 100%. **F** Simulated acoustic effects achieved when targeting the left central thalamus (left) and left pulvinar (right) with TUS in an example participant. The ultrasound focus is colored by the amount of acoustic pressure achieved, ranging from 50% of the maximum (blue) to the maximum (red). The left central thalamus and left pulvinar target regions defined for the participant are outlined in black. Abbreviations: PVT psychomotor vigilance task, EDT Egly-Driver task, TUS transcranial ultrasound stimulation, PD pulse duration, PRI pulse repetition interval, PTD pulse train duration, PTRI pulse train repetition interval, DC duty cycle, PRF pulse repetition frequency, Cen central thalamus, Pul pulvinar.

Leveraging the capability of TUS to noninvasively modulate subcortical structures in healthy volunteers, we test the causal roles of the central thalamus in arousal and the pulvinar in visuospatial attention in the healthy human brain. In a within-subjects, sham-controlled, and counterbalanced design, 27 healthy volunteers completed the Psychomotor Vigilance Task (PVT)^23^ and Egly-Driver Task (EDT)^24^ before and after central thalamic, pulvinar, and sham sonication with TUS at parameters considered suppressive^25–28^ (see Fig. 1). The PVT assesses the ability to maintain vigilance over uninterrupted periods. States of diminished arousal like sleep deprivation are reliably accompanied by reduced behavioral markers of vigilance during the PVT, including slower responses and more lapses in attention^29,30^. The time-on-task effect (also called the vigilance decrement) for the PVT, whereby responses slow and lapses increase over the duration of the task, is also exaggerated under reduced arousal. In contrast, the EDT is a visuospatial attention task that assesses the ability to detect validly or invalidly cued visual targets in varying spatial locations^24^. Patients with unilateral parietal lesions show a greater cost of invalid cuing for contralesional targets in the EDT^24^, and there is evidence that dorsal aspects of the pulvinar have a similar role in attention as posterior parietal cortex^20,31^. A recent study with nonhuman primates completing the EDT reported that the pulvinar was key for maintaining attention at cued locations^18^.

Given the putative roles of the central thalamus in arousal and the pulvinar in visuospatial attention, we expect their suppression with TUS to cause distinct effects on cognition that would be reflected differently in the PVT and EDT. While targeting the central thalamus with suppressive TUS should impair performance on both tasks in a manner consistent with a broad reduction in alertness or arousal, we expect the effects of central thalamic sonication to be most evident during the PVT. Specifically, we expect slower responses and more frequent lapses in attention after targeting the central thalamus compared to sham and pulvinar sonication. In contrast, we expect pulvinar sonication with suppressive TUS to affect visuospatial attention during the EDT, especially for the contralateral visual field. This would be evidenced by reduced detection and slower responses to visual stimuli presented contralaterally to the sonicated region, compared to the sham and central thalamus conditions.

In this work, we demonstrate that targeting the central thalamus with TUS elicits changes in behavior consistent with a reduction in arousal, whereas pulvinar sonication leads to a selective disruption of visuospatial attention for the contralateral visual field. Compared to sham and pulvinar sonication, central thalamic sonication was associated with slower responses and more frequent lapses in attention during the PVT as well as reduced visual target detection during the EDT, whether the targets appeared ipsilaterally or contralaterally to the TUS region. In contrast, compared to sham and central thalamic sonication, targeting the pulvinar with TUS was associated with reduced responsiveness to contralateral spatial cues, reduced detection of validly cued contralateral targets, and slowed responses to invalidly cued contralateral targets. These results provide causal evidence in healthy volunteers for the functional roles of the central thalamus and pulvinar, highlighting the potential of TUS as a high-resolution tool for causal brain mapping of deep brain regions in the healthy human brain.

## Results

### Psychomotor vigilance task (PVT)

**Fig. 2:**
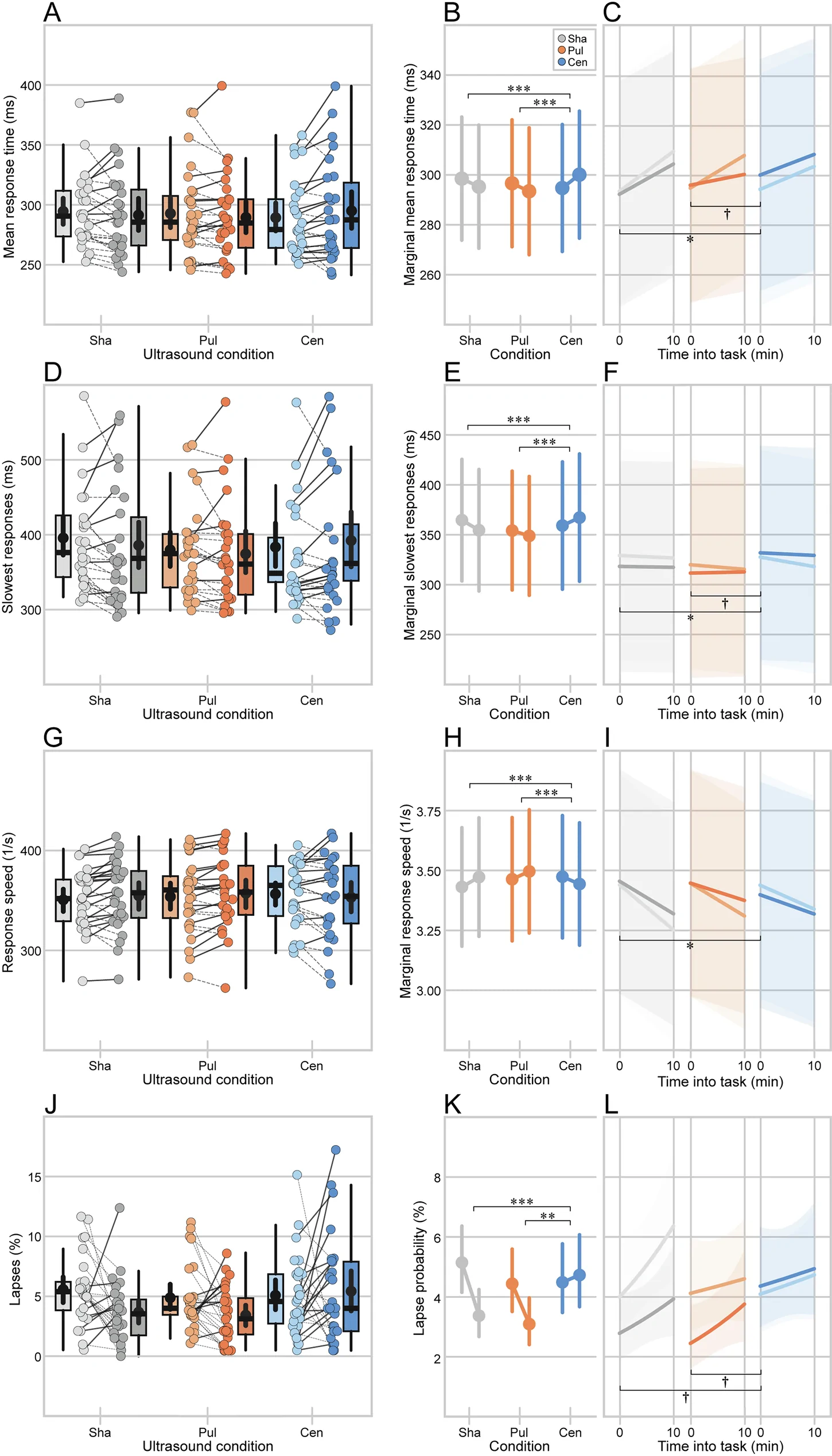
Central thalamic sonication reduced vigilance during the psychomotor vigilance task (PVT). **A**,** D**,** G**,** J** Distribution of response times (**A**; *n* = 26), slowest 10% of responses (**D**; *n* = 24), response speeds (**G**; *n* = 26), and lapses in attention (**J**; *n* = 27) before and after sonication (left to right, light to dark) for each ultrasound condition from the data used in the models. Boxes show first quartiles, medians, and third quartiles. Whiskers span the data points within 1.5 × the IQR from the lower and upper hinges. Black points and bars show means and 95% confidence intervals across participants, respectively. Individual points are participant means. Lines connect participant means across blocks, tracking their change in the outcome from before to after sonication (left to right). Lines are dashed or solid if the participant showed a higher value of the outcome before or after sonication, respectively. Estimated marginal means for response times (**B**), the slowest 10% of responses (**E**), response speeds (**H**), and the predicted probability of showing a lapse in attention (**K**) before and after sonication (from left to right) for each ultrasound condition. Bars show 95% confidence intervals. Predicted changes in response times (**C**), the slowest 10% of responses (**F**), response speeds (**I**), and the probability of showing a lapse in attention (**L**) over the duration of the task block (in minutes) before (light) and after sonication (dark) for each ultrasound condition. Bands show 95% confidence intervals. Abbreviations: Sha sham, Pul pulvinar, Cen central thalamus.$\,{\dagger}$ *p* < 0.10 before and/or after correction, * *p* < 0.05, ** *p* < 0.01, *** *p* < 0.001 after correction.

Mixed-effects models testing the three-way interaction between ultrasound condition (sham, pulvinar, and central thalamic sonication), block (before and after sonication), and time into the task block (time-on-task effect in minutes) and estimated marginal means contrasts were used to examine whether central thalamic and pulvinar sonication affected behavioral markers of vigilance during the PVT^23^, including response times, the slowest 10% of responses, response speeds, and lapses in attention (defined as unusually slow responses). These outcomes were selected because of their sensitivity to states of diminished arousal like sleep deprivation^29,30^. Data cleaning results are provided in Supplementary Fig. 1 and Supplementary Table 1. Descriptive statistics for the raw data used in the mixed-effects models are presented in Fig. 2A, D, G, J, as well as in Supplementary Fig. 2. Additional model results are presented in Supplementary Figs. 3–7 and Supplementary Tables 2–13. Numerical source data for Fig. 2 is provided in Supplementary Data 1.

Targeting the central thalamus with TUS slowed responses and increased lapses in attention during the PVT compared to sham and pulvinar conditions, on average. Response times increased by 8.56 ms compared to sham (SE = 1.40, 95% CI = [5.82, 11.30], *z* = 6.13, *p*< 0.001, *p*_adj_ < 0.001) and by 8.55 ms compared to pulvinar (SE = 1.40, 95% CI = [5.80, 11.29], *z* = 6.11, *p* < 0.001, *p*_adj_ < 0.001) sonication (see Fig. 2B). The slowest 10% of responses increased by 18.07 ms compared to sham (SE = 3.37, 95% CI = [11.47, 24.67], *z* = 5.36, *p* < 0.001, *p*_adj_ < 0.001) and by 13.28 ms compared to pulvinar (SE = 3.40, 95% CI = [6.61, 19.95], *z* = 3.90, *p* < 0.001, *p*_adj_ < 0.001) sonication (see Fig. 2E). Consistent with these results, response speeds decreased following central thalamic sonication by 0.07 1/s compared to sham (SE = 0.01, 95% CI = [−0.10, −0.04], *z* = −5.12, *p* < 0.001, *p*_adj_ < 0.001) and by 0.06 1/s compared to pulvinar (SE = 0.01, 95% CI = [−0.09, −0.04], *z* = −4.51, *p* < 0.001, *p*_adj_ < 0.001) sonication (see Fig. 2H). Central thalamic sonication also increased the likelihood of lapses in attention compared to the sham (odds ratio = 1.64, 95% CI = [1.28, 2.12], *z* = 3.84, *p* < 0.001, *p*_adj_ < 0.001) and pulvinar (odds ratio = 1.54, 95% CI = [1.19, 1.99], *z* = 3.25, *p* = 0.001, *p*_adj_ = 0.003) conditions (see Fig. 2K). There were no differences in how the outcomes changed after sonication between the sham and pulvinar conditions.

The canonical time-on-task effects for the PVT were replicated, with response times slowing by 1.29 ms per minute (SE = 0.14, 95% CI = [1.02, 1.57], *z* = 9.27, *p* < 0.001, *p*_adj_ < 0.001; see Fig. 2C), response speeds decreasing by 0.01 1/s per minute (SE = 0.00, 95% CI = [−0.02, −0.01], *z* = −10.12, *p* < 0.001, *p*_adj_ < 0.001; see Fig. 2I), and the likelihood of lapses in attention increasing over the duration of each block (odds ratio = 1.03, 95% CI = [1.00, 1.05], *z* = 2.03, *p* = 0.042, *p*_adj_ = 0.063; see Fig. 2L). However, no sonication condition altered these effects. Rather, slower responses and more frequent lapses in attention were evident promptly at task onset following central thalamic sonication (0 min into the post-sonication task block) and persisted for the duration of the task. Specifically, response times at task onset following central thalamic sonication were greater by 7.09 ms compared to sham (SE = 2.76, 95% CI = [1.68, 12.51], *t* = 2.57, *p* = 0.010) and by 4.69 ms compared to pulvinar (SE = 2.77, 95% CI = [−0.73, 10.11], *z* = 1.70, *p* = 0.090, *p*_adj_ = 0.13) sonication (see Fig. 2C). Similarly, the slowest 10% of response times were greater by 15.28 ms compared to sham (SE = 6.97, 95% CI = [1.61, 28.96], *t* = 2.19, *p* = 0.029) and by 12.64 ms compared to pulvinar (SE = 7.11, 95% CI = [−1.29, 26.58], *z* = 1.78, *p* = 0.075, *p*_adj_ = 0.15) sonication (see Fig. 2F). Conversely, response speeds at task onset following central thalamic sonication were lower by 0.06 1/s compared to sham (SE = 0.03, 95% CI = [−0.11, 0.00], *t* = -2.01, *p* = 0.045; see Fig. 2I). Finally, the odds of lapsing at task onset after central thalamic sonication were 1.55 times greater than sham (95% CI = [0.93, 2.60], *z* = 1.68, *p* = 0.094) and 1.82 times greater than pulvinar (95% CI = [1.08, 3.09], *z* = 2.24, *p* = 0.025, *p*_adj_ = 0.050) sonication (see Fig. 2l).

### Egly-Driver task (EDT)

**Fig. 3:**
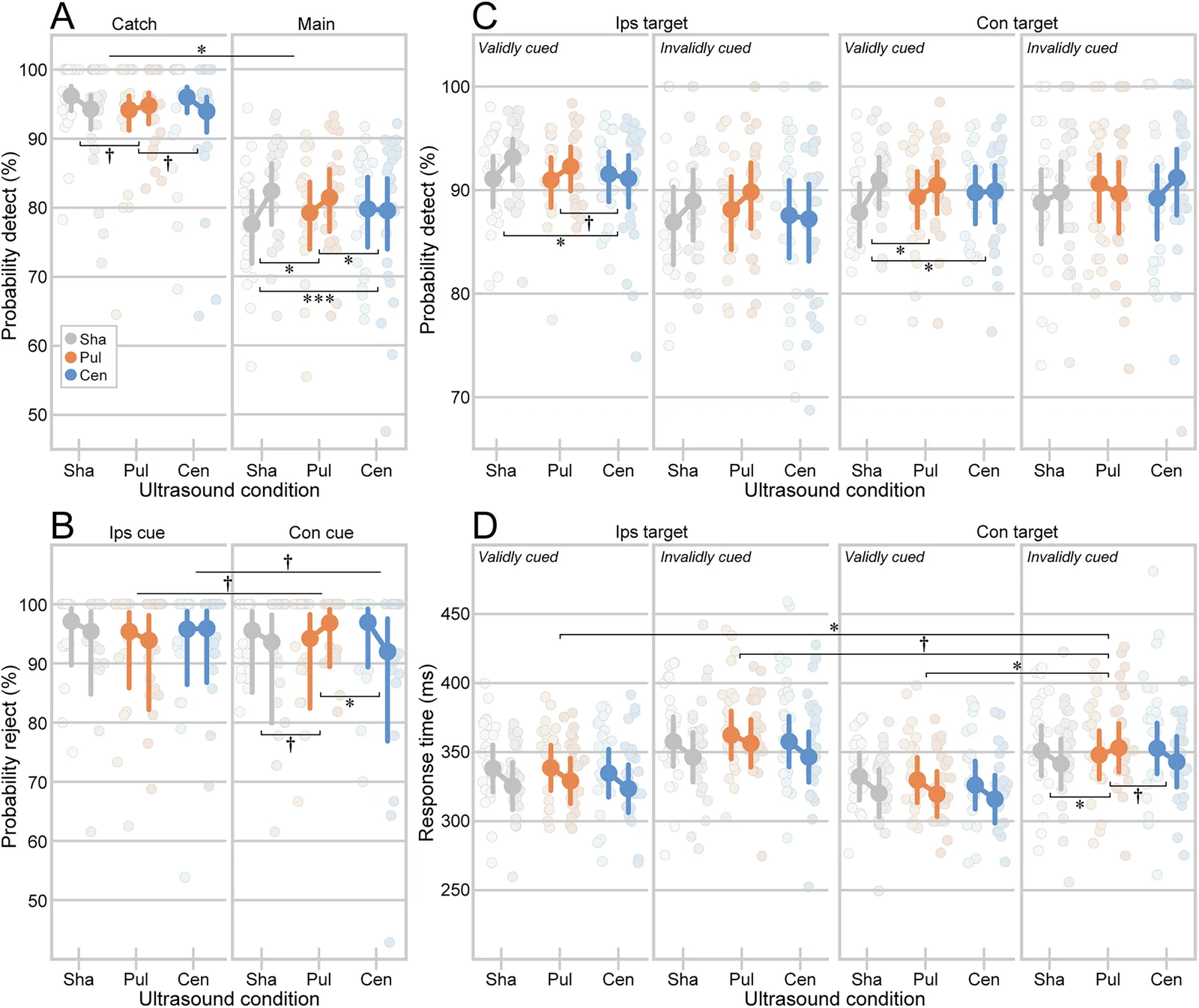
Targeting the central thalamus and pulvinar with TUS had different effects on visuospatial performance during the Egly-Driver Task (EDT). Bars show 95% confidence intervals. Transparent points show participant means from the raw data used in the mixed-effects models. **A** Estimated marginal means for the predicted probability of responding correctly on catch trials (left) and main trials (right) before and after sonication (left to right) for each ultrasound condition, on average (*n* = 26). **B** Estimated marginal means for the predicted probability of responding correctly (“correct rejections”) on catch trials with ipsilateral (left) and contralateral (right) spatial cues for each condition before and after sonication (left to right; *n* = 27). **C**, **D** Estimated marginal means for the predicted probability of responding correctly (**C**; *n* = 26) and estimated marginal response times (**D**; *n* = 27) on main trials with validly cued ipsilateral targets (first panel), invalidly cued ipsilateral targets (second panel), validly cued contralateral targets (third panel), and invalidly cued contralateral targets (fourth panel). Abbreviations: Sha sham, Pul pulvinar, Cen central thalamus, Ips ipsilateral, Con contralateral. † p < 0.10 before and/or after correction, * *p* < 0.05, ** *p* < 0.01, *** *p* < 0.001 after correction.

Mixed-effects models and estimated marginal means contrasts were used to examine whether pulvinar or central thalamic sonication affected correct responses and response times during the EDT^24^. Separate mixed-effects logistic regressions were used to assess whether the effect of sonication condition (sham, central thalamus, pulvinar) on correct responses depended on trial type (catch or main), spatial cue location (considering catch trials only), or spatial cue validity and visual target hemifield (considering main trials only). A linear mixed-effects model was used to examine whether the effects of sonication on response times during main trials depended on spatial cue validity and visual target hemifield. Data cleaning results are provided in Supplementary Fig. 9. Descriptive statistics for the underlying raw data used in the mixed-effects models are presented in Fig. 3 as well as in Supplementary Fig. 10. Additional model results are presented in Supplementary Figs. 11–14 and Supplementary Tables 14–25. Numerical source data for Fig. 3 is provided in Supplementary Data 2.

#### Catch trials

On average, participants responded to spatial cues less often after pulvinar sonication, as indicated by a greater proportion of “correct rejections” arising from fewer responses compared to the sham (odds ratio = 1.72, 95% CI = [1.05, 3.23], *z* = 1.68, *p* = 0.092; see Fig. 3A, left) and central thalamus (odds ratio = 1.72, 95% CI = [0.90, 3.33], *z* = 1.65, *p* = 0.099, *p*_adj_ = 0.14; see Fig. 3A, left) conditions. While these comparisons were marginally significant before correction, the direction and range of the confidence intervals indicate a trend. After separating catch trials by spatial cue location, it is evident that the effect of pulvinar sonication on catch trial responses was driven by a selective reduction in responses to contralateral spatial cues. Specifically, participants were more likely to reject contralateral spatial cues on catch trials after pulvinar sonication compared to sham (odds ratio = 2.77, 95% CI = [0.97, 7.93], *z* = 1.90, *p* = 0.058; see Fig. 3B, right) and central thalamic sonication (odds ratio = 5.18, 95% CI = [1.72, 15.65], *z* = 2.92, *p* = 0.004, *p*_adj_ = 0.028; see Fig. 3B, right). In contrast, participants were no more or less likely to reject ipsilateral spatial cues after pulvinar sonication compared to sham (odds ratio = 1.20, 95% CI = [0.44, 3.27], *z* = 0.35, *p* = 0.72, *p*_adj_ = 0.72; see Fig. 3B, left) or central thalamic sonication (odds ratio = 0.72, 95% CI = [0.27, 1.89], *z* = −0.67, *p* = 0.51, *p*_adj_ = 0.59; see Fig. 3B, left).

#### Main trials

The typical behavioral costs of disengaging spatial attention from a cued location to respond to a visual target presented in an uncued location during the EDT were replicated. Specifically, participants were more likely to detect validly cued visual targets than invalidly cued visual targets at baseline (before sonication), on average (odds ratio = 1.21, 95% CI = [1.08, 1.35], *z* = 3.24, *p* = 0.001, *p*_adj_ = 0.018; see Fig. 3C). In addition, participants took 21.57 ms (SE = 1.41, 95% CI = [18.80, 24.34], *z* = 15.27, *p* < 0.001, *p*_adj_ < 0.001) longer to respond to invalidly cued visual targets than validly cued visual targets at baseline (before sonication), on average (see Fig. 3D).

Central thalamic sonication broadly impaired visual target detection during the EDT. On average, participants were less likely to detect the visual target after central thalamic sonication compared to sham (odds ratio = 0.73, 95% CI = [0.64, 0.83], *z* = −4.62, *p* < 0.001, *p*_adj_ < 0.001; see Fig. 3A, right) and pulvinar sonication (odds ratio = 0.86, 95% CI = [0.75, 0.98], *z* = −2.31, *p* = 0.021, *p*_adj_ = 0.035; see Fig. 3A, right). After separating main trials according to spatial cue validity and visual target hemifield, the effect of central thalamic sonication on visual target detection was observed in all validly cued visual targets, whether ipsilateral or contralateral to the sonicated region. Specifically, participants were less likely to detect validly cued ipsilateral visual targets after central thalamic sonication compared to sham (odds ratio = 0.69, 95% CI = [0.55, 0.86], *z* = −3.24, *p* = 0.001, *p*_adj_ = 0.018; see Fig. 3C, first panel) and pulvinar sonication (odds ratio = 0.78, 95% CI = [0.63, 0.97], *z* = −2.21, *p* = 0.027, *p*_adj_ = 0.32; see Fig. 3C, first panel). Similarly, participants were less likely to detect validly cued contralateral visual targets after central thalamic sonication compared to sham sonication (odds ratio = 0.70, 95% CI = [0.57, 0.86], *z* = −3.34, *p* = 0.001; see Fig. 3C, third panel). Central thalamic sonication was not associated with any changes in response time during the EDT when compared to the sham condition (see Fig. 3D).

Pulvinar sonication was also associated with reduced visual target detection, as participants detected visual targets less often after pulvinar sonication compared to sham sonication, on average (odds ratio = 0.85, 95% CI = [0.74, 0.97], *z* = −2.33, *p* = 0.020, *p*_adj_ = 0.035; see Fig. 3A, right). However, after separating main trials according to spatial cue validity and visual target location, the effect of pulvinar sonication on visual target detection appeared to be driven by a selective reduction in the detection of contralateral targets. Specifically, participants were less likely to detect validly cued contralateral visual targets on main trials compared to sham sonication (odds ratio = 0.79, 95% CI = [0.64, 0.98], *z* = −2.15, *p* = 0.031; see Fig. 3C, third panel). In contrast, participants were no more or less likely to detect validly cued ipsilateral targets after pulvinar sonication, compared to sham sonication (odds ratio = 0.88, 95% CI = [0.70, 1.11], *z* = −1.09, *p* = 0.27, *p*_adj_ = 0.63; see Fig. 3C, first panel).

The disruption of contralateral target detection after pulvinar sonication is also consistent with the response time data. Specifically, after pulvinar sonication, participants took longer to respond to invalidly cued contralateral visual targets compared to sham (estimate = 14.69 ms, SE = 6.74, 95% CI = [1.49, 27.90], *z* = 2.18, *p* = 0.029; see Fig. 3D, fourth panel) and central thalamic sonication (estimate = 14.75 ms, SE = 6.73, 95% CI = [1.56, 27.94], *z* = 2.19, *p* = 0.028, *p*_adj_ = 0.25; see Fig. 3D, fourth panel). While the comparison to the central thalamus condition did not pass correction, the direction and range of the confidence intervals indicate a trend. In contrast, there were no differences in response times to invalidly cued ipsilateral targets after sonication between the pulvinar and sham conditions (estimate = 5.34 ms, SE = 6.05, 95% CI = [−6.52, 17.21], *z *= 0.88, *p* = 0.38, *p*_adj_ = 0.97; see Fig. 3D, second panel) or the central thalamus and pulvinar conditions (estimate = −5.17 ms, SE = 6.12, 95% CI = [−17.17, 6.83], *z* = −0.84, *p* = 0.40, *p*_adj_ = 0.97; see Fig. 3D, second panel). Indeed, following pulvinar sonication, responses to invalidly cued contralateral targets were 11.17 ms slower than for invalidly cued ipsilateral targets (SE = 6.47, 95% CI = [−1.50, 23.85], *z* = 1.73, *p* = 0.084, *p*_adj_ = 0.42; see Fig. 3D, second and fourth panels). While this did not reach significance when compared to sham (estimate = 9.35 ms, SE = 9.05, 95% CI = [−8.39, 27.10], *z* = 1.03, *p* = 0.30) or central thalamic sonication (estimate = 9.58 ms, SE = 9.10, 95% CI = [−8.25, 27.41], *z* = 1.05, *p* = 0.29, *p*_adj_ = 0.97), the confidence intervals indicate a trend.

The slowing of responses after pulvinar sonication was limited to invalidly cued contralateral targets. There were no differences in response times to validly cued ipsilateral targets after pulvinar sonication compared to sham sonication (estimate = 3.26 ms, SE = 2.44, 95% CI = [−1.52, 8.04], *z* = 1.34, *p* = 0.18, *p*_adj_ = 0.80; see Fig. 3D, first panel) and central thalamic sonication (estimate = −1.71 ms, SE = 2.42, 95% CI = [−6.45, 3.03], *z* = −0.71, *p* = 0.48, *p*_adj_ = 0.97; see Fig. 3D, first panel), or to validly cued contralateral targets after pulvinar sonication compared to sham sonication (estimate = 2.01 ms, SE = 2.52, 95% CI = [−2.93, 6.95], *z* = 0.80, *p* = 0.43, *p*_adj_ = 0.97; see Fig. 3D, third panel) and central thalamic sonication (estimate = −0.03 ms, SE = 2.48, 95% CI = [−4.90, 4.84], *z* = −0.01, *p* = 0.99, *p*_adj_ = 0.99; see Fig. 3D, third panel). Indeed, responses to invalidly cued contralateral targets were 14.67 ms slower (SE = 5.14, 95% CI = [4.60, 24.73], *z* = 2.86, *p* = 0.004, *p*_adj_ = 0.047) than for validly cued ipsilateral targets after pulvinar sonication, which was 11.43 ms (SE = 7.16, 95% CI = [−2.60, 25.47], *z* = 1.60, *p* = 0.11) and 13.04 ms (SE = 7.15, 95% CI = [−0.97, 27.06], *z* = 1.82, *p* = 0.068, *p*_adj_ = 0.40) greater than the changes in response times across blocks in the sham and central thalamus conditions, respectively (see Fig. 3D, third and fourth panels). Similarly, responses to invalidly cued contralateral targets were 15.26 ms slower (SE = 5.15, 95% CI = [5.17, 25.36], *z* = 2.96, *p* = 0.003, *p*_adj_ = 0.047) than for validly cued contralateral targets after pulvinar sonication, which was 12.69 ms (SE = 7.18, 95% CI = [−1.40, 26.77], *z* = 1.77, *p* = 0.077) and 14.72 ms (SE = 7.17, 95% CI = [0.67, 28.78], *z* = 2.05, *p* = 0.040, *p*_adj_ = 0.28) greater than the changes in response times across blocks in the sham and central thalamus conditions, respectively (see Fig. 3D, third and fourth panels). While some comparisons were marginally significant or did not pass correction, the directions and ranges of the confidence intervals indicate a trend.

## Discussion

In this work, we demonstrate that targeting the central thalamus with TUS slowed responses and increased lapses in attention during the PVT but also reduced visual target detection during the EDT. These behavioral changes are consistent with a broad reduction in arousal and a suppression of the central thalamus. In contrast, pulvinar sonication led to more specific changes in visuospatial behavior during the EDT. Specifically, participants were less responsive to contralateral spatial cues, detected validly cued contralateral visual targets less often, and took longer to respond to invalidly cued contralateral visual targets after pulvinar sonication compared to the other conditions. These behavioral changes are consistent with a disruption of visuospatial attention for the contralateral visual field specifically and a (unilateral) suppression of the pulvinar nucleus. Taken together, our results provide causal evidence in the healthy human brain linking the central thalamus to arousal and the pulvinar to visuospatial attention. In addition, this work indicates that single-element TUS at the parameters we apply is likely suppressive^25–27^, is sufficient to alter behavior in healthy volunteers over a period of at least 20 minutes, and is spatially precise enough to elicit distinct behavioral changes when applied to different parts of the thalamus in the same individuals.

Reduced vigilance under states of diminished arousal, like sleep deprivation, is reliably accompanied by slower responses and more frequent lapses in attention during the PVT^29,30^. In this work, we observed similar effects after targeting the central thalamus with TUS. Participants took longer to respond and showed lapses in attention more often after central thalamic sonication compared to sham and pulvinar sonication (see Fig. 2). This behavioral profile is consistent with reduced central thalamic engagement. Indeed, under sleep deprivation, lapses in attention are associated with reduced thalamic activation, while non-lapse periods are accompanied by elevated thalamic activation^32^. Thalamic activity in sleep-deprived individuals performing vigilance tasks reflects a complex interplay between the effects of sleep loss, which dampens arousal, and the effects of engaging in a task, which recruits arousal^33^. At rest, thalamic activity is reduced under sleep deprivation compared to rested wakefulness^34,35^. However, during vigilance tasks, the thalamus shows greater activation under sleep deprivation than it does during rested wakefulness. This suggests that the thalamus compensates for the dampening effects of sleep loss to support engagement in the task^33,35^. Some previous work reports that these effects are stronger in more central parts of the thalamus^33,34^. If, as we report here, targeting the central thalamus with TUS emulates the effects of sleep deprivation, then we might expect thalamic activity to be more pronounced during the PVT after central thalamic sonication. Future work could implement our experimental design in the magnetic resonance environment to test whether administering TUS at these parameters to the central thalamus alters the interplay of thalamic activity described during vigilance tasks under sleep deprivation.

Where the effects of central thalamic sonication on PVT performance differ from what is typically reported in the sleep deprivation work involves the time-on-task effect (also called the vigilance decrement), which is characterized by worsening performance over the duration of the task due to factors like fatigue or boredom^36^. This effect, which can be observed during rested wakefulness and is exaggerated under states of diminished arousal like sleep deprivation, presents as a slowing of responses, an increase in attentional lapses, and an increase in response time variability as time-on-task increases in the PVT^29,30^. While our participants exhibit time-on-task effects for the PVT (see Fig. 2), they were not altered by sonication of the central thalamus (or in any other condition). Interestingly, there is some evidence that maintaining vigilance over uninterrupted periods recruits a predominantly right-lateralized network of cortical and subcortical structures, including the right thalamus^37–39^. So, it is possible that targeting the right central thalamus with TUS could modulate within-block time-on-task effects.

Lesions and deactivation of the pulvinar have been associated with deficits in visuospatial attention in humans^21,22^ and nonhuman primates^19,20^, especially for the contralateral visual field. For instance, pulvinar inactivation in behaving nonhuman primates diminishes exploration of the contralesional visual field, particularly when stimuli are presented in both visual fields, thus competing for attention^19^. Behavioral impairments in patients with pulvinar damage are also especially strong when distractor stimuli compete for attention^21,22^. We observe similar deficits in visuospatial attention after targeting the pulvinar with TUS in this work. Participants responded to contralateral spatial cues on catch trials less often after pulvinar sonication compared to the other conditions, reflected by an increase in “correct rejections” (see Fig. 3A, B). In addition, participants detected validly cued contralateral visual targets less often after pulvinar sonication compared to the sham condition (see Fig. 3C). Taken together, the behavioral changes we observe are consistent with a suppression of the pulvinar.

Patients with unilateral parietal lesions show deficits in disengaging spatial attention to respond to contralesional stimuli^40^. More specifically, these patients show slower responses to contralesional visual targets when a spatial cue directed their attention ipsilesionally compared to when the ipsilesional field was cued and the target appeared contralesionally, demonstrating a greater behavioral cost for invalid cuing for contralesional visual targets. Since these patients show similar response times for validly cued ipsilesional and contralesional targets, the greater cost for invalid cuing for the contralesional field has been attributed to impaired ability to disengage spatial attention specifically. Similar effects can be demonstrated with the EDT. For instance, the original EDT paper includes an experiment in which the authors administered the task to patients with unilateral parietal lesions, and they report a greater cost of invalid cuing for contralesional targets during the EDT, replicating the impaired disengagement of spatial attention in these patients^24^.

We observed similar effects by targeting the pulvinar with TUS. There were no differences in response times to validly cued contralateral or ipsilateral targets during the EDT after pulvinar sonication (see Fig. 3D, first and third panels). However, responses to invalidly cued contralateral targets were slower after pulvinar sonication compared to the sham and central thalamus conditions (see Fig. 3D, fourth panel), indicating that pulvinar sonication led to similar deficits in disengaging spatial attention reported previously for patients with unilateral pulvinar lesions. This raises interesting questions about the roles of the pulvinar and parietal cortex in visuospatial attention. For instance, perhaps the original finding linking unilateral parietal lesions to the spatial disengagement deficit in patients could be explained by the pulvinar being unable to act normally on the damaged parietal cortex to coordinate visuospatial attention. Interestingly, there is some evidence that the dorsal portion of the pulvinar has a role in attention like that of the posterior parietal cortex^20,31^. For instance, deactivation of part of the pulvinar impairs attention like parietal deactivation does^15,41^. Future work could apply TUS to the pulvinar and parietal areas implicated in visuospatial attention to more directly compare their contributions.

While the behavioral changes that we observe after pulvinar sonication are consistent with previous lesion and stimulation studies, many of the statistical comparisons were weak and either did not reach significance or did not pass correction. We suggest that this is due to the low number of catch trials and invalidly cued target main trials in combination with the large quantity of comparisons we ran to comprehensively examine and compare the effects of sonication between the different ultrasound conditions. Participants completed 216.07 main trials (SD = 3.93) and 23.93 catch trials (SD = 3.93) per EDT block, on average. On catch trials, the spatial cue appeared ipsilaterally in 13.07 (SD = 2.91) trials and contralaterally in 10.86 (SD = 2.52) trials. On main trials, there were 88.20 validly cued ipsilateral visual targets (SD = 6.49) and 87.33 validly cued contralateral targets (SD = 6.74), but only 15.28 invalidly cued ipsilateral targets (SD = 3.06) and 12.13 invalidly cued contralateral targets (SD = 2.78). Despite the low number of catch trials and invalidly cued main trials, the comparisons we report still indicate trends, considering the direction and range of the confidence intervals. However, additional work administering longer versions of the EDT is necessary to make stronger conclusions about the effects of pulvinar sonication on visuospatial attention using the EDT.

By selecting cue-to-target intervals from a continuous range between 300 and 1100 ms during the EDT, fluctuations in spatial and object attention as a function of the interval between a spatial cue and visual target have been mapped to demonstrate that visuospatial attention is discontinuous, sampling the environment in theta-rhythmic cycles (3–8 Hz)^18,42^. These cycles are thought to organize neural activity into alternating attentional states of engagement (heightened sensitivity to stimuli in the attended location) and disengagement (decreased sensitivity to stimuli in the attended location and increased probability of shifting spatial attention)^42^. While these theta-rhythmic cycles of attention have been linked to theta oscillations in frontal and parietal areas^42^, the pulvinar seems to play a key role^18^. A recent study examined pulvino-cortical interactions during theta-rhythmic sampling of attention by recording the frontal eye fields, lateral intraparietal area, and pulvinar in awake nonhuman primates completing the EDT^18^. They report that activity propagated from pulvinar to cortex during states of engaged attention, but from cortex to the pulvinar during disengaged states. We might thus expect targeting the pulvinar with TUS to affect the theta-rhythmic sampling of attention during the EDT. However, since we used an abbreviated version of the EDT with only short (300 ms) and long (1100 ms) cue-to-target intervals, we cannot explore this with the present data. Future work could administer the full version of the EDT to assess whether suppressing the pulvinar alters theta-rhythmic cycles of attention.

Several limitations of this work should be acknowledged. First, most of our data come from biological males, which is likely due to a self-selection bias related to our inclusion criterion requiring short hair over the left temporal region where we placed the ultrasound transducer. While we do not have reason to believe that the effects of sonication on brain tissue should differ between biological sexes, future work should take greater care in recruiting a representative sample. In addition, the projected focus of the ultrasound beam achieved with our single-element, fixed-focal-length transducer might also deposit energy in structures adjacent to our targets. While the behavioral effects we observe after sonication in the different conditions are consistent with the presumed roles of our target regions, we cannot entirely rule out contributions of modulating neighboring tissue. Finally, our neuroanatomical targets were selected, a priori, in a template space, and then projected onto individual anatomy. On one hand, this approach allowed us to select sites that, while achieving our scientific goals, could minimize the possibility of beam overlap in sonicated tissue across conditions. On the other hand, it is possible that projected targets might have been in suboptimal locations, something that would have worked against us. Over the last few years, a growing number of techniques can be used to segment single-subject thalamic nuclei by employing structural and/or functional magnetic resonance imaging data^43^. While this would still require a strategy for selecting an exact target within the obtained single-subject segmentations (which may exceed the focus of the ultrasound beam), it is possible that this strategy could yield stronger results.

The present work joins a rapidly growing body of research applying TUS to the healthy human brain. Several behavioral effects have been reported during sonication with TUS (“online” effects), including, for example, reduced reaction times during sonication of primary motor cortex^44^, induction of tactile sensations^45^ and improvement of sensory discrimination of tactile stimuli^46^ during sonication of primary somatosensory cortex, worsened performance on tactile discrimination during sonication of sensory thalamus^47^, and phosphene perception during sonication of visual cortex^48^. In addition, improved response inhibition has been observed during inferior frontal gyrus sonication^49^, while dorsolateral prefrontal cortex sonication decreased errors in an anti-saccade task^50^. Few studies, however, report behavioral effects following sonication (“offline”), as done in this work. Among these, alterations of mood have been reported for up to 30 min after targeting the frontal cortex with TUS^51^, increased behavioral responsiveness has been observed following thalamic stimulation in patients with a disorder of consciousness^52,53^, and one study reported altered pain thresholds for up to 10 minutes following anterior thalamic sonication^54^. Taken together, most studies applying TUS in healthy volunteers to date report the online behavioral effects of delivering TUS to one target region in healthy volunteers.

Expanding upon these previous studies, we demonstrate that single-element TUS at the parameters we apply likely has suppressive effects on the targeted regions^25–27^, is sufficient to alter behavior in healthy volunteers over a period of at least 20 min after sonication (“offline”), and is spatially precise enough to elicit distinct behavioral changes when applied to different parts of the thalamus in the same individuals. Consistent with the view that our TUS parametrization is suppressive in nature^25–27^, we found that sonication of the central thalamus led to reduced behavioral markers of alertness, whereas targeting the pulvinar resulted in selective impairments in visuospatial attention. Importantly, the effects appeared to persist for at least 20 min after sonication. Indeed, each behavioral task lasted 10 min, and task order was counterbalanced across participants. Yet, there was minimal variation attributable to task order (see the Supplementary Information), and no evidence of waning effects.

## Methods

### Participants

Right-handed and English-speaking adults (18 years of age or older) with normal or corrected-to-normal vision were recruited following procedures approved by the Institutional Review Board (IRB) at the University of California, Los Angeles (UCLA). All ethical regulations relevant to human research participants were followed. Individuals were excluded from participation if they self-disclosed current or recent use of any psychoactive medications or recreational drugs (e.g., antidepressants or stimulants), a contraindication for entering the magnetic resonance imaging (MRI) environment (e.g., MR-incompatible implants), possible or planned pregnancy (in the short term), or had hair longer than a ¼ in. over their left temporal region and were unwilling to shave it. We required short hair over the left temporal region where the ultrasound transducer would be placed to minimize air bubbles in the acoustic coupling medium that could disrupt the ultrasound^55^. Participants provided written informed consent and were compensated at a rate of $40 per hour for their participation. If participants voluntarily withdrew before completing the full protocol (loss to follow-up), then a new participant was recruited to replace them. Twenty-seven participants completed the entire protocol and are included in the analyses (age = 23.07 ± 3.33, two females), which exceeds or is comparable to other TUS work^44–54^. Thirty-seven participants consented. Ten voluntarily withdrew from the study, including three after the MRI visit and seven after at least one sonication session. No adverse reactions or discomfort related to the TUS were reported by participants who completed the entire protocol or who voluntarily withdrew.

### Design

A 3×2 within-subjects design was used to test whether targeting the pulvinar or central thalamus with TUS would affect behavioral markers of vigilance during the Psychomotor Vigilance Task (PVT)^23^ or visuospatial attention during the Egly-Driver Task (EDT)^24^. Participants underwent a magnetic resonance imaging (MRI) visit followed by three main sonication sessions, including one per ultrasound condition: sham, pulvinar, and central thalamic sonication with TUS. Session order was counterbalanced across participants, and participants were blinded to the ultrasound condition assigned to each session. Sonication sessions were held at least 24 h apart but around the same time of day (within two hours) for each participant to minimize potential individual differences in task performance between sessions that might arise from extraneous factors like circadian phase. The PVT and EDT were administered before and after sonication in every session, and in a counterbalanced order across participants.

### Magnetic resonance imaging (MRI)

MR data were acquired with a 3T Siemens Prisma fit MRI scanner in the Staglin Center for Cognitive Neuroscience at UCLA. We acquired an anatomical (T1-weighted) image for every participant using a T1-weighted MPRAGE sequence with the following parameters: TR = 2400 ms, TI = 1060 ms, TE = 2.12 ms, flip angle = 8°, voxel size = 1 × 1 × 1 mm, matrix size = 256 × 256, 192 slices. The raw T1-weighted data were converted from DICOM to NIfTI format using *dcm2niix*^56^. The anatomical image was acquired to use with a frameless stereotactic neuronavigation system to refer to when aiming and administering TUS.

#### TUS target regions

We targeted the central thalamus because of its association with arousal^10^ and the pulvinar for its role in visuospatial attention^14^. Structures in the left hemisphere were targeted in all participants. Masks for each target region were generated in standard Montreal Neurological Institute (MNI) space by drawing 5-mm spheres over the predetermined target voxels (see Fig. 1E). Since we targeted the left central thalamus and the left pulvinar in the same individuals, we defined the target region for the central thalamus over more anterior aspects of the left central thalamus to maximize the distance between the target regions defined for the central thalamus and pulvinar and avoid co-modulation of our ultrasound targets. We created TUS target region masks for each participant by warping the left pulvinar and the left central thalamus target regions defined in standard structural (MNI) space onto the participant’s T1-weighted image using *applywarp* in the FMRIB Software Library (FSL; https://fsl.fmrib.ox.ac.uk^57^). Linear and non-linear registrations between native structural (T1) and standard structural (MNI) images were performed using *flirt* and *fnirt* in FSL. All steps were visually inspected and adjusted as needed to ensure anatomical accuracy and plausibility. The TUS target region masks were created for each participant to be overlaid on their individual T1 image in a neuronavigational system to refer to when aiming and delivering the TUS.

### Transcranial ultrasound stimulation (TUS)

Low-intensity, focused TUS was administered using a BXPulsar 1002 drive system and an accompanying fixed-focal-length, single-element, air-backed spherical transducer with an 80-mm fixed focal length, 61-mm aperture, and 650-kHz operating frequency (Brainsonix Corp., USA)^58^. A Brainsight neuronavigation system (Rogue Research, Inc., Canada) was retrofitted for concurrent use with TUS. Optical trackers placed on the participant’s head and a custom ultrasound transducer holder were co-registered with the participant’s anatomical T1 image in the Brainsight system, allowing us to visualize the transducer’s position relative to the participant’s brain in real time. Sham sonication was achieved by placing a pigmented gel pad that absorbs sound (Brainsonix Corp., USA) between the surface of the transducer and the participant’s head, preventing ultrasound transmission to the brain while still allowing for auditory and tactile sensations from TUS^59^. The transducer was always placed over the left temporal region to reach structures in the left hemisphere and acoustic coupling gel pads (Brainsonix Corp., USA) that promote ultrasound transmission were used during pulvinar and central thalamic sonication^58^, making setup identical between the sham and real sonication sessions. During sham sonication, the transducer was aimed at either the left pulvinar or the left central thalamus in counterbalanced order across participants. Since setup and procedure were identical in all ultrasound conditions and ultrasound sessions were held at least 24 h apart, we minimized the likelihood that participants could detect differences between the ultrasound conditions.

#### Sonication parameters

Ultrasound was delivered in an on/off block design with 10 blocks of 30 s of rest and 30 s of sonication with the following parameters: pulse duration (PD) = 0.5 ms, pulse repetition interval (PRI) = 10 ms, duty cycle (DC) = 5%, pulse repetition frequency (PRF) = 100 Hz, I_spta.3_ $\approx \,$72 mW/cm^2^, I_sppa.3_ ≈ 14.40 W/cm^2^, which comply with the United States Food and Drug Administration (FDA) guidelines for diagnostic ultrasound^60^ and have no known adverse thermal bioeffects^61^. These specific pulsation parameters were derived from prior work in the rodent model demonstrating that thalamic sonication at this parametrization modulates extracellular cortical GABA levels^25^ resulting in broad neural inhibition^26^. The suppressive nature of this specific parametrization is also supported by a large meta-analysis of the relationship between TUS parametrization and its neuromodulatory valence, which concluded that the only parameter systematically associated with the valence of the sonication was duty cycle, with duty cycles ≤10% typically associated with suppressive effects^27^. Indeed, previous work targeting the thalamus in patients with a disorder of consciousness^53^ and anterior parts of the thalamus and the globus pallidus in healthy volunteers^28^ showed brain-wide decreases in blood-oxygen-level–dependent signal during sonication at these same parameters in the targeted regions as well as connected subcortical and cortical areas.

#### Procedure

The individual T1 image and TUS target region masks were opened in the Brainsight neuronavigation system. The participant’s head was secured in a comfortable position using chin and head rests, and they were instructed to remain as still as possible to minimize movement during TUS positioning and administration. The transducer was first placed over the left temple (approximately 1/3 of the distance from the corner of the left eye to the left tragus and superior to that point by 2 cm), which is the thinnest part of the temporal bone and the ideal cranial entry point for minimizing ultrasound disruption by the skull. Deviations in transducer position were made iteratively until the projected trajectory of the ultrasound beam passed through the target region. The transducer was kept as flat against the side of the head as possible to deliver ultrasound perpendicular to the skull surface and minimize the scattering, reflection, or refraction of the ultrasound. Once an adequate transducer position was achieved, aqueous ultrasound gel (Aquasonic Clear Ultrasound Transmission Gel; Parker Laboratories, Inc., USA) was applied to the region of the scalp subsuming the diameter of the transducer such that no hair protruded from the gel layer and visible air bubbles were smoothed out^55^. A thin layer of ultrasound gel was also applied between the surface of the transducer and the gel pad assigned to the session, with visible air bubbles pressed out. If reaching the target region required tilting the transducer relative to the participant’s head, then an angled gel pad that filled the expanding gap between the transducer and the scalp was used. Additional ultrasound gel was applied to fill any remaining concavities between the transducer and the scalp before securing the transducer into position. Transducer position was verified continuously throughout the sonication period and adjustments were made as needed to ensure that the projected trajectory of the ultrasound beam passed through the target region.

#### Acoustic modeling

Acoustic modeling was performed using BabelBrain, an open-source application for TUS modeling^62^. BabelBrain allows for the simulation of the acoustic effects achieved with a given ultrasound transducer trajectory (location and orientation of the transducer toward a target region) for an individual brain, considering the type of transducer used, the distance from the transducer surface to the scalp, sonication parameters, and distortion effects caused by the skull (estimated automatically by BabelBrain from the individual T1-weighted image). The specific fixed-focal-length Brainsonix transducer (80 mm) used in this work is supported in BabelBrain.

### Apparatus and stimuli

Stimuli and tasks were created and administered using PsychoPy (Version 2021.2.3^63^) on a monitor with a 60 Hz refresh rate. Participants were seated in an upright position 150 cm away from the monitor with a button box in their right hand. Earplugs were provided to reduce auditory distractions during the behavioral tasks.

#### Psychomotor vigilance task (PVT)

The PVT assesses the capacity to maintain vigilance (also called sustained attention) over uninterrupted periods^23^. Participants maintained central fixation and pressed a button as quickly as possible after a visual cue appeared for 10 min without breaks. The visual cue was a yellow millisecond counter that appeared inside of a red rectangle at the center of the monitor at random 2–10 s inter-trial intervals (see Fig. 1B). Response time was recorded on every trial. When the participant pressed the button, the millisecond counter stopped but remained on the screen for one second, displaying the response time recorded on that trial to the participant. If the participant pressed the button before the visual cue appeared or too quickly after it appeared (≤100 ms), then a false start message was presented. If the participant did not respond within 30 seconds of the visual cue, then the trial timed out and a beep played to alert the participant. Many behavioral outcomes can be derived from response times recorded during the PVT. We examined mean response time, the slowest 10% of responses, response speed (1/response time), and lapses in attention (unusually slow responses) because of their sensitivity to states of diminished arousal like sleep deprivation^29,30^. We also examined the time-on-task effect (also called a vigilance decrement), whereby responses slow and lapses become more frequent over the duration of the task due to factors like task fatigue or boredom^36^ because they are exaggerated under states of diminished arousal like sleep deprivation^29,30^.

#### Egly-Driver task (EDT)

The EDT is a visuospatial attention task that assesses the ability to correctly and promptly respond to validly and invalidly cued visual targets that appear in various spatial locations^24^. Participants maintained central fixation and pressed a button if they detected a visual target for 10 min. A 30-s break was provided every 2 min. Trial onset was marked by the presentation of two horizontal white bars, one above fixation and another below it. After a variable 400–800 ms delay, a spatial cue (black square) was presented for 100 ms at the end of one of the white bars to direct the participant’s attention to that location. On main trials (90% of trials), a visual target (change in contrast) appeared at the end of one of the white bars for 100 ms either a short (300–350 ms) or long (1000–1050 ms) delay after the cue. The visual target could appear in the cued location or another (uncued) location, but the cue indicated the location where the target was most likely to appear (with 75% cue validity). The trial ended after a 150–700 ms variable response window. On catch trials (10% of trials), no target appeared, and the trial ended 450–1750 ms after the cue.

Participants could be either correct (“hits” on main trials and “correct rejections” on catch trials) or incorrect (“misses” on main trials and “false alarms” on catch trials) on an EDT trial. Response time was recorded on main trials when the participant responded. If the participant pressed the button on a main trial before the target appeared or too soon afterward (≤100 ms), then the response was considered anticipatory and discarded. The visual target (change in contrast) was selected for each participant using a staircase procedure that was identical to the main task, except that the contrast of the target changed on every trial. The staircase followed a two-down and one-up rule whereby successful trials were followed by more difficult ones, decreasing the contrast of the target relative to the white bar until the participant failed to detect it, and then adjusting accordingly. The staircase ended when we identified a minimum contrast with which the participant correctly detected the visual target on 50% of the trials. The resulting contrast was saved and used for all EDT blocks.

Spatial attention is associated with enhanced sensory processing and behavioral responses for stimuli that appear in the attended region. For the EDT, participants typically detect the visual target more often (and respond to it more quickly) when the spatial cue correctly indicated its location than when the spatial cue was invalid, demonstrating the behavioral benefit of spatial attention and the behavioral cost of disengaging attention from a cued location to respond to the target^24^. By varying cue validity and the visual hemifield that the spatial cue and visual target appear in, we can also examine hemispheric differences in the behavioral benefits and costs associated with visuospatial attention using the EDT. Indeed, the original EDT paper includes an experiment in which differences in the cost associated with invalid cueing in patients with parietal lesions are tested^24^. Some EDT versions use cue-to-target intervals selected from a continuous range of values between 300 and 1100 ms^18^. We administered an abbreviated version that presented either short (300–350 ms) or long (1000–1050 ms) intervals between the cue and target to reduce EDT task duration, avoiding the introduction of differences in task duration between the PVT and EDT. Since we counterbalanced task order across participants and we did not know how long the behavioral effects of sonication in the different conditions would last, introducing differences in task duration between the tasks could limit our ability to detect behavioral changes after TUS or introduce variability in the results that would limit our conclusions. Still, the abbreviated version still allows us to determine whether sonication in any of the conditions affects EDT performance, and whether there are hemispheric effects of sonication on visuospatial attention.

### Statistics and reproducibility

Mixed-effects modeling was used because of the repeated-measures structure of our data, but also because it allows for the specification of random effects. Mixed-effects modeling was performed using the lme4 (Version 1.1.36^64^), lmerTest (Version 3.1.3^65^), emmeans (Version 1.11.0^66^), and DHARMa (Version 0.4.7^67^) packages in R (Version 4.4.3; R Core Team^68^). Data cleaning and visualization were completed in Python (Version 3.11.11). To better isolate the effects of sonication, we included covariates for biological sex and age as well as random effects for participant and task order in all models. Since participants underwent the ultrasound conditions in separate sessions held on different days, we included a random intercept for participant with a random slope for ultrasound condition (1 + condition | participant). The random intercept for participant accounts for having multiple observations per participant, since we use unaggregated data from a repeated-measures design. The random slope of condition captures how the effect of ultrasound condition varies for each participant when all other fixed effects are at their reference levels (including block at pre-sonication), and thus accounts for individual differences in task performance before sonication between the sessions (and thus conditions) that could interfere with estimates of the fixed effects in the models. The PVT and EDT were administered before and after sonication in every session, but in a counterbalanced order across participants, separating participants into two groups based on task order (PVT then EDT or EDT then PVT). So, we included a random intercept for task order (1 | task order) to capture differences in task behavior that could be attributed to the order in which participants completed the tasks.

#### Psychomotor vigilance task (PVT)

Mixed-effects models were used to examine whether sonication in any of the ultrasound conditions affected behavioral markers of vigilance during the PVT, specifically response time, the slowest 10% of responses, response speed, and lapses in attention. These outcomes were selected because of their sensitivity to states of diminished arousal like sleep deprivation^29^. For the slowest 10% of responses, we considered only response times in the slowest 10% from each PVT block per participant. Response speed is defined as 1/RT, whereby the response time (in ms) on every trial was divided by 1000 and then reciprocally transformed. Lapses refer to lapses in attention and are typically defined as response times greater than 500 ms or twice the mean response time for an individual participant^29^. However, our participants never or rarely had response times greater than 500 ms or twice their mean response time (see the Supplementary Fig. 6). So, instead, we combined the unaggregated data from all PVT blocks for each participant and considered response times greater than that participant’s mean response time plus two standard deviations to be lapses in attention.

##### Data cleaning

Participants completed 210.59 trials (SD = 5.29, min = 197, max = 226) per PVT block, on average. Individual trials were discarded if the response time recorded on that trial was a false start (≤100 ms) or a definite outlier relative to the other trials for that PVT block and participant based on Tukey’s method (less than the first quartile minus 3 × the IQR or greater than the third quartile plus 3 × the IQR)^69^. Participants had 6.92 false starts (SD = 4.72, min = 1, max = 25) and 6.06 outliers (SD = 3.55, min = 1, max = 17) per PVT block, on average, leaving 197.42 trials per block after cleaning (SD = 7.33, min = 176, max = 214), on average. A summary of trial cleaning results is provided in Supplementary Table 1. Out of the 1184.52 trials (SD = 31.22, min = 1122, max = 1243) retained for each participant across all PVT blocks, 55.15 (SD = 7.98, min = 40, max = 69) were considered lapses in attention, on average.

To ensure that the mixed-effects model results for an outcome could not be driven by erroneous changes in the outcome in only some participants, we checked each mixed-effects model for influential participants and excluded outliers in any ultrasound condition at the group level. For each mixed-effects model, we assessed the influence of individual participants on model estimates by calculating the Cook’s distance for each participant. Participants with a Cook’s distance substantially greater than the group (>4 × the mean across participants) were considered influential and their data were examined further. If these participants were also definite outliers in their change in the model’s outcome across blocks (from before to after sonication) in any of the ultrasound conditions based on Tukey’s method (less than the first quartile minus 3 × the IQR or greater than the third quartile plus 3 × the IQR)^69^, then their data from that condition was left out of the final mixed-effects model run for the outcome. All other data from these participants were included in the models to retain as much data as possible and improve estimates of the random effects. Based on these criteria, one participant surpassed the Cook’s distance threshold in the models examining response time (see Supplementary Fig. 1a, b) and response speed (see Supplementary Fig. 1e, f). Upon further examination, this participant was also a definite outlier in their change in the outcomes across blocks in the central thalamus session, so their data from this condition was left out. Three participants surpassed the Cook’s threshold in the model for the slowest 10% of responses, and they were also definite outliers in their change in the outcome across blocks in at least one ultrasound condition (see Supplementary Fig. 1c, d). No participants exceeded the Cook’s threshold in the model for lapses in attention (see Supplementary Fig. 1g). Descriptive statistics for the cleaned PVT data used in the models are presented in Fig. 2 and Supplementary Fig. 2.

##### Analysis

To examine whether sonication in any of the ultrasound conditions affected response time, the slowest 10% of responses, response speed, or lapses in attention during the PVT, we fit mixed-effects models testing the three-way interaction between the effects of ultrasound condition (sham, pulvinar, and central thalamic sonication), block (before and after sonication), and time into the block (in minutes) on each outcome. All models shared the following specification: outcome ~ condition × block × minute + sex + age + (1 + condition | participant) + (1 | task order). This model specification allows us to quantify how each PVT outcome changed from before to after sonication in the different conditions on average, but also over the duration of the task (per minute into the task block). Linear mixed-effects models were used for response time, the slowest 10% of responses, and response speed because they are continuous variables in the unaggregated data. A mixed-effects logistic regression was used for lapses because an individual trial was either considered a lapse in attention or not, making lapses a binary variable in the unaggregated data. Estimated marginal means contrasts were used to make additional comparisons not included in the fixed effects, and adjusted for multiple comparisons using the Benjamini-Hochberg method^70^. Model output summaries, estimated marginal means, and estimated marginal means contrasts made for each mixed-effects model are presented in Supplementary Figs. 3–7 and Supplementary Tables 2–13.

#### Egly-Driver task (EDT)

Mixed-effects models were used to examine whether targeting the pulvinar or central thalamus with TUS affected visuospatial attention during the EDT^24^, including correct responses and response times. Separate mixed-effects logistic regressions were used to assess whether the effects of sonication on correct responses depended on trial type (considering all trials), spatial cue location on catch trials, or spatial cue validity and visual target hemifield on main trials. A linear mixed-effects model was used to examine whether the effects of sonication on response times from main trials depended on spatial cue validity and visual target hemifield. For main trial analyses, we considered all trials with validly cued visual targets but restricted trials with invalidly cued visual targets to those in which the spatial cue and visual target appeared in opposite sides of the visual field. This separated main trials into four types according to spatial cue validity and visual target location: validly cued ipsilateral targets, invalidly cued ipsilateral targets, validly cued contralateral targets, and invalidly cued contralateral targets (see Supplementary Fig. 8 for a visualization).

##### Data cleaning

Participants completed 216.07 main trials (SD = 3.93, min = 205, max = 227) and 23.93 catch trials (SD = 3.93, min = 13, max = 35) per EDT block, on average. On catch trials, the spatial cue appeared ipsilaterally in 13.07 (SD = 2.91, min = 6, max = 21) trials and contralaterally in 10.86 (SD = 2.52, min = 4, max = 17) trials per EDT block, on average. On main trials, participants were shown 88.20 validly cued ipsilateral visual targets (SD = 6.49, min = 72, max = 101), 15.28 invalidly cued ipsilateral targets (SD = 3.06, min = 7, max = 23), 87.33 validly cued contralateral targets (SD = 6.74, min = 64, max = 101), and 12.13 invalidly cued contralateral targets (SD = 2.78, min = 6, max = 23) per EDT block, on average. Main trials with a recorded response time ≤100 ms were considered anticipatory false starts and discarded. Participants submitted 1.83 false starts (SD = 3.35, min = 0, max = 20) per EDT block, on average. We are missing data from four EDT blocks due to either button box failure or incorrect button presses. We are missing data from the post-sonication EDT block in the sham condition from one participant. For another participant, we are missing button presses from the pre- and post-sonication EDT blocks in the pulvinar session, and from the pre-sonication block in the sham session. The rest of the data from these participants show no systematic issues compared to the group (see Supplementary Fig. 9a), so the rest of their data were included in the mixed-effects models to retain as much data as possible and improve estimates of the random effects.

To ensure that the mixed-effects model results for an outcome could not be driven by erroneous changes in the outcome in only some participants, we checked each mixed-effects model for influential participants and excluded outliers in any ultrasound condition at the group level. For each mixed-effects model, we assessed the influence of individual participants on model estimates by calculating the Cook’s distance for each participant. Participants with a Cook’s distance substantially greater than the group (>4 × the mean across participants) were considered influential and their data were examined further. If these participants were also definite outliers in their change in the model’s outcome across blocks (from before to after sonication) in any of the ultrasound conditions based on Tukey’s method (less than the first quartile minus 3 × the IQR or greater than the third quartile plus 3 × the IQR)^69^, then their data from that condition was left out of the final mixed-effects model run for the outcome. All other data from these participants were included in the models to retain as much data as possible and improve estimates of the random effects. One participant surpassed the Cook’s threshold in the model comparing correct responses between trial types and was also a definite outlier in their change in the outcome across blocks in the sham session (see Supplementary Fig. 9a, c). One participant surpassed the Cook’s threshold in the model examining correct responses on catch trials but was not an outlier in their change across blocks in the sham session, so their data were kept in the model (see Supplementary Fig. 9d, e). One participant surpassed the Cook’s threshold in the model examining correct responses on main trials and was also an outlier in their change in the outcome across blocks in the sham session (see Supplementary Fig. 9f, g). No participants surpassed the Cook’s threshold in the response time model (see Supplementary Fig. 9h). Descriptive statistics for the cleaned EDT data used in the mixed-effects models are presented in Fig. 3 and Supplementary Fig. 10.

Since participants were provided with a variable response window on main trials during the EDT, the natural response time distribution differed by design and trial cleaning based on response time using Tukey’s method was not applicable. Instead, to ensure that mixed-effects modeling results for response time could not be driven by erroneous response times, we opted for a model-based approach in identifying outlier observations using the standardized residuals in the linear mixed-effects model examining response time. Specifically, we labeled observations as outliers if their standardized residual exceeded ± 4 standard deviations from zero, which indicates that the observation was extreme relative to the patterns expected by the model for that participant and condition (see Supplementary Fig. 14). Out of the 25842 observations in total, 138 were labeled as outliers and left out of the final mixed-effects model. Descriptive statistics for the cleaned response time data used in the models are presented in Fig. 3D and Supplementary Fig. 10d.

##### Analysis

Participants could be either correct (“hits” on main trials and “correct rejections” on catch trials) or incorrect (“misses” on main trials and “false alarms” on catch trials) on an individual EDT trial, making correct responses a binary variable in the unaggregated EDT data. Separate mixed-effects logistic regressions were used to assess whether the effects of sonication on correct responses depended on trial type (considering all trials), spatial cue location on catch trials, or spatial cue validity and visual target hemifield on main trials. A linear mixed-effects model was used to examine whether the effects of sonication on response times from main trials depended on spatial cue validity and visual target hemifield, as response time was a continuous variable in the unaggregated EDT data recorded only on main trials when there was a response.

##### Correct responses

To test whether sonication in any of the ultrasound conditions affected correct responses during the EDT, and whether this differed for catch and main trials, we fit a mixed-effects logistic regression testing the three-way interaction between the effects of ultrasound condition (sham, pulvinar, and central thalamic sonication), block (before and after sonication), and trial type (catch or main) on correct responses with the following specification: correct responses ~ condition × block × trial type + sex + age + (1 + condition | participant) + (1 | task order). All EDT trials were considered in this model. Descriptive statistics for the underlying raw data used in the model are presented in Fig. 3A and Supplementary Fig. 10a. A summary of the model results is presented in Supplementary Fig. 11 and Supplementary Table 14. Estimated marginal means contrasts were used to make additional comparisons in the model not included in the fixed effects and adjusted for multiple comparisons using the Benjamini-Hochberg method^70^. All estimated marginal means and contrasts performed are presented in Supplementary Tables 15 and 16, respectively.

To examine whether the effects of sonication in the different conditions on correct responses during catch trials (“correct rejections”) depended on whether the spatial cue appeared ipsilaterally or contralaterally to the sonicated region, we fit a mixed-effects logistic regression testing the three-way interaction between the effects of ultrasound condition (sham, pulvinar, and central thalamic sonication), block (before and after sonication), and spatial cue location (ipsilateral or contralateral) on correct responses on catch trials with the following specification: correct responses ~ condition × block × cue location + sex + age + (1 + condition | participant) + (1 | task order). Descriptive statistics for the underlying raw data used in the model are presented in Fig. 3B and Supplementary Fig. 10b. A summary of the model results is presented in Supplementary Fig. 12 and Supplementary Table 17. Estimated marginal means contrasts were used to make additional comparisons in the model not included in the fixed effects and adjusted for multiple comparisons using the Benjamini-Hochberg method^70^. Estimated marginal means and contrasts performed are presented in Supplementary Tables 18 and 19, respectively.

Lastly, to examine whether the effects of sonication in the ultrasound conditions on correct responses during main trials (“hits”) depended on spatial cue validity and visual target location, we fit a mixed-effects logistic regression testing the three-way interaction between the effects of ultrasound condition (sham, pulvinar, and central thalamic sonication), block (before and after sonication), and cue-target location (validly cued ipsilateral target, invalidly cued ipsilateral target, validly cued contralateral target, and invalidly cued contralateral target) on correct responses with the following specification: correct responses ~ condition × block × cue-target location + sex + age + (1 + condition | participant) + (1 | task order). We considered all main trials with validly cued visual targets but restricted main trials with invalidly cued visual targets to those in which the spatial cue and visual target appeared in opposite sides of the visual field, separating main trials into four types according to spatial cue validity and visual target location: validly cued ipsilateral targets, invalidly cued ipsilateral targets, validly cued contralateral targets, and invalidly cued contralateral targets (see Supplementary Fig. 8 for a visualization). Descriptive statistics for the underlying raw data used in the model are presented in Fig. 3C and Supplementary Fig. 10c. A summary of the model results is presented in Supplementary Fig. 13 and Supplementary Table 20. Estimated marginal means contrasts were used to make additional comparisons in the model not included in the fixed effects and adjusted for multiple comparisons using the Benjamini-Hochberg method^70^. Estimated marginal means and contrasts are presented in Supplementary Tables 21 and 22, respectively.

##### Response time

To examine whether the effects of sonication in the different ultrasound conditions on response times from main trials depended on spatial cue validity and visual target location, we fit a linear mixed-effects model testing the three-way interaction between ultrasound condition (sham, pulvinar, and central thalamic sonication), block (before and after sonication), and cue-target location (validly cued ipsilateral target, invalidly cued ipsilateral target, validly cued contralateral target, and invalidly cued contralateral target) on response times from main trials with the following specification: response time ~ condition × block × cue-target location + sex + age + (1 + condition | participant) + (1 | task order). We considered all main trials with validly cued visual targets but restricted main trials with invalidly cued visual targets to those in which the spatial cue and visual target appeared in opposite sides of the visual field, separating main trials into four types according to spatial cue validity and visual target location: validly cued ipsilateral targets, invalidly cued ipsilateral targets, validly cued contralateral targets, and invalidly cued contralateral targets (see Supplementary Fig. 8 for a visualization). Descriptive statistics for the underlying raw data used in the model are presented in Fig. 3D and Supplementary Fig. 10d. A summary of the model results is presented in Supplementary Fig. 14 and Supplementary Table 23. Estimated marginal means contrasts were used to make additional comparisons in the model not included in the fixed effects and adjusted for multiple comparisons using the Benjamini-Hochberg method^70^. Estimated marginal means and contrasts performed are presented in Supplementary Tables 24 and 25, respectively.

### Reporting summary

Further information on research design is available in the Nature Portfolio Reporting Summary linked to this article.

## Supplementary information

Transparent Peer Review file

Supplementary Information

Description of Additional Supplementary files

Supplementary Data 1

Supplementary Data 2

Reporting Summary

## Data Availability

The raw anonymized data from this work are available at the OSF repository associated with this publication: https://osf.io/pn8eq/overview?view_only=5f6b10f6638d4d8db56ece1b05c5a972. Numerical source data for all graphs in the manuscript can be found in Supplementary Data 1 and 2. All other data are available from the corresponding author upon reasonable request.

## Code availability

No custom code or mathematical algorithms were created for this work. Stimuli and tasks were created and administered using PsychoPy (Version 2021.2.3^63^). Mixed-effects modeling was performed using the lme4 (Version 1.1.36^64^), lmerTest (Version 3.1.3^65^), emmeans (Version 1.11.0^66^), and DHARMa (Version 0.4.7^67^) packages in R (Version 4.4.3; R Core Team^68^). Data cleaning and visualization were completed in Python (Version 3.11.11).
