# Supplementary material for: Targeting central thalamus with transcranial ultrasound reduces arousal in healthy volunteers
Source: Commun Biol. 2026 Oct 26;9:1403. doi: 10.1038/s42003-026-10637-7 (PMC13710743; doi:10.1038/s42003-026-10637-7)
Supplement: Supplementary file 1 [file 42003_2026_10637_MOESM1_ESM.pdf]

nature portfolio

Peer Review File

Targeting central thalamus with transcranial ultrasound reduces arousal in healthy volunteers

Corresponding Author: Ms Amber Hopkins

This file contains all reviewer reports in order by version, followed by all author rebuttals in order by version.

Version 0:

Reviewer comments:

Reviewer #1

(Remarks to the Author)
Dear Dr. Hopkins,
Thank you for taking the time to respond and address the outlined comments. I believe the updates to the paper have improved its clarity. Below are outstanding comments and suggestions that I believe still need to be addressed (broken down by general concern).

Blinding of participants

Thank you for the suggestion. We have added some additional about this to the methods section (see lines 580-589). Participants were not asked after TUS what stimulation condition they were placed in.
Thank you for clarifying. Adding a section on the limitations of blinding checks appears warranted. The absence of a blinding check limits readers' confidence that participants were unaware of condition assignment. Potentially adding previous literature that argues this is unnecessary for TUS would suffice.

Sample Size

Thank you for the suggestion. We have added it to the manuscript (see lines 521-522).
Thank you for adding more information. I do think that saying 37 were consented, which is sufficient, is misleading since 27 were included in analyses. If this is incorrect, however, then please fix the language in the paper. While previous research using TUS is small (~10 in some studies), I do think a formal post hoc power analysis would be warranted to strengthen this section. At the very least, discussing expected effect sizes based on previous research should be added to this section to strengthen the methods.

EDT explanation

There is explicit discussion about the rationale for this in the methods section (see lines 708-718).
Since tasks were counterbalanced, sensitivity analyses showing that effects of condition order did not have an effect could potentially address your comment of: “we did not know how long the effects of sonification in different conditions would last” sentence in line 712. Additionally, because of how this section is currently phrased, it might be better to discuss this limitation more thoroughly in the limitations section.

Lapses in attention

Lapses in attention are a common outcome of the PVT but are typically defined as response times greater than 500 ms or twice the mean response time for an individual participant. However, our participants never or rarely had response times greater than 500 ms or twice their mean response time (see the Supplementary Fig. 6). So, instead, we combined the unaggregated data from all PVT blocks for each participant and considered response times greater than that participant’s mean response time plus two standard deviations to be lapses in attention. This is discussed in lines 749-755.

I appreciate the clarification; however, if lapses in attention are common in the PVT and you are not seeing the typical RT effects in your own PVT experiment, this is potentially very concerning. Has a previous study also not shown typical PVT

RTs and combined data this way? I am concerned that this study is creating a new variable called “lapses in attention” simply because you aren’t seeing “typical” lapses in attention. Without a stronger justification, this effect seems to be “unusually slower RT” rather than lapses in attention. You do point this out earlier in the paper, and the paper might benefit from rephrasing the argument away from a lapses-in-attention framework.

Supplemental Materials Length

Thank you for the suggestion. We acknowledge that the supplemental material is extensive; however, we report these analyses to ensure transparency and to allow interested readers access to comparisons not included in the main text. Biological sex was included as a covariate in all models in accordance with editorial guidelines on sex and gender reporting. As noted, biological sex did not affect model outputs, but we retained it per editorial request.

This is appreciated; however, could critical supplemental materials be included with extended outputs in OSF? The supplementary section is currently hard to navigate and would benefit from highlighting more critical results.

Open Access This Peer Review File is licensed under a Creative Commons Attribution 4.0 International License, which permits use, sharing, adaptation, distribution and reproduction in any medium or format, as long as you give appropriate credit to the original author(s) and the source, provide a link to the Creative Commons license, and indicate if changes were made.
In cases where reviewers are anonymous, credit should be given to 'Anonymous Referee' and the source.
The images or other third party material in this Peer Review File are included in the article’s Creative Commons license, unless indicated otherwise in a credit line to the material. If material is not included in the article’s Creative Commons license and your intended use is not permitted by statutory regulation or exceeds the permitted use, you will need to obtain permission directly from the copyright holder.
To view a copy of this license, visit https://creativecommons.org/licenses/by/4.0/

Reviewer #2 (Remarks to the Author):

I agree the advantages of adopting healthy subjects, within-subject design, and tFUS in this study. The major conclusions of this study are about the causal role of the central thalamus in vigilance and the role of the pulvinar in visuospatial attention, while similar conclusions have been reached or supported in previous studies. In my opinion, conceptual advance provided by the paper is not of a high enough caliber for Nature Communications.

To the best of our knowledge, no previous work has demonstrated the causal roles of the central thalamus in vigilance and the pulvinar in visuospatial attention in the healthy human brain. To date, these functional associations have only been supported by correlational studies in healthy volunteers and causal studies (lesion/invasive stimulation work) with human patient populations or non-human animal models. Our work provides the first within-subject, reversible causal manipulation of the central thalamus and pulvinar in the healthy human brain, constituting a distinct and important advance over previous work.

We believe that this comment also overlooks additional key contributions and advances to the field made by this study. Specifically, this work is the first to demonstrate that transcranial ultrasound stimulation (TUS) can elicit distinct behavioral effects when applied to different thalamic nuclei within the same individuals, and that these effects persist for at least 20 minutes without dissipating. This underscores the potential of TUS as a high-resolution, non-invasive tool for functional brain mapping in healthy humans, capable of dissociating the contributions of nearby subcortical structures.

In addition, some results are still questionable to me. In normal trials of the EDT task, reaction time changes (original Figure 3c Valid Ips and Valid Con) caused by pulvinar stimulation and sham stimulation were similar. Please clarify whether the existence of tFUS is the main difference between the Sham and Pul conditions. If true, the effect in the Pul condition are largely due to the sham stimulation. The detection rate change caused by sham stimulation were even greater than the change caused by pulvinar stimulation (original Figure 3b Valid Ips and Valid Con), raising the concerns about the sham stimulation.

It is correct that reaction time changes for validly cued trials (see Fig. 3d, first and third panels) were similar following pulvinar and sham stimulation. Importantly, however, this pattern is expected based on the established EDT literature, **which consistently shows that attentional deficits manifest selectively for invalidly cued contralateral targets, rather than for validly cued stimuli.**

Consistent with this literature, we observed slower responses to invalidly cued contralateral targets following pulvinar sonication, **relative to both sham and central thalamus conditions (Fig. 3d, fourth panel), while responses to validly cued targets were unaffected (Fig. 3d, first and third panels). This selective effect is discussed explicitly in the manuscript (lines 404–410) and mirrors the original EDT findings in patients with unilateral damage to other attention-related regions.[24]**

With respect to detection rates, while sham stimulation produced changes in some valid conditions, our central finding is that pulvinar sonication led to a relative reduction in visual detection rates compared to sham, **particularly for contralateral targets. This differential effect supports the conclusion that pulvinar stimulation specifically disrupts visuospatial attention, rather than reflecting nonspecific effects of sham stimulation.**

Reviewer #3 (Remarks to the Author):

Dear Dr. Hopkins,

Thank you for taking the time to respond and address the outlined comments. I believe the updates to the paper have significantly improved its clarity. Below are minor outstanding comments and suggestions that I believe still need to be addressed.

1. Abstract

a. All points have been addressed

2. Introduction

a. All points have been addressed

3. Methods and Materials

i. If participant blinding was completed, what was done to ensure that participants could not tell 346 the difference between conditions? It is crucial to rule out any placebo effect in this study.

ii. Thank you for the explanation. I believe that a shortened version of this should be placed in the methods section. This would strengthen your methods. To clarify, were participants asked after TUS what stimulation condition they thought they had been placed in? I am asking because I think it would further strengthen your methods to include a test showing no differences during the sessions.

Thank you for the suggestion. We have added some additional about this to the methods section (see lines 580-589). Participants were not asked after TUS what stimulation condition they were placed in.

b. Please provide some justification for the sample size

i. Thank you for the explanation. Adding a shortened version of this to the manuscript would be useful. Adding citations to other studies using similar sample sizes would also be useful. Seeing that other studies have 40 to 50 participants does cause some concern, so strengthening the reasoning for 27 participants in the paper would be preferred.

Thank you for the suggestion. We have added it to the manuscript (see lines 521-522).

c. Line 611: it is not clear to me why a shorter EDT was used and why task duration would 389 impact the results. Please provide a more detailed explanation of the reasoning here. This also 390 comes up as a concern in the discussion, and it seems like a major concern.

i. Thank you for the explanation in the letter. Adding this as a shortened version to the discussion or methods would be useful.

There is explicit discussion about the rationale for this in the methods section (see lines 708-718).

d. PVT line 671: Has this response time method been used for other studies? Please provide a bit more justification for why this was chosen here.

i. I might be misunderstanding, but you say lapses in attention have not been used before, but then provide citations in the paper. For example, you cite lapses in attention for the PVT in the introduction, “including slower responses 79 and more lapses in attention29,30”. Can you clarify

why, in your response, you say that the “lapse in attention” outcome that you are using has not been done before?

Lapses in attention are a common outcome of the PVT but are typically defined as response times greater than 500 ms or twice the mean response time for an individual participant[29]. However, our participants never or rarely had response times greater than 500 ms or twice their mean response time (see the Supplementary Fig. 6). So, instead, we combined the unaggregated data from all PVT blocks for each participant and considered response times greater than that participant’s mean response time plus two standard deviations to be lapses in attention. This is discussed in lines 749-755.

4. Results

a. All points have been addressed

5. Discussion

a. All points have been addressed

6. Supplemental

a. New suggestion: If possible, please pare down the supplemental section to help the readers interpret your results. I appreciate that you are openly sharing all your results with readers, but the amount of supplemental material is overwhelming. For example, I don’t think the male vs. female tests are needed, since there are only two females in the study. With only two in that group, I wouldn’t expect differences, and you do discuss this in your limitations section.

Thank you for the suggestion. We acknowledge that the supplemental material is extensive; however, we report these analyses to ensure transparency and to allow interested readers access to comparisons not included in the main text. Biological sex was included as a covariate in all models in accordance with editorial guidelines on sex and gender reporting. As noted, biological sex did not affect model outputs, but we retained it per editorial request.

Reviewers' comments:

Reviewer #3 (Remarks to the Author):

Dear Dr. Hopkins,
Thank you for taking the time to respond and address the outlined comments. I believe the updates to the paper have improved its clarity. Below are outstanding comments and suggestions that I believe still need to be addressed (broken down by general concern).

Blinding of participants

Thank you for the suggestion. We have added some additional about this to the methods section (see lines 580-589). Participants were not asked after TUS what stimulation condition they were placed in.
Thank you for clarifying. Adding a section on the limitations of blinding checks appears warranted. The absence of a blinding check limits readers' confidence that participants were unaware of condition assignment. Potentially adding previous literature that argues this is unnecessary for TUS would suffice.

Thank you for the comment, and we understand the concern. Using sham pads are a common approach with TUS (see e.g., https://www.sciencedirect.com/science/article/pii/S1388245725000148), and they allow for much of the same sensory stimulation as the transmitting pads. In addition, our participants were untrained in TUS and sessions were held on separate days at least 24 hours apart, so it would be very difficult for participants to remember exactly how one session felt enough to directly compare it to the next session.

Sample Size

Thank you for the suggestion. We have added it to the manuscript (see lines 521-522).
Thank you for adding more information. I do think that saying 37 were consented, which is sufficient, is misleading since 27 were included in analyses. If this is incorrect, however, then please fix the language in the paper. While previous research using TUS is small (~10 in some studies), I do think a formal post hoc power analysis would be warranted to strengthen this section. At the very least, discussing expected effect sizes based on previous research should be added to this section to strengthen the methods.

We have reworded the participant section of the methods (see main text starting at line 508) to avoid this concern. It may be worth pointing out that we state 27 participants in the abstract, so we are upfront about the participant count from the start.

EDT explanation

There is explicit discussion about the rationale for this in the methods section (see lines 708-718).
Since tasks were counterbalanced, sensitivity analyses showing that effects of condition order did not have an effect could potentially address your comment of: “we did not know how long the effects of sonification in different conditions would last” sentence in line 712. Additionally, because of how this section is currently phrased, it might be better to discuss this limitation more thoroughly in the limitations section.

Every mixed-effects model run included task order as a random effect (see text starting at line 717), which essentially performs the kind of test that you are suggesting here. Importantly, there were no differences in any outcome variable that could be explained by the order in which participants completed the tasks (as reported throughout the Supplemental Material and discussed explicitly in the discussion text starting at line 494), indicating that task order did not drive results and that we effectively controlled for task order.

Lapses in attention

Lapses in attention are a common outcome of the PVT but are typically defined as response times greater than 500 ms or twice the mean response time for an individual participant. However, our participants never or rarely had

response times greater than 500 ms or twice their mean response time (see the Supplementary Fig. 6). So, instead, we combined the unaggregated data from all PVT blocks for each participant and considered response times greater than that participant's mean response time plus two standard deviations to be lapses in attention. This is discussed in lines 749-755.

I appreciate the clarification; however, if lapses in attention are common in the PVT and you are not seeing the typical RT effects in your own PVT experiment, this is potentially very concerning. Has a previous study also not shown typical PVT RTs and combined data this way? I am concerned that this study is creating a new variable called "lapses in attention" simply because you aren't seeing "typical" lapses in attention. Without a stronger justification, this effect seems to be "unusually slower RT" rather than lapses in attention. You do point this out earlier in the paper, and the paper might benefit from rephrasing the argument away from a lapses-in-attention framework.

Lapses in attention are defined as unusually long response times (see e.g., https://academic.oup.com/sleep/article-abstract/34/5/581/2281465) and our criterion satisfies this definition. Actually, we feel that the criterion we designed may be more suited to meeting the definition because it is individually tailored to each participant, ensuring that we only capture responses that are unusually slow for that participant rather than comparing them to a flat criterion of 500 ms as originally done.

We are not too concerned by the fact that our participants did not pass the standard thresholds for lapses. We are transparent about this and explain the rationale for this criterion for lapses in attention. It could be explained by a variety of factors. For instance, we have a younger sample size than is reported in major papers (e.g., https://academic.oup.com/sleep/article-abstract/34/5/581/2281465) and reaction times tend to increase with age (see e.g., https://journals.plos.org/plosone/article?id=10.1371/journal.pone.0045759). Moreover, since the original paper defining the PVT is from the 1985 (https://link.springer.com/article/10.3758/BF03200977), perhaps the younger generation now, 40 years later, can react to stimuli on computers more quickly given their regular use.

Supplemental Materials Length

Thank you for the suggestion. We acknowledge that the supplemental material is extensive; however, we report these analyses to ensure transparency and to allow interested readers access to comparisons not included in the main text. Biological sex was included as a covariate in all models in accordance with editorial guidelines on sex and gender reporting. As noted, biological sex did not affect model outputs, but we retained it per editorial request.

This is appreciated; however, could critical supplemental materials be included with extended outputs in OSF? The supplementary section is currently hard to navigate and would benefit from highlighting more critical results.

The Supplemental Material will appear in a separate document than the original manuscript. Since it is already made, we want to be transparent about all comparisons run, and we want to ensure that readers can find specific comparisons that are not reported in the main manuscript that they might be interested in (and we cannot anticipate which they may or may not be interested in), we feel it is good to keep it as-is. The table of contents should direct readers well to the specific comparisons that they are interested in, as well as the references to the Supplemental Material throughout the methods section.
