# Supplementary material for: Targeting central thalamus with transcranial ultrasound reduces arousal in healthy volunteers
Source: Commun Biol. 2026 Oct 26;9:1403. doi: 10.1038/s42003-026-10637-7 (PMC13710743; doi:10.1038/s42003-026-10637-7)
Supplement: Supplementary file 2 [file 42003_2026_10637_MOESM2_ESM.pdf]

Targeting central thalamus with transcranial ultrasound reduces arousal in healthy volunteers

Supplementary Information

Table of Contents

Psychomotor Vigilance Task (PVT) 2
- Additional data cleaning information 2
- Additional mixed-effects modeling results 5
  - Response time 5
  - Slowest 10% of responses 9
  - Response speed 13
  - Lapses in attention 17

Egly-Driver Task (EDT) 22
- Additional data cleaning results 22
- Additional mixed-effects modeling results 25
  - Correct responses between trial types 25
  - Correct responses on catch trials 29
  - Correct responses on main trials 33
  - Response time on main trials 39

Psychomotor Vigilance Task (PVT)

Additional data cleaning information

Supplementary Table 1: Trial cleaning summary for each participant

Sub	*Kept (total)*	*Outlier (total)*	*Kept (block)*	*Outlier (block)*
1	1122	28	187.00 +/- 7.43	4.67 +/- 3.01
2	1201	30	200.16 +/- 4.12	5.00 +/- 2.19
3	1187	49	197.83 +/- 6.49	8.17 +/- 2.04
4	1141	27	190.16 +/- 6.59	4.50 +/- 3.27
5	1243	11	207.16 +/- 3.19	1.83 +/- 0.75
6	1206	41	201.00 +/- 8.25	6.83 +/- 3.11
7	1177	26	196.17 +/- 7.14	4.33 +/- 1.97
8	1179	36	196.50 +/- 5.75	6.00 +/- 3.35
9	1223	12	203.83 +/- 5.49	2.00 +/- 1.10
10	1181	21	196.83 +/- 3.92	3.50 +/- 1.05
11	1161	46	193.50 +/- 5.96	7.66 +/- 2.73
12	1194	39	199.00 +/- 1.55	6.50 +/- 2.17
13	1229	17	204.83 +/- 5.12	4.25 +/- 1.71
14	1168	35	194.67 +/- 4.68	5.83 +/- 1.94
15	1172	25	195.33 +/- 5.68	4.16 +/- 2.40
16	1228	38	204.66 +/- 4.93	6.33 +/- 2.25
17	1201	23	200.16 +/- 5.85	4.60 +/- 2.19
18	1203	34	200.50 +/- 3.45	5.67 +/- 2.88
19	1209	34	201.50 +/- 5.47	5.67 +/- 2.87
20	1220	27	203.33 +/- 6.47	4.50 +/- 2.43
21	1130	39	188.33 +/- 6.38	6.50 +/- 2.74
22	1140	71	190.00 +/- 10.30	11.83 +/- 4.18
23	1166	38	194.33 +/- 4.89	6.33 +/- 4.23
24	1171	39	195.17 +/- 5.00	6.50 +/- 3.27
25	1195	42	199.17 +/- 5.78	7.00 +/- 3.85
26	1178	75	196.33 +/- 3.50	12.50 +/- 4.18
27	1157	60	192.83 +/- 4.49	10.00 +/- 3.35
Avg	***1184.52 +/- 31.22***	***35.67 +/- 15.36***	***197.42 +/- 7.33***	***6.06 +/- 3.55***

*For the trial summary per block, we present mean +/- the standard deviation across blocks. The Avg row presents the mean ± standard deviation across participants. *Abbreviations:* Avg, average across participants.

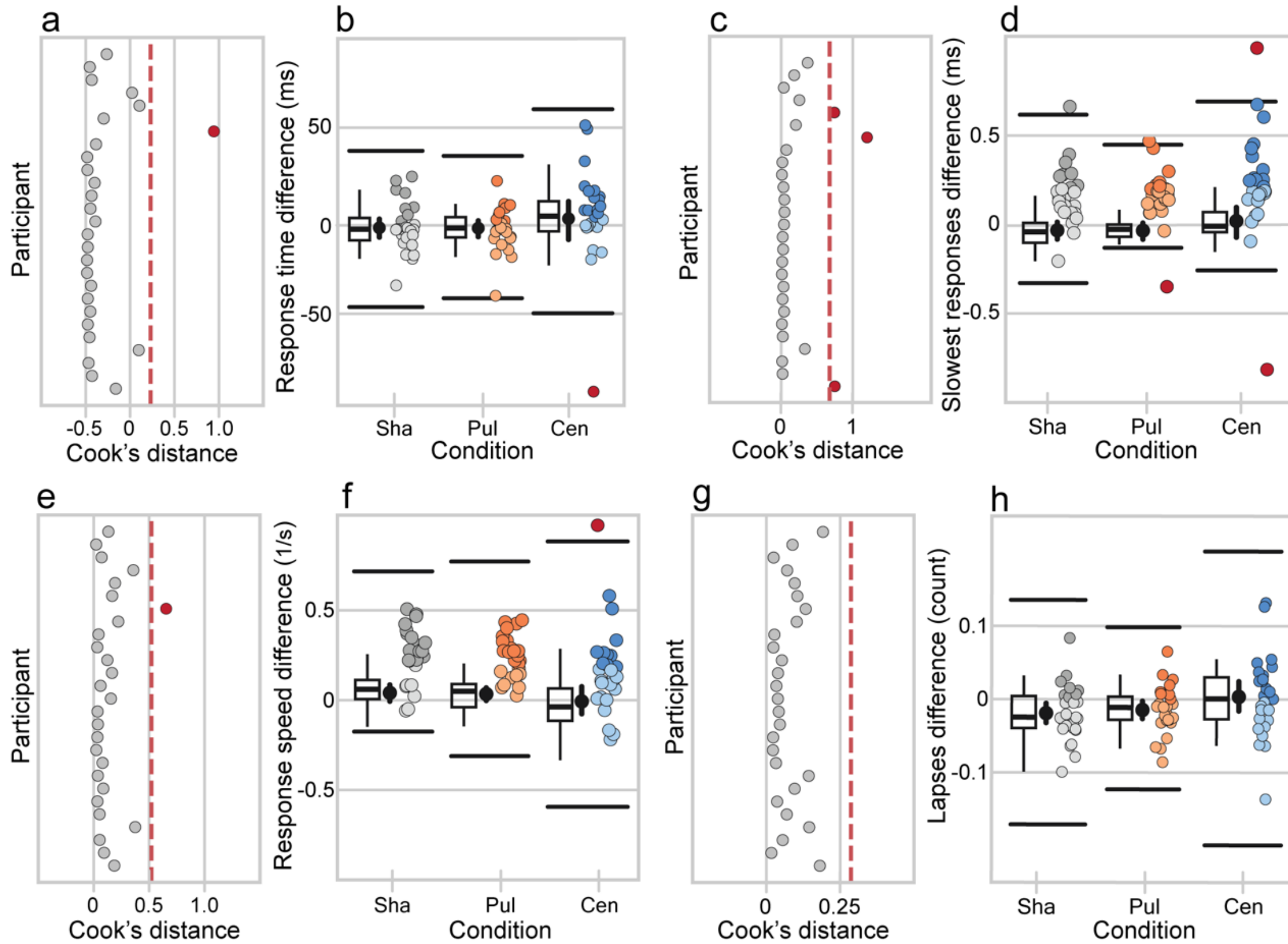

Supplementary Figure 1: **Data cleaning for the mixed-effects models examining performance during the Psychomotor Vigilance Task (PVT).** Boxes show first quartiles, medians, and third quartiles and whiskers span the datapoints within 1.5 × the IQR from the lower and upper hinges. Black points and bars next to each box show means and 95% confidence intervals across participants. **a, c, e, & g** Cook's distance for each participant in the mixed-effects models for response time (a), slowest 10% of responses (c), response speed (e), and lapses in attention (g). Red dashed line marks the exclusion threshold (4 x the mean across participants). Points in red are participants who surpassed the Cook's distance exclusion threshold and underwent further evaluation. **b, d, f, & h** Distribution of differences in response time (b), slowest 10% of responses (d), response speed (f), and lapses in attention (h) across blocks for each ultrasound condition. Individual points are participant means and are lighter or darker if the participant had greater value of the outcome before or after sonication, respectively. Solid black horizontal lines show outer fences based on Tukey's method (less than the first quartile minus 3 × the IQR or greater than the third quartile plus 3 × the IQR) for each condition. Participants in red are those who surpassed the Cook's distance exclusion threshold for the outcome's mixed-effects model. *Abbreviations:* Sha, sham condition; Pul, pulvinar condition; Cen, central thalamus condition.

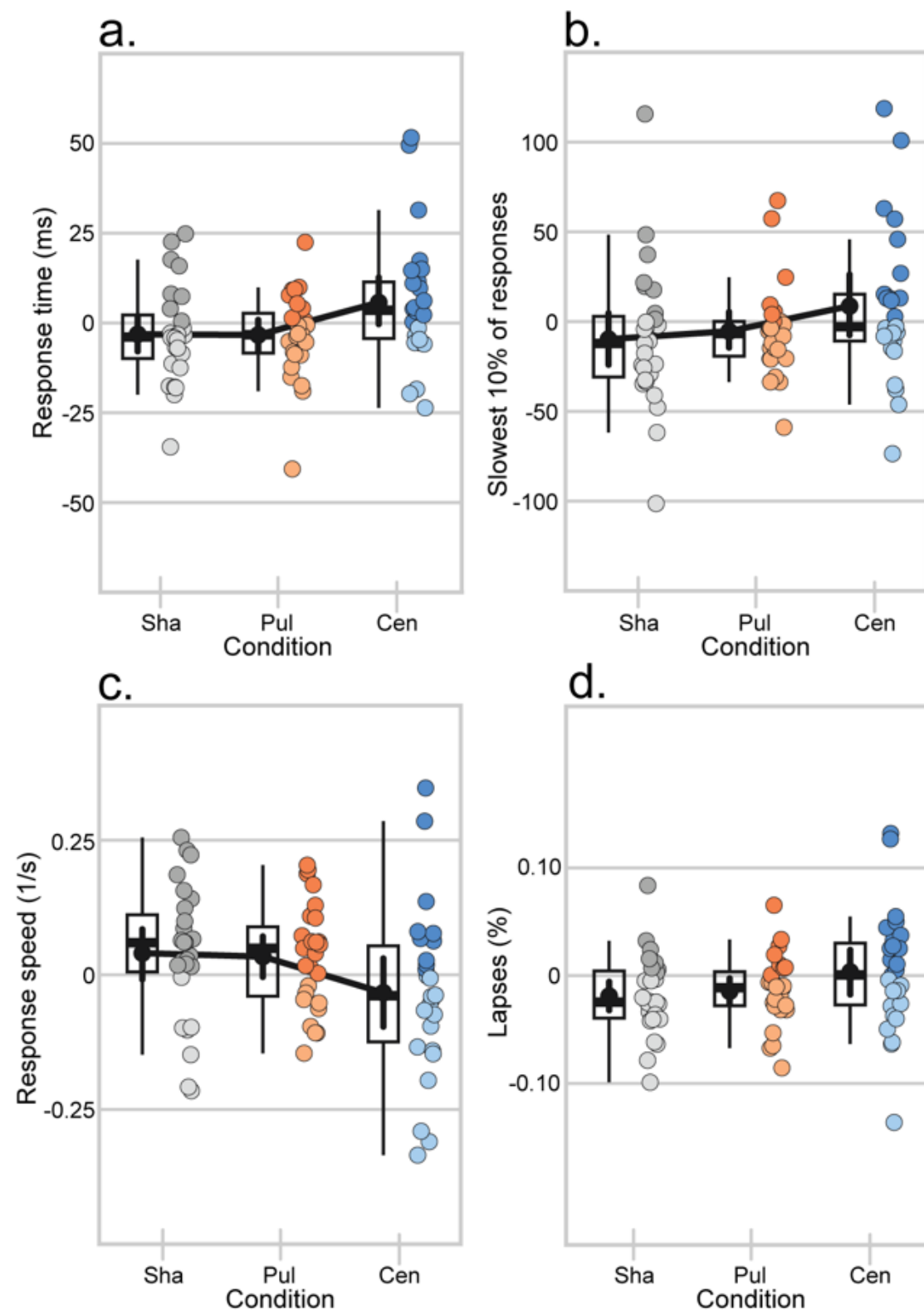

Supplementary Figure 2: **Additional descriptive statistics for the cleaned Psychomotor Vigilance Task (PVT) data used in the mixed-effects models.** Distribution of the changes in response times (a), the slowest 10% of responses (b), response speeds (c), and lapses in attention (d) across blocks (post- minus pre-sonication) in each ultrasound condition. Boxes show first quartiles, medians, and third quartiles and whiskers span the datapoints within 1.5 × the IQR from the lower and upper hinges. Black points and bars next to each box show means and 95% confidence intervals across participants. Individual points are participant means and are lighter or darker if the participant had greater value of the outcome before or after sonication, respectively. *Abbreviations:* Sha, sham condition; Pul, pulvinar condition; Cen, central thalamus condition.

Additional mixed-effects modeling results

Response time

There was no effect of biological sex or age on response times during the PVT, as indicated by the corresponding fixed effect terms ('F-M') and ('Age') in the model, respectively (see Supplementary Table 2). The random intercept for participant with the random slope for ultrasound condition effectively captured some individual differences in baseline (before sonication) response times between the sessions (and thus conditions), as indicated by the random slopes (see Supplementary Fig. 3b, right, and Supplementary Table 2). No meaningful variability in response time based on task order was observed, as indicated by the random effect for task order (see Supplementary Fig. 3c and Supplementary Table 2), which suggests that the order in which participants completed the tasks did not explain differences in response times. There were no differences in how response times changed over the duration of the task across blocks between the sham and pulvinar conditions (estimate = -0.48 ms, SE = 0.48, 95% CI = [-1.43, 0.46], t = -1.01, p = 0.31), the sham and central thalamus conditions (estimate = 0.30 ms, SE = 0.49, 95% CI = [-0.65, 1.25], t = 0.62, p = 0.54), or the pulvinar and central thalamus conditions (estimate = 0.78 ms, SE = 0.49, 95% CI = [-0.17, 1.74], z = 1.62, p = 0.11, p_{adj} = 0.16; see main text Fig. 2C).

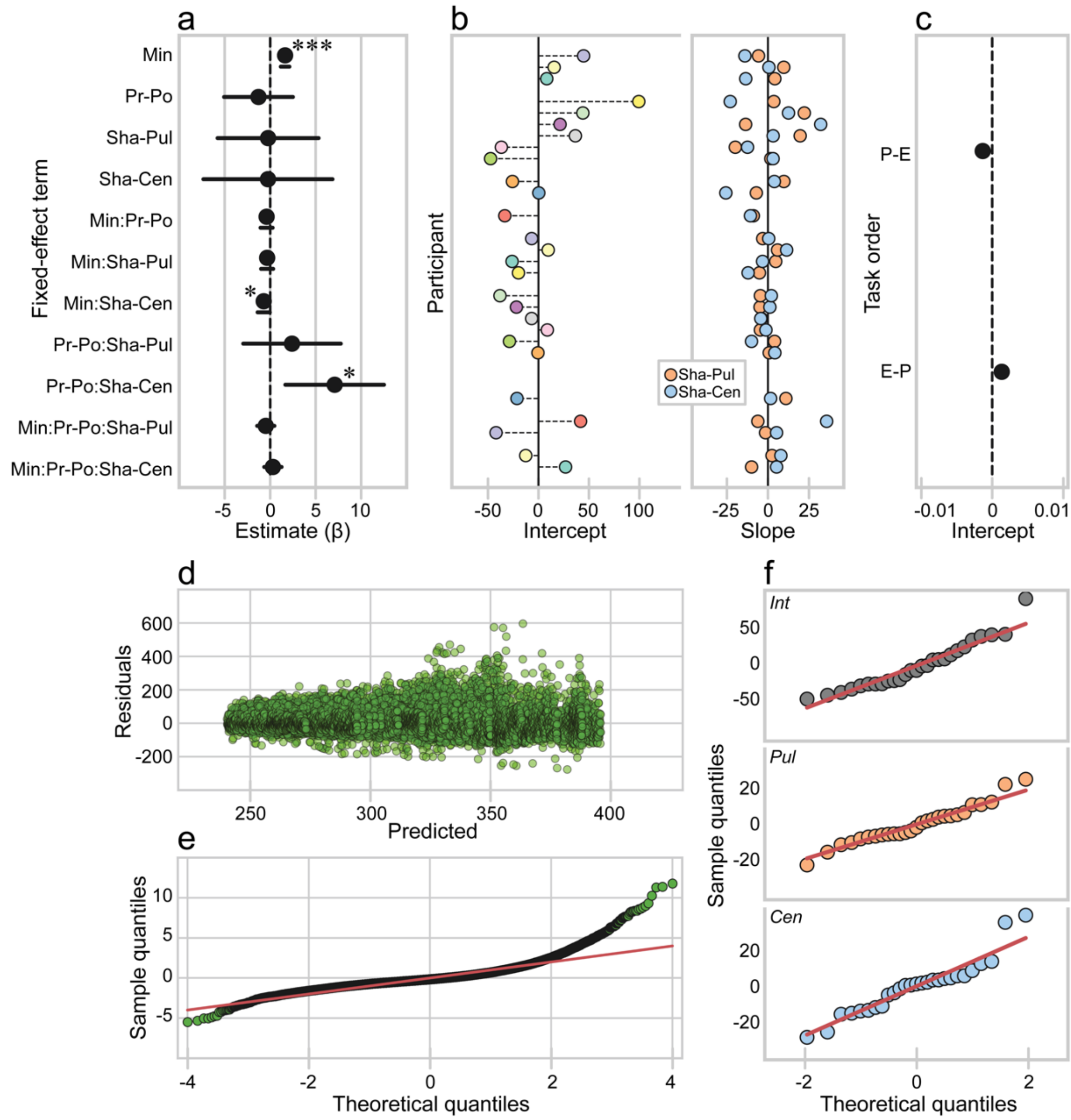

Supplementary Figure 3: **Summary of the linear mixed-effects model examining response times during the Psychomotor Vigilance Task (PVT). a** Fixed effect estimates. Bars show 95% confidence intervals. **b & c** Random effects. **b** Random intercept for participant (left) with a random slope for ultrasound condition (right). The random intercept for each participant shows their baseline response time (when block is at pre-sonication and ultrasound condition is at sham sonication) relative to the average baseline response time across participants in the fixed effects (0, black line). The random slope for ultrasound condition for each participant shows the difference in their baseline response time (when block is at pre-sonication) between the pulvinar and sham conditions (orange) and the central thalamus and sham conditions (blue) relative to the average across participants in the fixed effects (0, black line). **c** Random intercept for task order. Points show the average baseline response time (when block is at pre-sonication and ultrasound condition is at sham sonication) for each task order group, PVT then EDT ('P-E') and EDT then PVT ('E-P'). **d** Predicted values versus residuals. **e** QQ plot for theoretical quantiles versus residual (sample) quantiles. **f** QQ plot showing theoretical quantiles versus sample quantiles for the random intercept for participant (top, black) as well as the random slopes for the steps in condition from sham to pulvinar (middle, orange) and from sham to central thalamus (bottom, blue). *Abbreviations:* Int, intercept; Min, minute; Pr, pre-sonication; Po, post-sonication; Sha, sham condition; Pul, pulvinar condition; Cen, central thalamus condition.

Supplementary Table 2: Response time ~ minute × block × condition + sex + age + (1 + condition | participant) + (1 | task order)

Model summary

	REML	*Marg R^2*	*Cond R^2*	*ICC*			
	337848	0.01	0.33	0.33			
Fit (ANOVA)	*AIC*	*BIC*	*Log L*	*Deviance*	χ^2	*df*	*p*
Null/restricted	338074	338150	-169028	338056			
Full	337912	338080	-168936	337872	184.04	11	**<0.001**
Fixed effect	*Estimate*		*SE*	*95% CI*	*t*	*p*	
Int	301.14		45.17	212.60 – 389.68	6.67	**<0.001**	***
Min	1.64		0.24	1.17 – 2.11	6.81	**<0.001**	***
Pr-Po	-1.29		1.93	-5.07 – 2.50	-0.67	0.505	
Sha-Pul	-0.23		2.83	-5.78 – 5.32	-0.08	0.935	
Sha-Cen	-0.26		3.62	-7.35 – 6.84	-0.07	0.943	
F-M	-9.16		25.50	-59.14 – 40.82	-0.36	0.719	
Age	-0.27		2.05	-4.27 – 3.74	-0.13	0.897	
Min:Pr-Po	-0.39		0.34	-1.06 – 0.27	-1.16	0.247	
Min:Sha-Pul	-0.33		0.34	-1.00 – 0.33	-0.98	0.327	
Min:Sha-Cen	-0.71		0.34	-1.38 – -0.04	-2.07	**0.038**	*
Pr-Po:Sha-Pul	2.40		2.74	-2.96 – 7.76	0.88	0.380	
Pr-Po:Sha-Cen	7.09		2.76	1.68 – 12.51	2.57	**0.010**	*
Min:Pr-Po:Sha-Pul	-0.48		0.48	-1.43 – 0.46	-1.01	0.313	
Min:Pr-Po:Sha-Cen	0.30		0.49	-0.65 – 1.25	0.62	0.537	
Random effect	*N (obs)*		*Factor*	*Variance*	*Std*	*Corr*	
Participant	27 (31598)		Int	1083.17	32.91		
			Sha-Pul	114.90	10.72	0.21	
			Sha-Cen	241.00	15.52	0.02	0.10
Task order	2		Int	0.02	0.12		
Residual				2554.21	50.54		

*Linear mixed-effects model fit with REML. Units in milliseconds (ms). Grey rows indicate results reported in the main text. ":" denotes interaction terms, reflecting the additional change in the outcome across the stated levels of the fixed effects. Each block lasted 10 minutes, making 'Min' a continuous variable for minutes into the task block that captures change in the outcome per minute over the duration of a task block. Block is a categorical factor with two levels: including before sonication ('Pr') and after sonication ('Po'). Condition is a categorical factor with three levels: including sham ('Sha'), pulvinar ('Pul'), and central thalamic sonication ('Cen'). The reference levels for block and condition are pre-sonication ('Pr') and sham sonication ('Sha'), respectively. Random effects include a participant-level random intercept (baseline differences in the outcome across individuals) and a participant-level random slope for condition (individual differences in the effect of condition on the outcome at baseline, before sonication). *Abbreviations:* Marg, marginal; Cond, conditional; Log L, log-likelihood; CI, confidence interval; Int, intercept; Min, minute; Pr, pre-sonication block; Po, post-sonication block; Sha, sham; Pul, pulvinar; Cen, central thalamic; M, male; F, female; Corr, correlation. * p < 0.05, ** p < 0.01, *** p < 0.001.

Supplementary Table 3: Response time estimated marginal means

	Cond	Block	Mean	SE	CI (lower)	CI (upper)
**Avg**	Sha	Pr	298.50	12.59	273.82	323.18
	Sha	Po	295.28	12.59	270.60	319.96
	Pul	Pr	296.63	12.97	271.21	322.06
	Pul	Po	293.43	12.97	268.01	318.86
	Cen	Pr	294.75	12.98	269.30	320.19
	Cen	Po	300.09	12.98	274.64	325.53
**Min (0)**	Sha	Pr	290.43	12.65	265.64	315.22
	Sha	Po	289.15	12.65	264.36	313.93
	Pul	Pr	290.20	13.03	264.67	315.73
	Pul	Po	291.31	13.03	265.79	316.84
	Cen	Pr	290.18	13.04	264.62	315.73
	Cen	Po	295.98	13.04	270.42	321.53
**Min (slope)**	Sha	Pr	1.64	0.24	1.17	2.11
	Sha	Po	1.25	0.24	0.78	1.72
	Pul	Pr	1.31	0.24	0.84	1.78
	Pul	Po	0.43	0.24	-0.04	0.90
	Cen	Pr	0.93	0.24	0.45	1.41
	Cen	Po	0.84	0.25	0.35	1.32

*Units in milliseconds (ms). Model-based estimates of the mean outcome for each level, averaging over and controlling for the other variables in the model. Avg represents average across all other factors. Min (slope) represents the average change in the outcome variable per minute into the task. Min (0) represents task onset (0 minutes into the task block). *Abbreviations:* Cond, condition; Sha, sham sonication; Pul, pulvinar sonication; Cen, central thalamic sonication; Pr, pre-sonication; Po, post-sonication; Min, minute into the task block.

Supplementary Table 4: Response time estimated marginal means contrasts

Contrast	Est	SE	CI (lower)	CI (upper)	z	p	p_{adj}	
Pul-Sha | Po-Pr	0.02	1.38	-2.69	2.73	0.01	0.989	0.989	
Cen-Sha | Po-Pr	8.56	1.40	5.82	11.30	6.13	**<0.001**	**<0.001**	***
Cen-Pul | Po-Pr	8.55	1.40	5.80	11.29	6.11	**<0.001**	**<0.001**	***
Min (0) | Cen-Pul | Po-Pr	4.69	2.77	-0.73	10.11	1.70	**0.090**	0.127	
Min (slope) | Pr	1.29	0.14	1.02	1.57	9.27	**<0.001**	**<0.001**	***
Min (slope) | Cen-Pul | Po-Pr	0.78	0.49	-0.17	1.74	1.62	0.106	0.127	

*Units in milliseconds (ms). Grey rows mark results presented in the main manuscript. Min (slope) represents the average change in the outcome variable per minute into the task. Min (0) represents task onset (0 minutes into the task block). *Abbreviations:* Est, estimate; CI, confidence interval; Min, minute; Pr, pre-sonication; Po, post-sonication; Sha, sham sonication; Pul, pulvinar sonication; Cen, central thalamic sonication; p_{adj}, p-value after correcting for multiple comparisons using the Benjamini-Hochberg method. † $p < 0.10$ before and/or after correction, * $p < 0.05$, ** $p < 0.01$, *** $p < 0.001$ after correction.

Slowest 10% of responses

There was no effect of biological sex or age on the slowest 10% of responses, as indicated by the corresponding fixed effect terms (‘F-M’) and (‘Age’) in the model, respectively (see Supplementary Table 5). The random intercept for participant with the random slope for ultrasound condition effectively captured individual differences in baseline (pre-sonication) values of the slowest 10% of responses between the sessions (and thus conditions), as indicated by the random slopes for ultrasound condition per participant (see Supplementary Fig. 4b, right, and Supplementary Table 5). Slight variability in the slowest 10% of responses based on task order was captured by the random effect for task order (see Supplementary Fig. 4c and Supplementary Table 5). No time-on-task effect was observed for the slowest 10% of responses, as the slowest 10% of responses showed no consistent change per minute into the task (-0.55 ms, SE = 0.34, 95% CI = [-1.22, 0.12], $z = -1.61$, $p = 0.11$, $p_{adj} = 0.25$; see main text Fig. 2F), averaging over the ultrasound conditions.

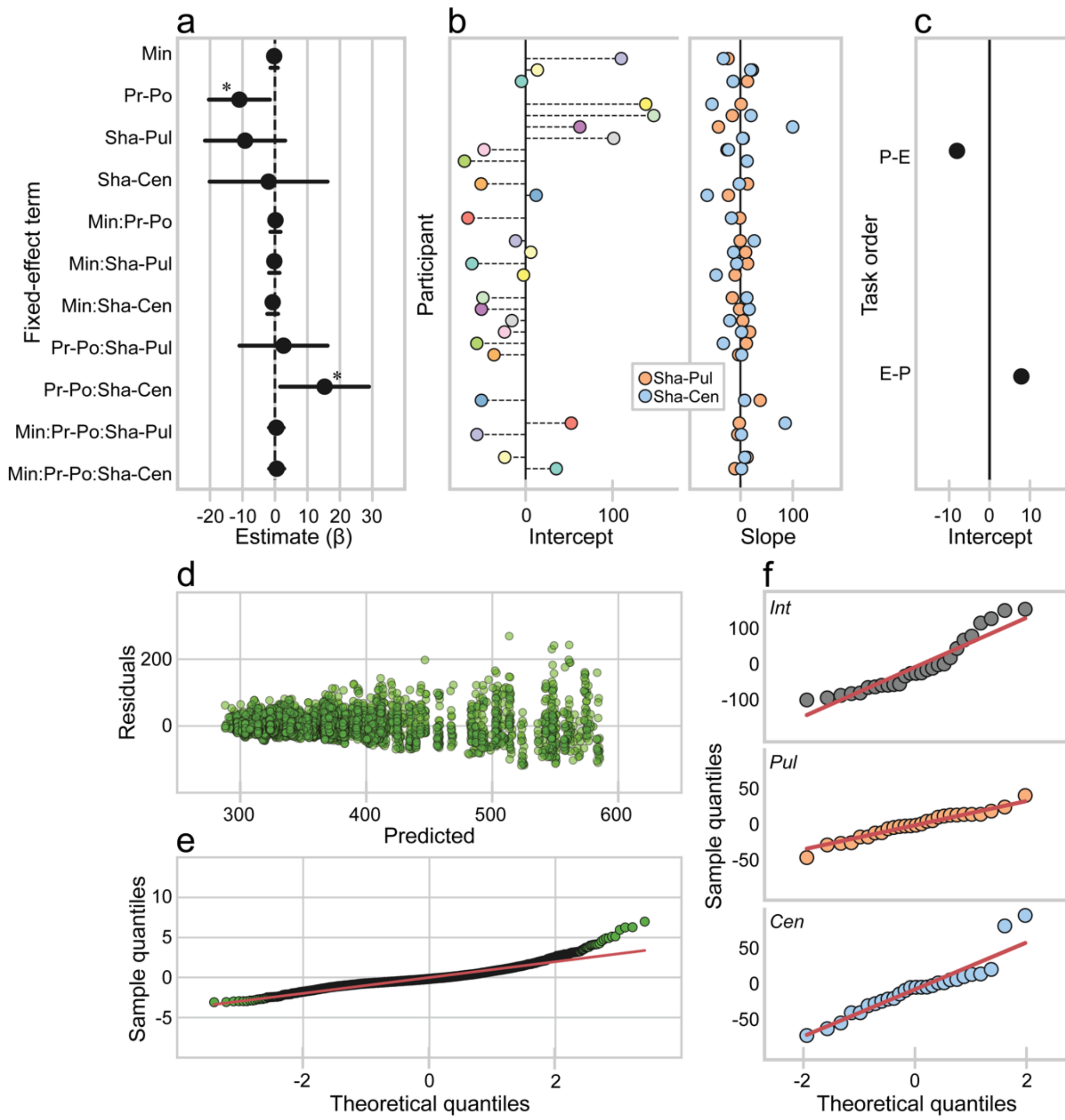

Supplementary Figure 4: **Summary of the linear mixed-effects model examining slowest 10% of responses during the Psychomotor Vigilance Task (PVT). a** Fixed effect estimates. Bars show 95% confidence intervals. **b & c** Random effects. **b** Random intercept for participant (left) with a random slope for ultrasound condition (right). The random intercept for each participant shows their baseline slowest 10% of responses (when block is at pre-sonication and ultrasound condition is at sham sonication) relative to the average baseline slowest 10% of responses across participants in the fixed effects (0, black line). The random slope for ultrasound condition for each participant shows the difference in their baseline slowest 10% of responses (when block is at pre-sonication) between the pulvinar and sham conditions (orange) and the central thalamus and sham conditions (blue) relative to the average across participants in the fixed effects (0, black line). **c** Random intercept for task order. Points show the average baseline slowest 10% of responses (when block is at pre-sonication and ultrasound condition is at sham sonication) for each task order group, PVT then EDT ('P-E') and EDT then PVT ('E-P'). **d** Predicted values versus residuals. **e** QQ plot for theoretical quantiles versus residual (sample) quantiles. **f** QQ plot showing theoretical quantiles versus sample quantiles for the random intercept for participant (top, black) as well as the random slopes for the steps in condition from sham to pulvinar (middle, orange) and from sham to central thalamus (bottom, blue). *Abbreviations:* Int, intercept; Min, minute; Pr, pre-sonication; Po, post-sonication; Sha, sham condition; Pul, pulvinar condition; Cen, central thalamus condition.

Supplementary Table 5: Slowest 10% of responses ~ minute × block × condition + sex + age + (1 + condition | participant) + (1 | task order)

Model summary

	REML	*Marg R^2*	*Cond R^2*	*ICC*			
	32205	0.046	0.834	0.83			
Fit (ANOVA)	*AIC*	*BIC*	*Log L*	*Deviance*	*χ^2*	*df*	*p*
Null/restricted	32313	32367	-16148	32295			
Full	32294	32415	-16127	32254	41.11	11	**<0.001**
Fixed effect	*Estimate (ms)*		*SE*	*95% CI*	*t*	*p*	
Int	355.92		98.06	163.65 – 548.18	3.63	**<0.001**	***
Min	-0.25		0.57	-1.37 – 0.87	-0.44	0.662	
Pr-Po	-10.93		4.79	-20.33 – -1.54	-2.28	**0.023**	*
Sha-Pul	-9.37		6.31	-21.74 – 3.01	-1.48	0.138	
Sha-Cen	-1.74		9.26	-19.90 – 16.41	-0.19	0.851	
F-M	75.79		55.32	-32.67 – 184.25	1.37	0.171	
Age	-1.22		4.42	-9.88 – 7.45	-0.28	0.783	
Min:Pr-Po	0.16		0.80	-1.41 – 1.72	0.20	0.843	
Min:Sha-Pul	-0.20		0.82	-1.81 – 1.41	-0.25	0.804	
Min:Sha-Cen	-0.70		0.84	-2.35 – 0.95	-0.83	0.405	
Pr-Po:Sha-Pul	2.64		6.91	-10.92 –16.19	0.38	0.703	
Pr-Po:Sha-Cen	15.28		6.97	1.61 – 28.96	2.19	**0.029**	*
Min:Pr-Po:Sha-Pul	0.41		1.15	-1.84 – 2.67	0.36	0.721	
Min:Pr-Po:Sha-Cen	0.53		1.18	-1.77 – 2.84	0.45	0.651	
Random effect	*N (obs)*		*Factor*	*Variance*	*Std*	*Corr*	
Participant	27 (3153)		Int	6346.8	79.67		
			Sha-Pul	390.8	19.77	-0.51	
			Sha-Cen	1521.9	39.01	0.14	-0.05
Task order	2		Int	336.3	18.34		
Residual				1484.6	38.53		

*Linear mixed-effects model fit with REML. Units in milliseconds (ms). Grey rows indicate results reported in the main text. ":" denotes interaction terms, reflecting the additional change in the outcome across the stated levels of the fixed effects. Each block lasted 10 minutes, making 'Min' a continuous variable for minutes into the task block that captures change in the outcome per minute over the duration of a task block. Block is a categorical factor with two levels: including before sonication ('Pr') and after sonication ('Po'). Condition is a categorical factor with three levels: including sham ('Sha'), pulvinar ('Pul'), and central thalamic sonication ('Cen'). The reference levels for block and condition are pre-sonication ('Pr') and sham sonication ('Sha'), respectively. Random effects include a participant-level random intercept (baseline differences in the outcome across individuals) and a participant-level random slope for condition (individual differences in the effect of condition on the outcome at baseline, before sonication). *Abbreviations:* Marg, marginal; Cond, conditional; Log L, log-likelihood; CI, confidence interval; Int, intercept; Min, minute; Pr, pre-sonication block; Po, post-sonication block; Sha, sham; Pul, pulvinar; Cen, central thalamic; M, male; F, female; Corr, correlation. † $p < 0.10$, * $p < 0.05$, ** $p < 0.01$, *** $p < 0.001$.

Supplementary Table 6: Slowest 10% of responses estimated marginal means

	Cond	Block	Mean	SE	CI (lower)	CI (upper)
**Avg**	Sha	Pr	364.58	31.07	303.69	425.47
	Sha	Po	354.47	31.07	293.58	415.36
	Pul	Pr	354.15	30.36	294.64	413.65
	Pul	Po	348.82	30.36	289.32	408.33
	Cen	Pr	359.18	32.54	295.41	422.94
	Cen	Po	367.13	32.54	303.36	430.91
**Min (0)**	Sha	Pr	365.88	31.22	304.70	427.06
	Sha	Po	354.95	31.20	293.80	416.10
	Pul	Pr	356.51	30.52	296.69	416.34
	Pul	Po	348.22	30.50	288.44	408.00
	Cen	Pr	364.14	32.69	300.07	428.21
	Cen	Po	368.49	32.69	304.42	432.55
**Min (slope)**	Sha	Pr	-0.25	0.57	-1.37	0.87
	Sha	Po	-0.09	0.56	-1.19	1.00
	Pul	Pr	-0.45	0.59	-1.61	0.70
	Pul	Po	0.12	0.58	-1.03	1.26
	Cen	Pr	-0.95	0.62	-2.16	0.26
	Cen	Po	-0.26	0.61	-1.45	0.93

*Units in milliseconds (ms). Model-based estimates of the mean outcome for each level, averaging over and controlling for the other variables in the model. Avg represents average across all other factors. Min (slope) represents the average change in the outcome variable per minute into the task. Min (0) represents task onset (0 minutes into the task block). *Abbreviations:* Cond, condition; Sha, sham sonication; Pul, pulvinar sonication; Cen, central thalamic sonication; Pr, pre-sonication; Po, post-sonication; Min, minute into the task block.

Supplementary Table 7: Slowest 10% of responses estimated marginal means contrasts

Contrast	Est	SE	CI (lower)	CI (upper)	z	p	p_{adj}	
Pul-Sha | Po-Pr	4.79	3.33	-1.74	11.31	1.44	0.150	0.180	
Cen-Sha | Po-Pr	18.07	3.37	11.47	24.67	5.36	**<0.001**	**<0.001**	***
Cen-Pul | Po-Pr	13.28	3.40	6.61	19.95	3.90	**<0.001**	**<0.001**	***
Min (0) | Cen-Pul | Po-Pr	12.64	7.11	-1.29	26.58	1.78	**0.075**	0.150	†
Min (slope) | Pr	-0.55	0.34	-1.22	0.12	-1.61	0.108	0.162	
Min (slope) | Cen-Pul | Po-Pr	0.12	1.20	-2.22	2.47	0.10	0.919	0.919	

*Units in milliseconds (ms). Grey rows mark results presented in the main manuscript. Min (slope) represents the average change in the outcome variable per minute into the task. Min (0) represents task onset (0 minutes into the task block). *Abbreviations:* Est, estimate; CI, confidence interval; Min, minute; Pr, pre-sonication; Po, post-sonication; Sha, sham sonication; Pul, pulvinar sonication; Cen, central thalamic sonication; p_{adj}, p-value after correcting for multiple comparisons using the Benjamini-Hochberg method. † $p < 0.10$ before and/or after correction. * $p < 0.05$, ** $p < 0.01$, *** $p < 0.001$ after correction.

Response speed

There was no effect of biological sex or age on response speed, as indicated by the corresponding fixed effect terms (‘F-M’) and (‘Age’) in the model, respectively (see Supplementary Table 8). The random intercept for participant with the random slope for ultrasound condition effectively captured some individual differences in baseline (before sonication) response speeds between the sessions (and thus conditions), as indicated by the random slopes for ultrasound condition (see Supplementary Fig. 5b, right, and Supplementary Table 8). No meaningful variability in response speed based on task order was captured by the model, as indicated by the random effect for task order (see Supplementary Fig. 5c and Supplementary Table 8), which indicates that the order in which participants completed the tasks did not explain differences in response speeds. There were no differences in how response speeds changed over the duration of the task across blocks between the sham and pulvinar conditions (0.00 1/s, SE = 0.00, 95% CI = [-0.01, 0.01], t = 0.24, p = 0.81), the sham and central thalamus conditions (0.00 1/s, SE = 0.00, 95% CI = [-0.01, 0.01], t = -0.67, p = 0.50), or the pulvinar and central thalamus conditions (0.00 1/s, SE = 0.00, 95% CI = [-0.01, 0.01], z = -0.91, p = 0.36, p_{adj} = 0.43; see main text Fig. 2I).

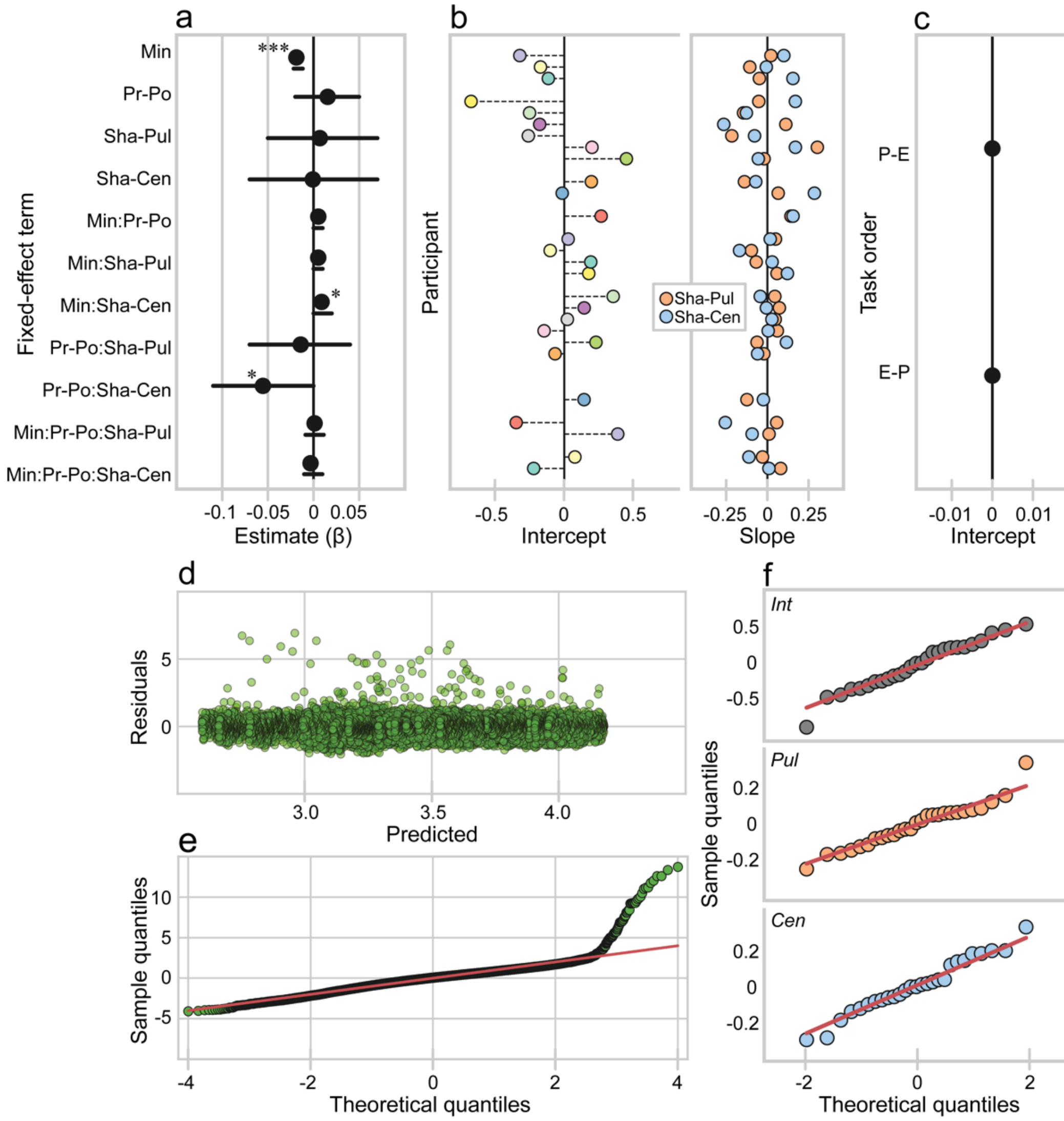

Supplementary Figure 5: **Summary of the linear mixed-effects model examining response speeds during the Psychomotor Vigilance Task (PVT). a** Fixed effect estimates. Bars show 95% confidence intervals. **b & c** Random effects. **b** Random intercept for participant (left) with a random slope for ultrasound condition (right). The random intercept for each participant shows their baseline response speed (when block is at pre-sonication and ultrasound condition is at sham sonication) relative to the average baseline response speed across participants in the fixed effects (0, black line). The random slope for ultrasound condition for each participant shows the difference in their baseline response speed (when block is at pre-sonication) between the pulvinar and sham conditions (orange) and the central thalamus and sham conditions (blue) relative to the average across participants in the fixed effects (0, black line). **c** Random intercept for task order. Points show the average baseline response speed (when block is at pre-sonication and ultrasound condition is at sham sonication) for each task order group, PVT then EDT ('P-E') and EDT then PVT ('E-P'). **d** Predicted values versus residuals. **e** QQ plot for theoretical quantiles versus residual (sample) quantiles. **f** QQ plot showing theoretical quantiles versus sample quantiles for the random intercept for participant (top, black) as well as the random slopes for the steps in condition from sham to pulvinar (middle, orange) and from sham to central thalamus (bottom, blue). *Abbreviations:* Int, intercept; Min, minute; Pr, pre-sonication; Po, post-sonication; Sha, sham condition; Pul, pulvinar condition; Cen, central thalamus condition.

Supplementary Table 8: Response speed ~ minute × block × condition + sex + age + (1 + condition | participant) + (1 | task order)

**Model summary**							
	REML	*Marg R^2*	*Cond R^2*	*ICC*			
	46864	0.01	0.35	0.34			
Fit (ANOVA)	*AIC*	*BIC*	*Log L*	*Deviance*	χ^2	*df*	*p*
Null/restricted	46979	47054	-23480	46961			
Full	46799	46966	-23380	46759	201.48	11	**<0.001**
Fixed effect	*Estimate (1/s)*		*SE*	*95% CI*	*t*	*p*	
Int	3.50		0.45	2.61 – 4.38	7.75	**<0.001**	***
Min	-0.02		0.00	-0.02 – -0.01	-7.79	**<0.001**	***
Pr-Po	0.02		0.02	-0.02 – 0.05	0.80	0.421	
Sha-Pul	0.01		0.03	-0.05 – 0.07	0.23	0.820	
Sha-Cen	-0.00		0.04	-0.07 – 0.07	-0.02	0.984	
F-M	0.18		0.25	-0.32 – 0.68	0.69	0.491	
Age	-0.00		0.02	-0.04 – 0.04	-0.13	0.895	
Min:Pr-Po	0.01		0.00	-0.00 – 0.01	1.54	0.124	
Min:Sha-Pul	0.01		0.00	-0.00 – 0.01	1.52	0.128	
Min:Sha-Cen	0.01		0.00	0.00 – 0.02	2.57	**0.010**	*
Pr-Po:Sha-Pul	-0.01		0.03	-0.07 – 0.04	-0.52	0.606	
Pr-Po:Sha-Cen	-0.06		0.03	-0.11 – -0.00	-2.01	**0.045**	*
Min:Pr-Po:Sha-Pul	0.00		0.00	-0.01 – 0.01	0.24	0.809	
Min:Pr-Po:Sha-Cen	-0.00		0.00	-0.01 – 0.01	-0.67	0.502	
Random effect	*N (obs)*		*Factor*	*Variance*	*Std*	*Corr*	
Participant	27 (31598)		Int	0.11	0.33		
			Sha-Pul	0.02	0.12	0.25	
			Sha-Cen	0.02	0.15	0.05	0.29
Task order	2		Int	0.00	0.00		
Residual				0.03	0.51		

*Linear mixed-effects model fit with REML. Units in 1/s (1/RT). Grey rows indicate results reported in the main text. ":" denotes interaction terms, reflecting the additional change in the outcome across the stated levels of the fixed effects. Each block lasted 10 minutes, making 'Min' a continuous variable for minutes into the task block that captures change in the outcome per minute over the duration of a task block. Block is a categorical factor with two levels: including before sonication ('Pr') and after sonication ('Po'). Condition is a categorical factor with three levels: including sham ('Sha'), pulvinar ('Pul'), and central thalamic sonication ('Cen'). The reference levels for block and condition are pre-sonication ('Pr') and sham sonication ('Sha'), respectively. Random effects include a participant-level random intercept (baseline differences in the outcome across individuals) and a participant-level random slope for condition (individual differences in the effect of condition on the outcome at baseline, before sonication). *Abbreviations:* Marg, marginal; Cond, conditional; Log L, log-likelihood; CI, confidence interval; Int, intercept; Min, minute; Pr, pre-sonication block; Po, post-sonication block; Sha, sham; Pul, pulvinar; Cen, central thalamic; M, male; F, female; Corr, correlation. † p < 0.10, * p < 0.05, ** p < 0.01, *** p < 0.001.

Supplementary Table 9: Response speed estimated marginal means

	Cond	Block	Mean	SE	CI (lower)	CI (upper)
**Avg**	Sha	Pr	3.43	0.13	3.18	3.68
	Sha	Po	3.47	0.13	3.23	3.72
	Pul	Pr	3.46	0.13	3.21	3.72
	Pul	Po	3.50	0.13	3.24	3.75
	Cen	Pr	3.47	0.13	3.22	3.73
	Cen	Po	3.44	0.13	3.19	3.70
**Min (0)**	Sha	Pr	3.52	0.13	3.28	3.77
	Sha	Po	3.54	0.13	3.29	3.79
	Pul	Pr	3.53	0.13	3.27	3.79
	Pul	Po	3.53	0.13	3.27	3.79
	Cen	Pr	3.52	0.13	3.27	3.78
	Cen	Po	3.48	0.13	3.23	3.74
**Min (slope)**	Sha	Pr	-0.02	0.00	-0.02	-0.01
	Sha	Po	-0.01	0.00	-0.02	-0.01
	Pul	Pr	-0.01	0.00	-0.02	-0.01
	Pul	Po	-0.01	0.00	-0.01	0.00
	Cen	Pr	-0.01	0.00	-0.01	-0.01
	Cen	Po	-0.01	0.00	-0.01	0.00

*Units in 1/s (1/RT). Model-based estimates of the mean outcome for each level, averaging over and controlling for the other variables in the model. Avg represents average across all other factors. Min (slope) represents the average change in the outcome variable per minute into the task. Min (0) represents task onset (0 minutes into the task block). *Abbreviations:* Cond, condition; Sha, sham sonication; Pul, pulvinar sonication; Cen, central thalamic sonication; Pr, pre-sonication; Po, post-sonication; Min, minute into the task block.

Supplementary Table 10: Response speed estimated marginal means contrasts

Contrast	Est	SE	CI (lower)	CI (upper)	z	p	p_{adj}	
Pul-Sha | Po-Pr	-0.01	0.01	-0.04	0.02	-0.61	0.544	0.544	
Cen-Sha | Po-Pr	-0.07	0.01	-0.10	-0.04	-5.12	**<0.001**	**<0.001**	***
Cen-Pul | Po-Pr	-0.06	0.01	-0.09	-0.04	-4.51	**<0.001**	**<0.001**	***
Min (0) | Cen-Pul | Po-Pr	-0.04	0.03	-0.10	0.01	-1.50	0.135	0.202	
Min (slope) | Pr	-0.01	0.00	-0.02	-0.01	-10.12	**<0.001**	**<0.001**	***
Min (slope) | Cen-Pul | Po-Pr	0.00	0.00	-0.01	0.01	-0.91	0.362	0.434	

*Units in 1/s (1/RT). Grey rows mark results presented in the main manuscript. Min (slope) represents the average change in the outcome variable per minute into the task. Min (0) represents task onset (0 minutes into the task block). *Abbreviations:* Est, estimate; CI, confidence interval; Min, minute; Pr, pre-sonication; Po, post-sonication; Sha, sham sonication; Pul, pulvinar sonication; Cen, central thalamic sonication; p_{adj}, p-value after correcting for multiple comparisons using the Benjamini-Hochberg method. † $p < 0.10$ before and/or after correction. * $p < 0.05$, ** $p < 0.01$, *** $p < 0.001$ after correction.

Lapses in attention

There was no effect of biological sex or age on the odds of lapsing, as indicated by the fixed effect terms (‘F-M’) and (‘Age’) in the model, respectively (see Supplementary Table 11). The random intercept for participant with the random slope for ultrasound condition captured some individual differences in baseline (pre-sonication) odds of lapsing between the sessions (and thus the ultrasound conditions), as indicated by the random slopes for ultrasound condition for each participant (see Supplementary Fig. 7b, right, and Supplementary Table 11). Slight variability in the odds of lapsing based on task order was captured by the random effect for task order (see Supplementary Fig. 7c and Supplementary Table 11). There were no differences in how the odds of lapsing changed over the duration of the task across blocks between the sham and pulvinar conditions (odds ratio = 1.05, 95% CI = [0.95, 1.15], z = 0.97, p = 0.33), the sham and central thalamus conditions (odds ratio = 1.01, 95% CI = [0.93, 1.10], z = 0.25, p = 0.80), or the pulvinar and central thalamus conditions (odds ratio = 0.97, 95% CI = [0.88, 1.06], z = -0.75, p = 0.45, p_{adj} = 0.54; see main text Fig. 2L).

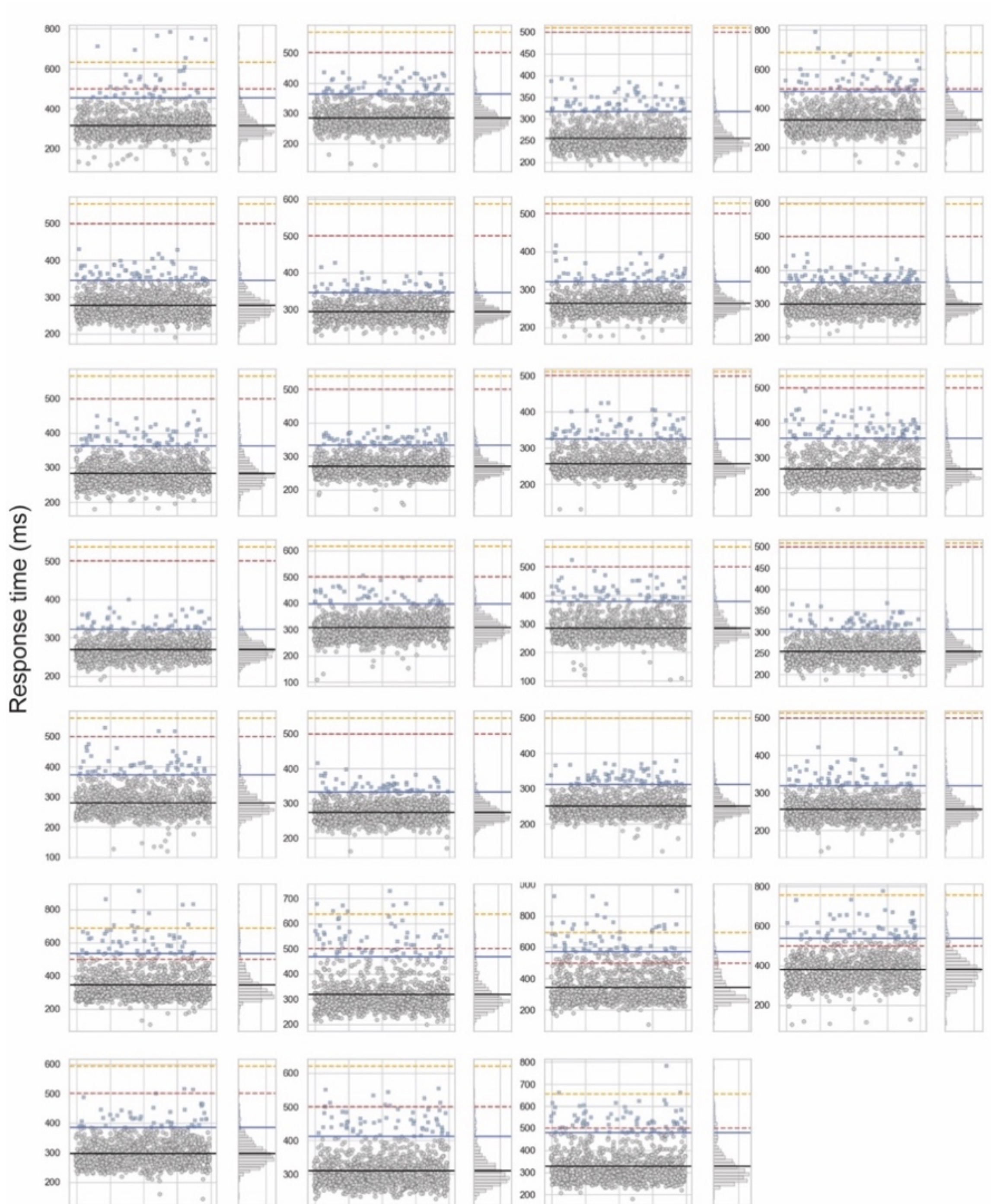

Supplementary Figure 6: **Defining lapses in attention during the Psychomotor Vigilance Task (PVT).** Each subplot shows the distribution of individual response times (grey points) and lapses in attention (blue '+' symbols) for each participant, combining the data from every PVT block. The solid blue lines mark the lapse in attention criterion we defined for the participants (their mean response time plus two standard deviations). The solid black line shows the mean response time for the participant, averaging over all PVT trials. The red and yellow dashed lines show the conventional lapse cutoffs of 500 ms and twice the mean response time, which our participants never or rarely surpassed.

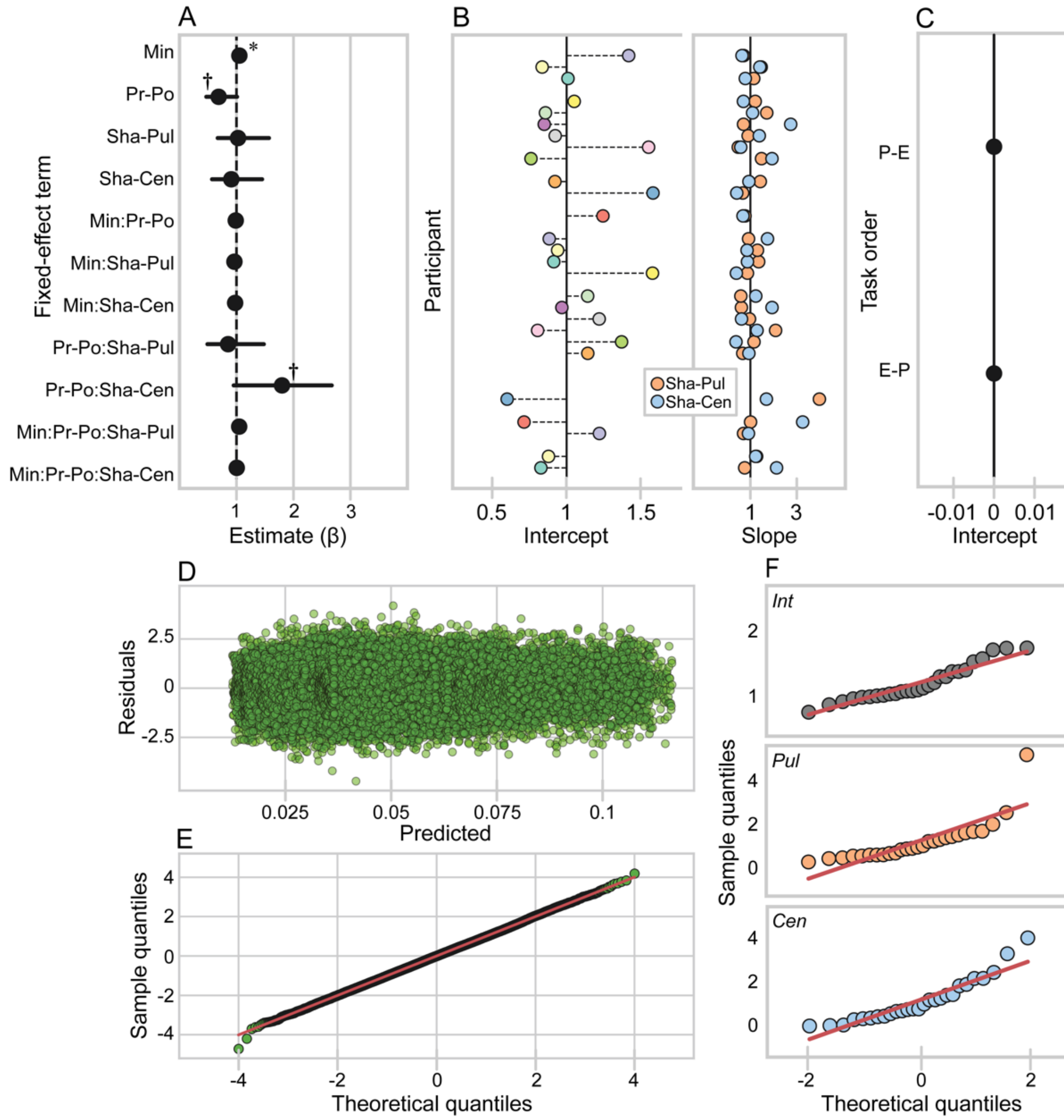

Supplementary Figure 7: **Summary of the mixed-effects logistic regression examining lapses in attention during the Psychomotor Vigilance Task (PVT). a** Fixed effect estimates. Bars show 95% confidence intervals. **b & c** Random effects. **b** Random intercept for participant (left) with a random slope for ultrasound condition (right). The random intercept for each participant shows their baseline odds of lapsing (when block is at pre-sonication and ultrasound condition is at sham sonication) relative to the average baseline odds of lapsing across participants in the fixed effects (0, black line). The random slope for ultrasound condition for each participant shows the difference in their baseline odds of lapsing (when block is at pre-sonication) between the pulvinar and sham conditions (orange) and the central thalamus and sham conditions (blue) relative to the average across participants in the fixed effects (0, black line). **c** Random intercept for task order. Points show the average baseline odds of lapsing (when block is at pre-sonication and ultrasound condition is at sham sonication) for each task order group, PVT then EDT ('P-E') and EDT then PVT ('E-P'). **d** Predicted values versus residuals. **e** QQ plot for theoretical quantiles versus residual (sample) quantiles. **f** QQ plot showing theoretical quantiles versus sample quantiles for the random intercept for participant (top, black) as well as the random slopes for the steps in condition from sham to pulvinar (middle, orange) and from sham to central thalamus (bottom, blue). *Abbreviations*: Int, intercept; Min, minute; Pr, pre-sonication; Po, post-sonication; Sha, sham condition; Pul, pulvinar condition; Cen, central thalamus condition.

Supplementary Table 11: Lapses ~ minute × block × condition + sex + age + (1 + condition | participant) + (1 | task order)

**Model summary**							
	Marg R^2	*Cond R^2*	*ICC*				
	0.02	0.07	0.06				
Fit (ANOVA)	*AIC*	*BIC*	*Log L*	*Deviance*	*χ^2*	*df*	*p*
Null/restricted	11875	11942	-5929.4	11859			
Full	11850	12026	-5904.0	11808	50.68	13	**<0.001**
Fixed effect	*Estimate (odds ratio)*		*95% CI*		*z*	*p*	
Int	0.04		0.02 – 0.07		-11.22	**<0.001**	***
Min	1.05		1.01 – 1.09		2.34	**0.019**	*
Pr-Po	0.69		0.47 – 1.01		-1.93	0.054	†
Sha-Pul	1.03		0.67 – 1.58		0.13	0.897	
Sha-Cen	1.02		0.65 – 1.62		0.09	0.926	
F-M	1.03		0.78 – 1.37		0.23	0.821	
Age	1.00		0.98 – 1.02		0.34	0.737	
Min:Pr-Po	0.99		0.93 – 1.05		-0.41	0.682	
Min:Sha-Pul	0.96		0.91 – 1.02		-1.23	0.218	
Min:Sha-Cen	0.97		0.91 – 1.03		-1.12	0.263	
Pr-Po:Sha-Pul	0.85		0.49 – 1.48		-0.57	0.570	
Pr-Po:Sha-Cen	1.55		0.93 – 2.60		1.68	0.094	†
Min:Pr-Po:Sha-Pul	1.05		0.95 – 1.15		0.97	0.333	
Min:Pr-Po:Sha-Cen	1.01		0.93 – 1.10		0.25	0.800	
Random effect	*N (obs)*		*Factor*	*Variance*	*Std*	*Corr*	
Participant	27 (31982)		Int	1.15	1.46		
			Sha-Pul	1.47	1.86	-0.72	
			Sha-Cen	1.84	2.18	-0.81	0.23
Task order	2		Int	1.00	1.00		

*Mixed-effects logistic regression. Units in odds ratios. An odds ratio of 1 reflects equal likelihood of lapsing between the compared groups. Grey rows indicate results reported in the main text. ":" denotes interaction terms, reflecting the additional change in the outcome across the stated levels of the fixed effects. Each block lasted 10 minutes, making 'Min' a continuous variable for minutes into the task block that captures change in the outcome per minute over the duration of a task block. Block is a categorical factor with two levels: including before sonication ('Pr') and after sonication ('Po'). Condition is a categorical factor with three levels: including sham ('Sha'), pulvinar ('Pul'), and central thalamic sonication ('Cen'). The reference levels for block and condition are pre-sonication ('Pr') and sham sonication ('Sha'), respectively. Random effects include a participant-level random intercept (baseline differences in the outcome across individuals) and a participant-level random slope for condition (individual differences in the effect of condition on the outcome at baseline, before sonication). *Abbreviations:* Marg, marginal; Cond, conditional; Log L, log-likelihood; CI, confidence interval; Int, intercept; Min, minute; Pr, pre-sonication block; Po, post-sonication block; Sha, sham; Pul, pulvinar; Cen, central thalamic; M, male; F, female; Corr, correlation. † $p < 0.10$, * $p < 0.05$, ** $p < 0.01$, *** $p < 0.001$.

Supplementary Table 12: Lapses estimated marginal means

	Cond	Block	Mean	CI (lower)	CI (upper)
**Avg**	Sha	Pr	5.15	4.15	6.36
	Sha	Po	3.37	2.67	4.24
	Pul	Pr	4.44	3.51	5.59
	Pul	Po	3.09	2.41	3.96
	Cen	Pr	4.49	3.48	5.77
	Cen	Po	4.73	3.67	6.07
**Min (0)**	Sha	Pr	4.09	3.03	5.51
	Sha	Po	2.85	2.02	3.99
	Pul	Pr	4.20	3.07	5.73
	Pul	Po	2.50	1.74	3.59
	Cen	Pr	4.18	3.01	5.78
	Cen	Po	4.45	3.21	6.12
**Min (slope)**	Sha	Pr	4.90	0.80	8.99
	Sha	Po	3.56	-1.39	8.50
	Pul	Pr	1.16	-3.15	5.47
	Pul	Po	4.41	-0.75	9.56
	Cen	Pr	1.52	-2.75	5.79
	Cen	Po	1.31	-2.89	5.51

*Units in percent probability of lapsing. Model-based estimates of the mean outcome for each level, averaging over and controlling for the other variables in the model. Avg represents average across all other factors. Min (slope) represents the average change in the outcome variable per minute into the task. Min (0) represents task onset (0 minutes into the task block). *Abbreviations:* Cond, condition; Sha, sham sonication; Pul, pulvinar sonication; Cen, central thalamic sonication; Pr, pre-sonication; Po, post-sonication; Min, minute into the task block.

Supplementary Table 13: Lapses estimated marginal means contrasts

Contrast	Est	CI (lower)	CI (upper)	z	p	p_{adj}	
Pul-Sha | Po-Pr	1.07	0.82	1.40	0.48	0.631	0.631	
Cen-Sha | Po-Pr	1.64	1.28	2.12	3.84	**<0.001**	**<0.001**	***
Cen-Pul | Po-Pr	1.54	1.19	1.99	3.25	**0.001**	**0.003**	**
Min (0) | Cen-Pul | Po-Pr	1.82	1.08	3.09	2.24	**0.025**	**0.050**	†
Min (slope) | Pr	1.03	1.00	1.05	2.03	**0.042**	**0.063**	†
Min (slope) | Cen-Pul | Po-Pr	0.97	0.88	1.06	-0.75	0.452	0.542	

*Units in odds ratios. Grey rows mark results presented in the main manuscript. Min (slope) represents the average change in the outcome variable per minute into the task. Min (0) represents task onset (0 minutes into the task block). *Abbreviations:* Est, estimate; CI, confidence interval; Min, minute; Pr, pre-sonication; Po, post-sonication; Sha, sham sonication; Pul, pulvinar sonication; Cen, central thalamic sonication; p_{adj}, p-value after correcting for multiple comparisons using the Benjamini-Hochberg method. † $p < 0.10$ before and/or after correction. * $p < 0.05$, ** $p < 0.01$, *** $p < 0.001$ after correction.

Egly-Driver Task (EDT)

Additional data cleaning results

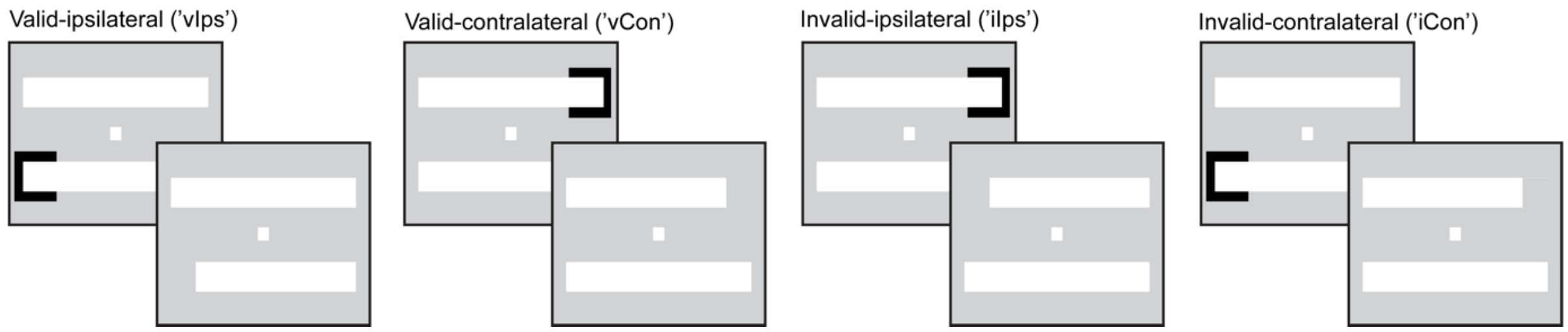

Supplementary Figure 8: **Grouping of main trials during the Egly-Driver Task (EDT) according to spatial cue validity and visual target hemifield.** We targeted the left pulvinar and left central thalamus in all participants. Stimuli could appear in the hemifield ipsilateral to the ultrasound target (left side of the display) or contralateral (right side of the monitor). There were four kinds of main trials based on cue validity and visual target location: validly cued ipsilateral targets, validly cued contralateral targets, invalidly cued ipsilateral targets, and invalidly cued contralateral targets. We considered all validly cued visual targets but limited invalidly cued target trials to those in which the spatial cue appeared in the other hemifield than the target (either same bar or not). We thus ignored main trials in which the spatial cue and visual target appeared in the same hemifield but on different bars in the main analysis. *Abbreviations:* vCon, validly cued contralateral target; iCon, invalidly cued contralateral target; vIps, validly cued ipsilateral target; iIps, invalidly cued ipsilateral target.

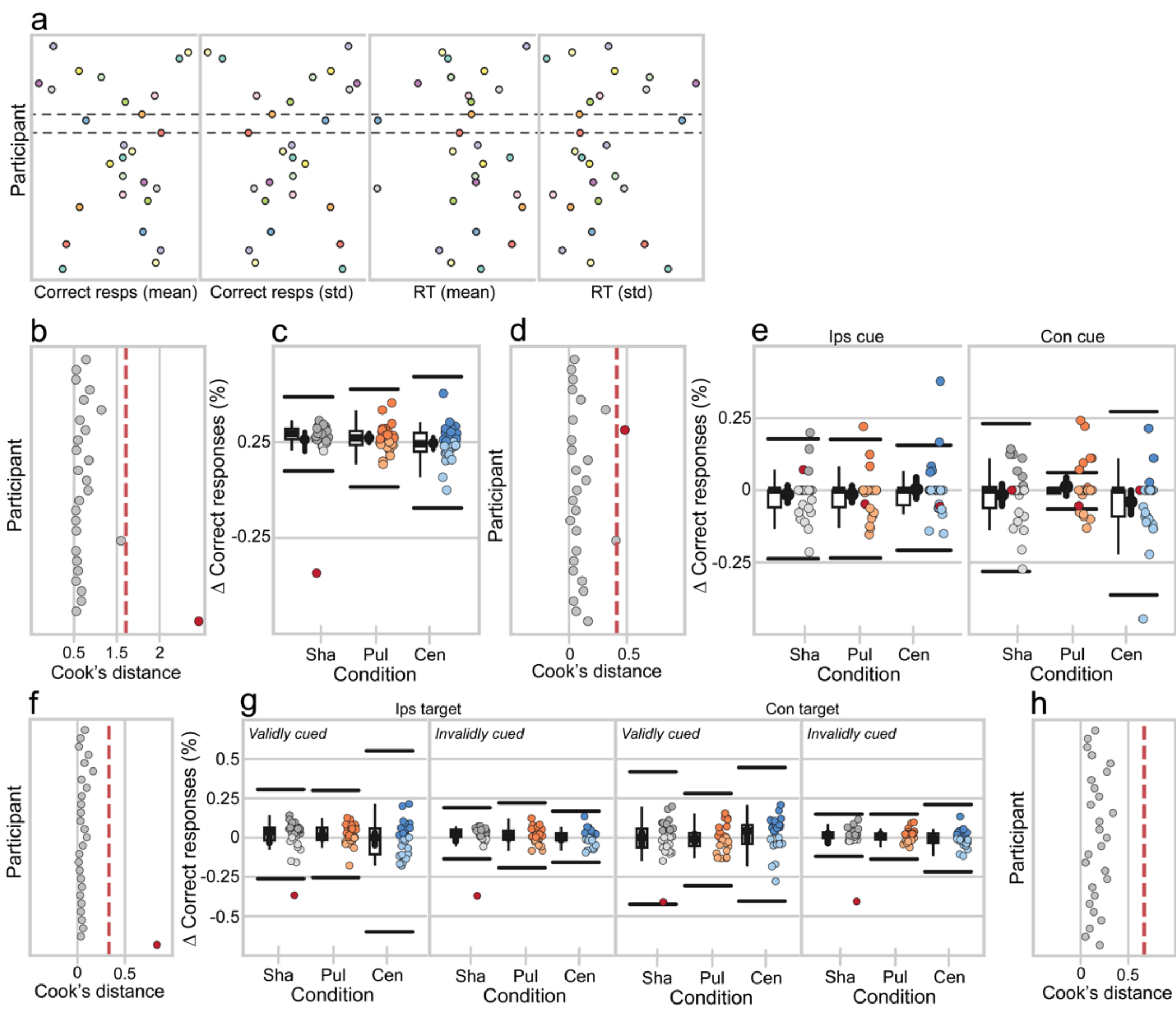

Supplementary Figure 9: **Data cleaning for the mixed-effects models examining visuospatial performance during the Egly-Driver Task (EDT).** Boxes show first quartiles, medians, and third quartiles and whiskers span the datapoints within 1.5 × the IQR from the lower and upper hinges. Black points and bars next to each box show means and 95% confidence intervals across participants. **a** Distribution of correct responses and response times on average (mean) as well as their variability (std) amongst the participants. Dashed lines mark participants we are missing button presses for in some sessions to show that they show no systematic issues in their data compared to the group. **b, d, f & h** Cook's distance for each participant in the mixed-effects models for correct responses on all trials (b), correct responses on catch trials (d), correct responses on main trials (f), and response times on main trials (h). Red dashed line marks the exclusion threshold (4 x the mean across participants). Points in red are participants who surpassed the Cook's distance exclusion threshold and underwent further evaluation. **c, e, & g** Distribution of differences in correct responses on average (c), on catch trials for each spatial cue location (e), and on main trials separated by spatial cue validity and visual target location (g). Individual points are participant means and are lighter or darker if the participant had greater value of the outcome before or after sonication, respectively. Solid black horizontal lines show outer fences based on Tukey's method (less than the first quartile minus 3 × the IQR or greater than the third quartile plus 3 × the IQR) for each condition. Participants in red are those who surpassed the Cook's distance exclusion threshold for the outcome's mixed-effects model. *Abbreviations:* Sha, sham sonication; Pul, pulvinar sonication; Cen, central thalamic sonication; Ips, ipsilateral; Con, contralateral.

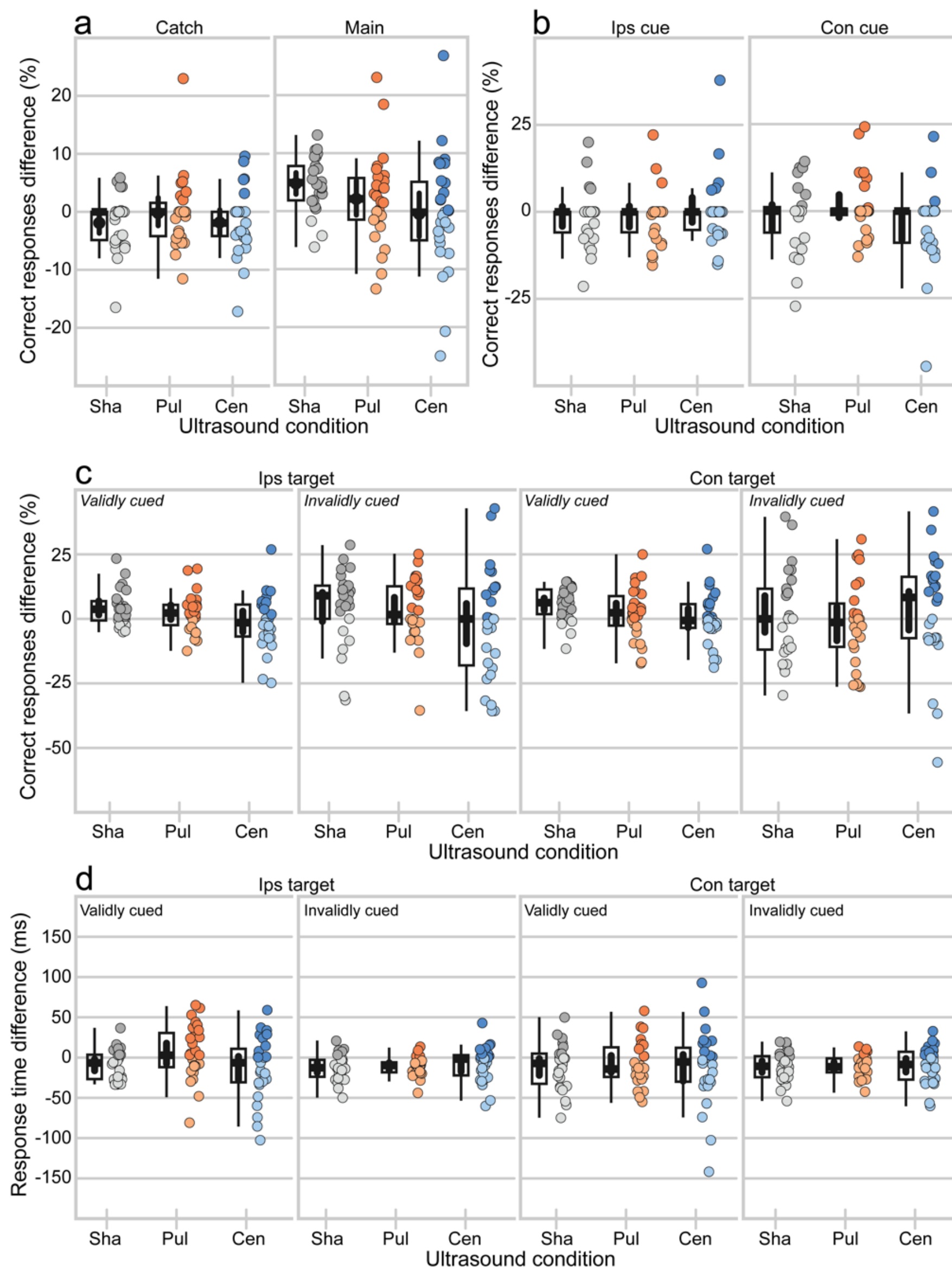

Supplementary Figure 10: **Descriptive statistics for the cleaned data used in the mixed-effects models examining visuospatial performance during the Egly-Driver Task (EDT).** Boxes show first quartiles, medians, and third quartiles and whiskers span the datapoints within 1.5 × the IQR from the lower and upper hinges. Black points and bars next to each box show means and 95% confidence intervals across participants. Individual points are participant means and are lighter or darker if the participant had greater value of the outcome before or after sonication, respectively. **a** Distribution of differences in correct responses (post- minus pre-sonication) for the different trial types, on average. **b** Distribution of differences in correct responses on catch trials for each spatial cue location. **c** Distribution of differences in correct responses on main trials for each combination of spatial cue validity and visual target hemifield. **d** Distribution of differences in response times on main trials for each combination of spatial cue validity and visual target hemifield. *Abbreviations*: Sha, sham sonication; Pul, pulvinar sonication; Cen, central thalamic sonication; Ips, ipsilateral; Con, contralateral.

Additional mixed-effects modeling results

Correct responses between trial types

There was no effect of biological sex or age on the odds of responding correctly, as indicated by the fixed effect terms ('F-M') and ('Age') in the model, respectively (see Supplementary Table 14). The random intercept for participant with the random slope for ultrasound condition captured some individual differences in baseline (pre-sonication) odds of responding correctly between the sessions (and thus ultrasound conditions), as indicated by the random slopes for ultrasound condition for each participant (see Supplementary Fig. 11b, right, and Supplementary Table 14). No variability in the odds of responding correctly based on task order was captured by the random intercept for task order (see Supplementary Fig. 11c and Supplementary Table 14).

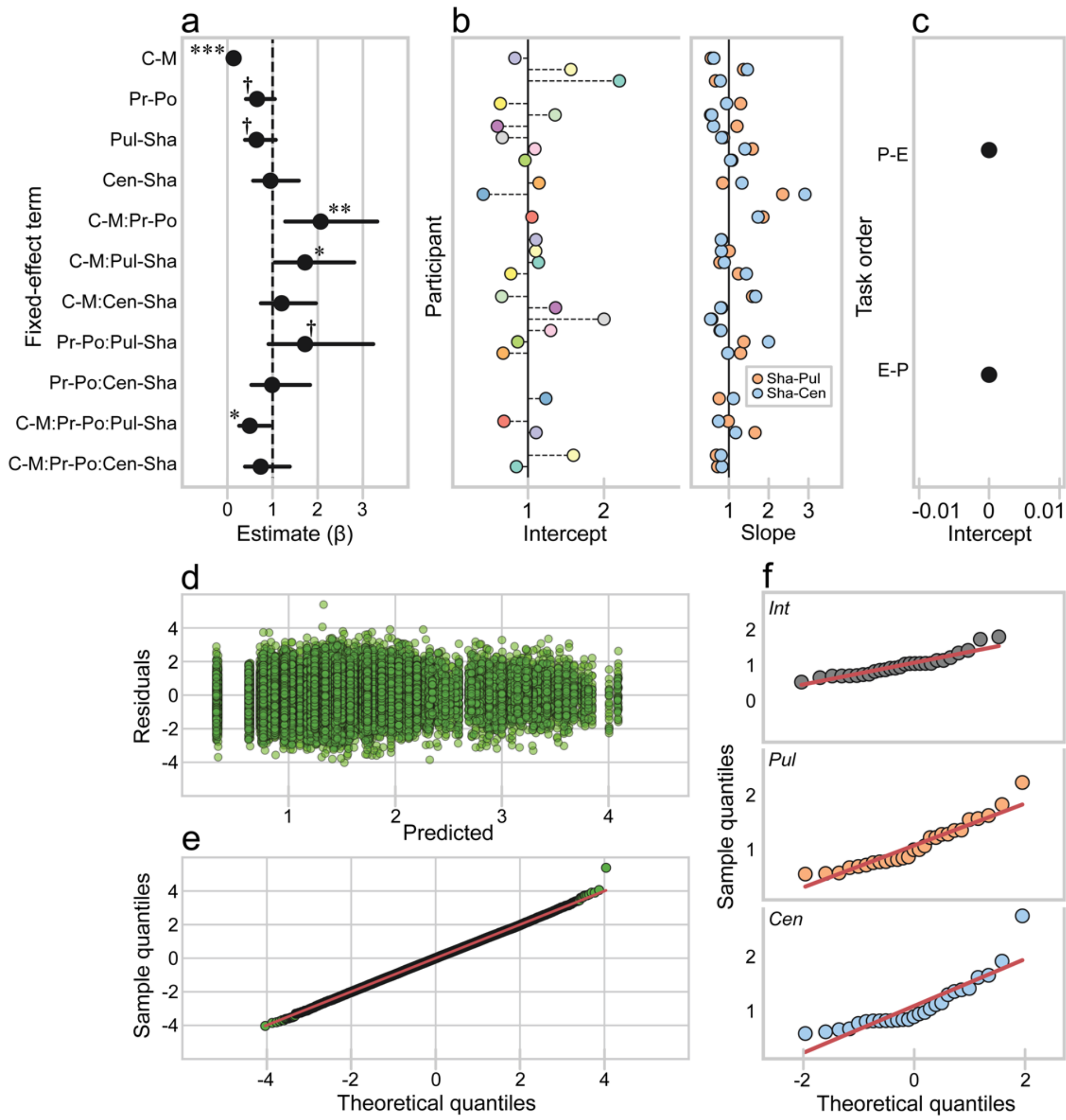

Supplementary Figure 11: **Summary of the mixed-effects logistic regression examining effects of sonication on correct responses during the Egly-Driver Task (EDT) differed for catch and main trials: correct responses ~ condition × block × trial type + sex + age + (1 + condition | participant) + (1 | task order).** **a** Fixed effect estimates. Bars show 95% confidence intervals. **b & c** Random effects. **b** Random intercept for participant (left) with a random slope for ultrasound condition (right). The random intercept for each participant shows their baseline odds of responding correctly (when block is at pre-sonication and ultrasound condition is at sham sonication) relative to the average baseline odds of responding correctly across participants in the fixed effects (0, black line). The random slope for ultrasound condition for each participant shows the difference in their baseline odds of responding correctly (when block is at pre-sonication) between the pulvinar and sham conditions (orange) and the central thalamus and sham conditions (blue) relative to the average across participants in the fixed effects (0, black line). **c** Random intercept for task order. Points show the average baseline odds of responding correctly (when block is at pre-sonication and ultrasound condition is at sham sonication) for each task order group, PVT then EDT ('P-E') and EDT then PVT ('E-P'). **d & e** Model fitness metrics. Residuals were simulated and normalized using the DHARMa package in R. **d** Predicted values versus residuals. **e** QQ plot for theoretical quantiles versus residual (sample) quantiles. **f** QQ plot showing theoretical quantiles versus sample quantiles for the random intercept for participant (top, black) as well as the random slopes for the steps in condition from sham to pulvinar sonication (middle, orange) and from sham to central thalamus sonication (bottom, blue). *Abbreviations:* Int, intercept; C, catch trial; M, main trial; Pr, pre-sonication; Po, post-sonication; Sha, sham condition; Pul, pulvinar condition; Cen, central thalamus condition.

Supplementary Table 14: Correct responses ~ trial type × block × condition + sex + age + (1 + condition | participant) + (1 | task order)

Model summary

	Marg R^2	*Cond R^2*	*ICC*				
	0.06	0.11	0.05				
Fit (ANOVA)	*AIC*	*BIC*	*Log L*	*Deviance*	*χ^2*	*df*	*p*
Null/restricted	34738	34806	-17361	34722			
Full	34080	34259	-17019	34038	683.6	11	**<0.001**
Fixed effect	*Estimate*	*95% CI*	*z*	*p*			
Int	34.07	12.55 – 92.49	6.92	**<0.001**	***		
C-M	0.14	0.09 – 0.20	-10.45	**<0.001**	***		
Pr-Po	0.65	0.41 – 1.05	-1.77	0.076	†		
Pul-Sha	0.64	0.39 – 1.07	-1.70	0.088	†		
Cen-Sha	0.95	0.57 – 1.58	-0.19	0.851			
F-M	1.14	0.63 – 2.04	0.43	0.666			
Age	0.98	0.94 – 1.03	-0.75	0.453			
C-M:Pr-Po	2.06	1.28 – 3.32	3.00	**0.003**	**		
C-M:Pul-Sha	1.72	1.05 – 2.81	2.17	**0.030**	*		
C-M:Cen-Sha	1.20	0.74 – 1.96	0.73	0.465			
Pr-Po:Pul-Sha	1.72	0.91 – 3.23	1.68	0.092	†		
Pr-Po:Cen-Sha	0.99	0.53 – 1.83	-0.03	0.973			
C-M:Pr-Po:Pul-Sha	0.49	0.26 – 0.94	-2.15	**0.031**	*		
C-M:Pr-Po:Cen-Sha	0.74	0.39 – 1.38	-0.96	0.337			
Random effect	*N (obs)*	*Factor*	*Variance*	*Std*	*Corr*		
Participant	27 (37157)	Int	1.19	1.51			
		Pul-Sha	1.22	1.57	-0.58		
		Cen-Sha	1.23	1.58	-0.41	0.78	
Task order	2	Int	1.00	1.00			

*Mixed-effects logistic regression. Units in odds ratios. An odds ratio of 1 reflects equal likelihood of responding correctly between the compared groups. Grey rows indicate results reported in the main text. ":" denotes interaction terms, reflecting the additional change in the outcome across the stated levels of the fixed effects. Trial type is a categorical factor with two levels: catch ('C') and main ('M') trials. Block is a categorical factor with two levels: before sonication ('Pr') and after sonication ('Po'). Condition is a categorical factor with three levels: sham ('Sha'), pulvinar ('Pul'), and central thalamic sonication ('Cen'). The reference levels for block and condition are pre-sonication ('Pr') and sham sonication ('Sha'), respectively. The reference level for trial type is catch ('C'). Random effects include a participant-level random intercept (baseline differences in the outcome across individuals) and a participant-level random slope for condition (individual differences in the effect of condition on the outcome at baseline, before sonication). *Abbreviations:* Marg, marginal; Cond, conditional; Log L, log likelihood; CI, confidence intervals; Int, intercept; C, catch trial; M, main trial; Pr, pre-sonication; Po, post-sonication; Sha, sham sonication; Pul, pulvinar sonication; Cen, central thalamic sonication; M, male; F, female; Obs, observations; Corr, correlation. † p < 0.10, * p < 0.05, ** p < 0.01, *** p < 0.001.

Supplementary Table 15: Correct responses estimated marginal means

Cond	Block	Trial	Mean	CI (lower)	CI (upper)
Sha	Pr	Catch	96.18	94.05	97.57
Sha	Po	Catch	94.28	91.34	96.26
Pul	Pr	Catch	94.18	91.21	96.18
Pul	Po	Catch	94.79	92.13	96.59
Cen	Pr	Catch	96.00	93.73	97.46
Cen	Po	Catch	93.95	90.93	96.01
Sha	Pr	Main	77.53	71.86	82.35
Sha	Po	Main	82.36	77.51	86.34
Pul	Pr	Main	79.21	73.92	83.65
Pul	Po	Main	81.41	76.51	85.48
Cen	Pr	Main	79.77	74.24	84.36
Cen	Po	Main	79.52	73.94	84.15

*Units in percent probability of responding correctly. Model-based estimates of the mean outcome for each level, averaging over and controlling for the other fixed effects in the model. *Abbreviations:* Cond, condition; Sha, sham sonication; Pul, pulvinar sonication; Cen, central thalamic sonication; Pr, pre-sonication; Po, post-sonication.

Supplementary Table 16: Correct responses estimated marginal means contrasts

Contrast	Est	CI (lower)	CI (upper)	z	*p*	p_{adj}	
Cen-Pul | Po-Pr | Catch	0.58	0.30	1.11	-1.65	**0.099**	0.124	†
Pul-Sha | Po-Pr | Main	0.85	0.74	0.97	-2.33	**0.020**	**0.035**	*
Cen-Sha | Po-Pr | Main	0.73	0.64	0.83	-4.62	**<0.001**	**<0.001**	***
Cen-Pul | Po-Pr | Main	0.86	0.75	0.98	-2.31	**0.021**	**0.035**	*
Cen-Pul | Po-Pr | Main-Catch	1.49	0.73	3.03	1.09	0.275	0.344	

*Units in odds ratios. An odds ratio of 1 reflects equal likelihood of responding correctly between compared groups. Grey rows mark results presented in the main manuscript. *Abbreviations:* Est, estimate; CI, confidence interval; Pr, pre-sonication; Po, post-sonication; Sha, sham sonication; Pul, pulvinar sonication; Cen, central thalamic sonication; p_{adj}, p-value after correcting for multiple comparisons using the Benjamini-Hochberg method. † $p < 0.10$ before and/or after correction. * $p < 0.05$, ** $p < 0.01$, *** $p < 0.001$ after correction.

Correct responses on catch trials

There was no effect of biological sex or age on the odds of responding correctly, as indicated by the fixed effect terms ('F-M') and ('Age') in the model, respectively (see Supplementary Table 17). The random intercept for participant with the random slope for ultrasound condition captured some individual differences in the odds of responding correctly at baseline (pre-sonication) between the sessions (and thus ultrasound conditions), as indicated by the random slopes for the ultrasound conditions for each participant (see Supplementary Fig. 12b, right, and Supplementary Table 17). No variability in the odds of responding correctly based on task order was captured by the random intercept for task order included in the model (see Supplementary Fig. 12c and Supplementary Table 17).

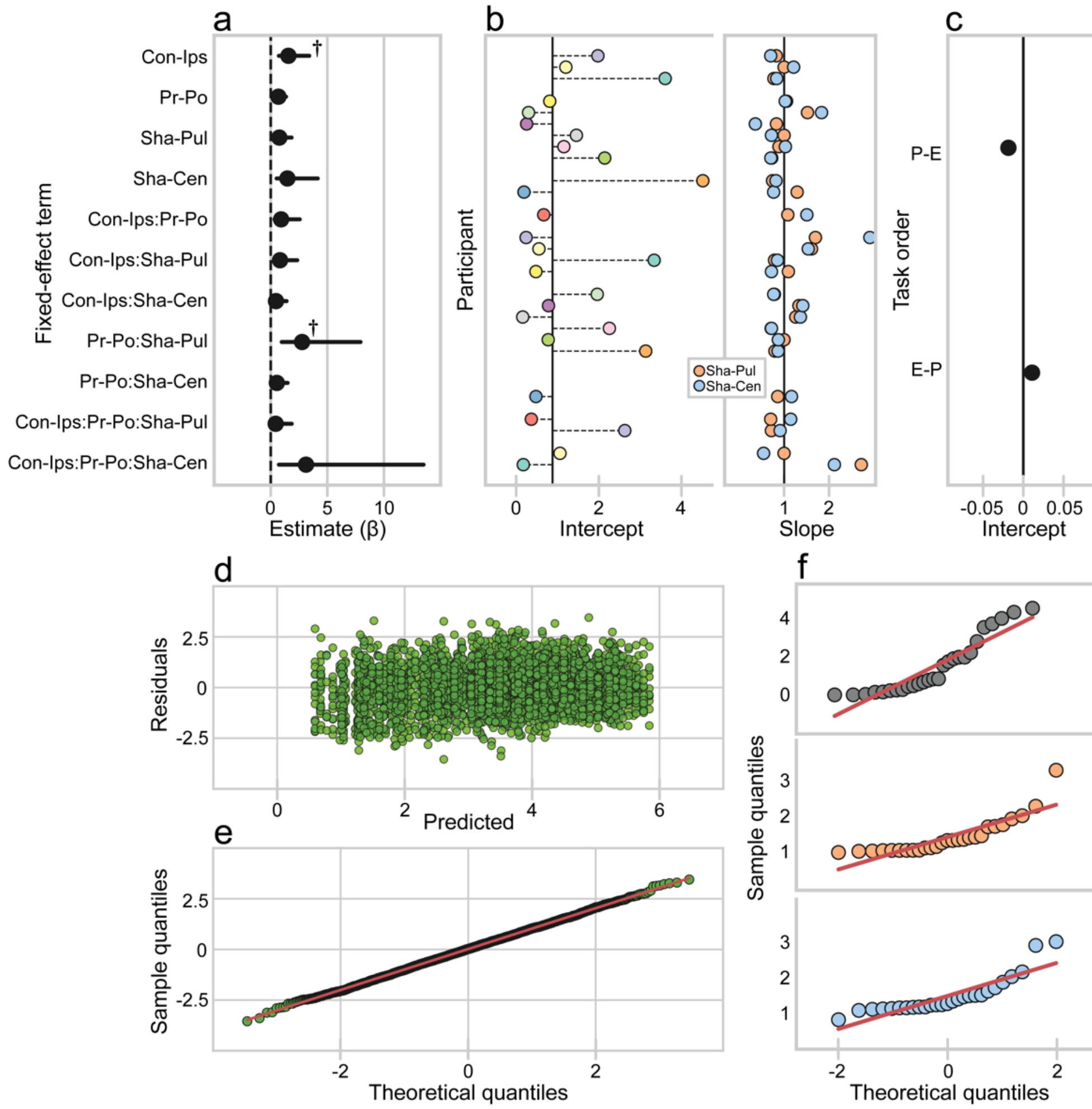

Supplementary Figure 12: **Summary of the mixed-effects logistic regression examining whether the effects of sonication in the ultrasound conditions on correct responses on catch trials during the Egly-Driver Task (EDT) depended on spatial cue location: correct responses ~ condition × block × spatial cue location + sex + age + (1 + condition | participant) + (1 | task order)**. **a** Fixed effect estimates. Bars show 95% confidence intervals. **b & c** Random effects. **b** Random intercept for participant (left) with a random slope for ultrasound condition (right). The random intercept for each participant shows their baseline odds of responding correctly (when block is at pre-sonication and ultrasound condition is at sham) relative to the average baseline odds of responding correctly across participants in the fixed effects (0, black line). The random slope for ultrasound condition for each participant shows the difference in their baseline odds of responding correctly (when block is at pre-sonication) between the pulvinar and sham sonication conditions (orange) and the central thalamus and sham sonication conditions (blue) relative to the average across participants in the fixed effects (0, black line). **c** Random intercept for task order. Points show the average baseline odds of responding correctly (when block is at pre-sonication and ultrasound condition is at sham) for each task order group, PVT then EDT ('P-E') and EDT then PVT ('E-P'). **d & e** Model fitness metrics. Residuals were simulated and normalized using the DHARMa package in R. **d** Predicted values versus residuals. **e** QQ plot for theoretical quantiles versus residual (sample) quantiles. **f** QQ plot showing theoretical quantiles versus sample quantiles for the random intercept for participant (top, black) as well as the random slopes for the steps in condition from sham to pulvinar sonication (middle, orange) and from sham to central thalamus sonication (bottom, blue). *Abbreviations:* Int, intercept; Pr, pre-sonication; Po, post-sonication; Sha, sham sonication; Pul, pulvinar sonication; Cen, central thalamic sonication; Ips, ipsilateral spatial cue; Con, contralateral spatial cue.

Supplementary Table 17: Correct responses ~ spatial cue location × block × condition + sex + age + (1 + condition | participant) + (1 | task order)

Model summary

	Marg R^2	*Cond R^2*	*ICC*				
	0.04	0.37	0.34				
Fit (ANOVA)	*AIC*	*BIC*	*Log L*	*Deviance*	*χ^2*	*df*	*p*
Null/restricted	1409.3	1459.2	-696.66	1393.3			
Full	1416.8	1547.7	-687.37	1374.8	18.57	13	0.14
Fixed effect	*Odds ratio*	*95% CI*	*z*	*p*			
Int	26.63	0.67 – 1061.58	1.75	0.081	†		
Con-Ips	1.56	0.72 – 3.40	1.12	0.264			
Pr-Po	0.68	0.34 – 1.35	-1.10	0.269			
Sha-Pul	0.76	0.31 – 1.84	-0.62	0.538			
Sha-Cen	1.47	0.52 – 4.15	0.73	0.467			
F-M	4.18	0.47 – 37.48	1.28	0.202			
Age	0.96	0.81 – 1.14	-0.45	0.651			
Con-Ips:Pr-Po	0.91	0.33 – 2.56	-0.17	0.864			
Con-Ips:Sha-Pul	0.82	0.28 – 2.34	-0.38	0.706			
Con-Ips:Sha-Cen	0.46	0.15 – 1.40	-1.37	0.172			
Pr-Po:Sha-Pul	2.77	0.97 – 7.93	1.90	0.058	†		
Pr-Po:Sha-Cen	0.53	0.19 – 1.49	-1.20	0.231			
Con-Ips:Pr-Po:Sha-Pul	0.43	0.10 – 1.85	-1.13	0.258			
Con-Ips:Pr-Po:Sha-Cen	3.11	0.72 – 13.50	1.52	0.129			
Random effect	*N (obs)*	*Factor*	*Variance*	*Std*	*Corr*		
Participant	27 (3778)	Int	7.04	4.12			
		Pul-Sha	1.32	1.69	-0.61		
		Cen-Sha	1.41	1.79	-0.51	0.78	
Task order	2	Int	1.06	1.27			

*Mixed-effects logistic regression. Units in odds ratios. An odds ratio of 1 reflects equal likelihood of responding correctly between the compared groups. Grey rows indicate results reported in the main text. ":" denotes interaction terms, reflecting the additional change in the outcome across the stated levels of the fixed effects. Spatial cue hemifield is a categorical factor with two levels: contralateral ('Con') and ipsilateral ('Ips') trials. Block is a categorical factor with two levels: before sonication ('Pr') and after sonication ('Po'). Condition is a categorical factor with three levels: sham ('Sha'), pulvinar ('Pul'), and central thalamic sonication ('Cen'). The reference levels for block and condition are pre-sonication ('Pr') and sham sonication ('Sha'), respectively. The reference level for spatial cue hemifield was contralateral ('Con'). Random effects include a participant-level random intercept (baseline differences in the outcome across individuals) and a participant-level random slope for condition (individual differences in the effect of condition on the outcome at baseline, before sonication). *Abbreviations:* Marg, marginal; Cond, conditional; Log L, log likelihood; CI, confidence intervals; Int, intercept; Pr, pre-sonication; Po, post-sonication; Sha, sham sonication; Pul, pulvinar sonication; Cen, central thalamic sonication; M, male; F, female; Ips, ipsilateral spatial cue; Con, contralateral spatial cue; Obs, observations; Corr, correlation. † $p < 0.10$, * $p < 0.05$, ** $p < 0.01$, *** $p < 0.001$.

Supplementary Table 18: Catch trial correct responses estimated marginal means

Cond	Block	Cue	Mean	CI (lower)	CI (upper)
Sha	Pr	Con	95.56	85.07	98.78
Sha	Po	Con	93.59	79.90	98.17
Sha	Pr	Ips	97.10	89.69	99.23
Sha	Po	Ips	95.41	84.78	98.73
Pul	Pr	Con	94.20	82.41	98.26
Pul	Po	Con	96.83	89.43	99.10
Pul	Pr	Ips	95.38	85.84	98.60
Pul	Po	Ips	93.89	82.15	98.09
Cen	Pr	Con	96.93	89.36	99.17
Cen	Po	Con	91.97	76.85	97.53
Cen	Pr	Ips	95.77	86.39	98.78
Cen	Po	Ips	95.90	86.75	98.82

*Units in percent probability of responding correctly. Model-based estimates of the mean outcome for each level, averaging over and controlling for the other fixed effects in the model. *Abbreviations:* Cond, condition; Sha, sham sonication; Pul, pulvinar sonication; Cen, central thalamic sonication; Pr, pre-sonication; Po, post-sonication; Con, contralateral spatial cue; Ips, ipsilateral spatial cue.

Supplementary Table 19: Catch trial correct responses estimated marginal means contrasts

Contrast	Est	CI (lower)	CI (upper)	z	*p*	p_{adj}	
Pul-Sha | Po-Pr | Ips	1.20	0.44	3.27	0.35	0.724	0.724	
Cen-Sha | Po-Pr | Ips	1.66	0.58	4.78	0.95	0.344	0.482	
Pul-Cen | Po-Pr | Con	5.18	1.72	15.65	2.92	0.004	0.028	*
Pul-Cen | Po-Pr | Ips	0.72	0.27	1.89	-0.67	0.506	0.590	
Pul | Po-Pr | Ips-Con	0.40	0.14	1.10	-1.77	0.076	0.133	†
Cen | Po-Pr | Ips-Con	2.85	1.00	8.10	1.96	0.050	0.117	†
Pul-Cen | Po-Pr | Ips-Con	0.14	0.03	0.60	-2.64	0.008	0.028	*

*Units in odds ratios. An odds ratio of 1 reflects equal likelihood of responding correctly between compared groups. Grey rows mark results presented in the main manuscript. *Abbreviations:* Est, estimate; CI, confidence interval; Pr, pre-sonication; Po, post-sonication; Sha, sham sonication; Pul, pulvinar sonication; Cen, central thalamic sonication; p_{adj}, p-value after correcting for multiple comparisons using the Benjamini-Hochberg method. † $p < 0.10$ before and/or after correction. * $p < 0.05$, ** $p < 0.01$, *** $p < 0.001$ after correction.

Correct responses on main trials

There was no effect of biological sex or age on the odds of responding correctly, as indicated by the fixed effect terms ('F-M') and ('Age') in the model, respectively (see Supplementary Table 20). The random intercept for participant with the random slope for ultrasound condition captured some individual differences in the odds of responding correctly at baseline (pre-sonication) between the sessions (and thus ultrasound conditions), as indicated by the random slopes for the ultrasound conditions for each participant (see Supplementary Fig. 13b, right, and Supplementary Table 20). No variability in the odds of responding correctly based on task order was captured by the random intercept for task order included in the model (see Supplementary Fig. 13c and Supplementary Table 20).

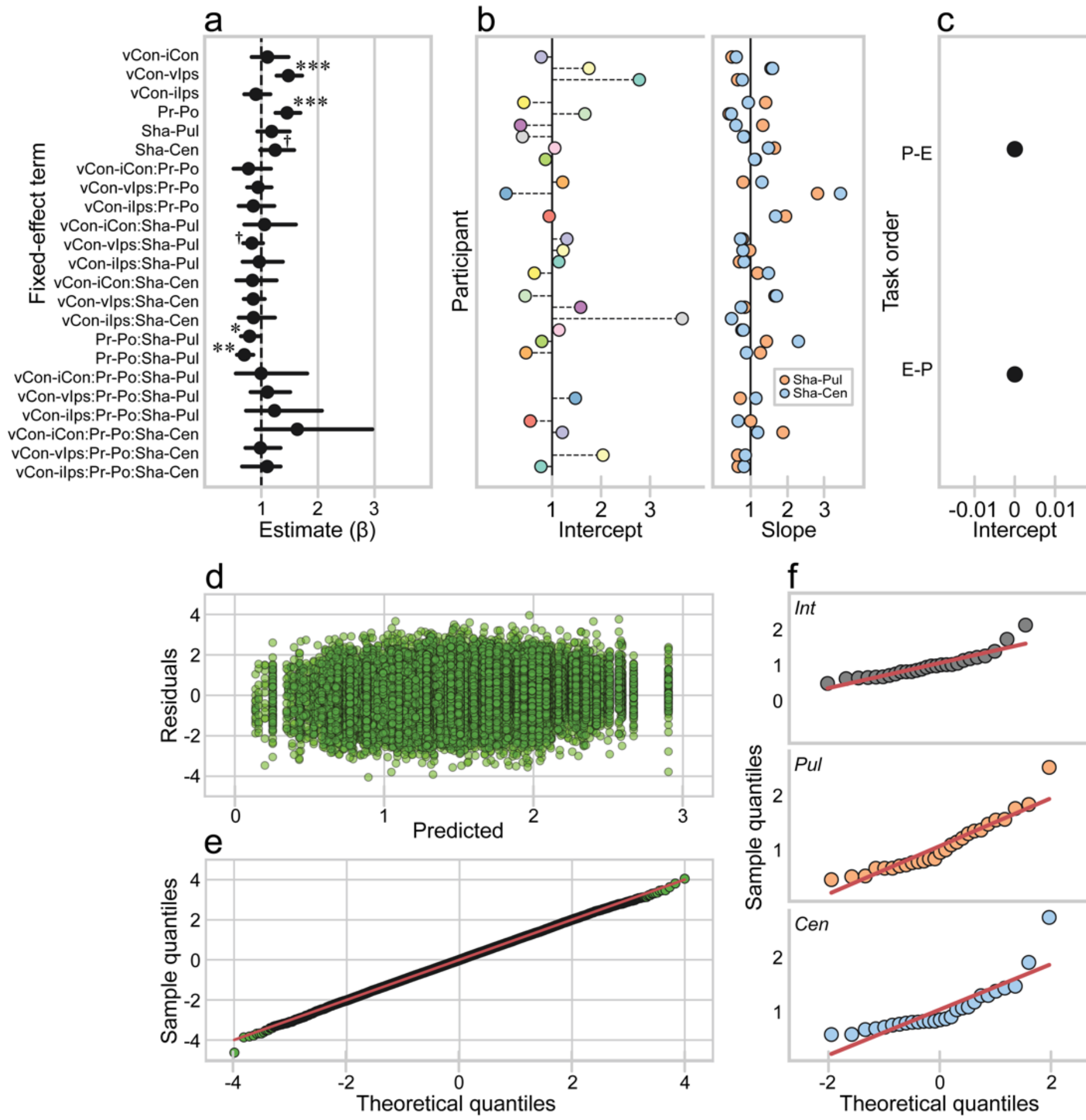

Supplementary Figure 13**: Summary of the mixed-effects logistic regression examining whether the effects of sonication in the ultrasound conditions on correct responses on main trials during the Egly-Driver Task (EDT) depended on cue-target location: correct responses ~ condition × block × cue-target location + sex + age + (1 + condition | participant) + (1 | task order). a** Fixed effect estimates. Bars show 95% confidence intervals. **b & c** Random effects. **b** Random intercept for participant (left) with a random slope for ultrasound condition (right). The random intercept for each participant shows their baseline odds of responding correctly (when block is at pre-sonication and ultrasound condition is at sham) relative to the average baseline odds of responding correctly across participants in the fixed effects (0, black line). The random slope for ultrasound condition for each participant shows the difference in their baseline odds of responding correctly (when block is at pre-sonication) between the pulvinar and sham sonication conditions (orange) and the central thalamus and sham sonication conditions (blue) relative to the average across participants in the fixed effects (0, black line). **c** Random intercept for task order. Points show the average baseline odds of responding correctly (when block is at pre-sonication and ultrasound condition is at sham) for each task order group, PVT then EDT (‘P-E’) and EDT then PVT (‘E-P’). **d & e** Model fitness metrics. Residuals were simulated and normalized using the DHARMa package in R. **d** Predicted values versus residuals. **e** QQ plot for theoretical quantiles versus residual (sample) quantiles. **f** QQ plot showing theoretical quantiles versus sample quantiles for the random intercept for participant (top, black) as well as the random slopes for the steps in condition from sham to pulvinar sonication (middle, orange) and from sham to central thalamus sonication (bottom, blue). *Abbreviations:* Int, intercept; Pr, pre-sonication; Po, post-sonication; Sha, sham sonication; Pul, pulvinar sonication; Cen, central thalamic sonication; vCon, validly cued contralateral target; iCon, invalidly cued contralateral target; vIps, validly cued ipsilateral target; iIps, invalidly cued ipsilateral target.

Supplementary Table 20: Correct responses ~ cue-target location × block × condition + sex + age + (1 + condition | participant) + (1 | task order)

Model summary

	Marg R^2	*Cond R^2*					
	0.012	0.07					
Fit (ANOVA)	*AIC*	*BIC*	*Log L*	*Deviance*	*χ^2*	*df*	*p*
Null/restricted	29902	29968	-14943	29886			
Full	29788	30063	-14861	29722	163.9	23	**<0.001**
Fixed effect	*Estimate*	*95% CI*		*z*	*p*		
Int	4.21	1.46 – 12.17		2.66	**0.008**	**	
vCon-iCon	1.11	0.83 – 1.48		0.71	0.476		
vCon-vIps	1.48	1.27 – 1.72		5.09	**<0.001**	***	
vCon-iIps	0.90	0.70 – 1.16		-0.79	0.428		
Pr-Po	1.45	1.25 – 1.69		4.82	**<0.001**	***	
Sha-Pul	1.18	0.93 – 1.50		1.38	0.169		
Sha-Cen	1.25	0.98 – 1.58		1.83	0.068	†	
F-M	1.13	0.61 – 2.09		0.38	0.704		
Age	0.98	0.94 – 1.03		-0.66	0.508		
vCon-iCon:Pr-Po	0.77	0.51 – 1.17		-1.21	0.225		
vCon-vIps:Pr-Po	0.94	0.75 – 1.18		-0.52	0.603		
vCon-iIps:Pr-Po	0.86	0.60 – 1.23		-0.83	0.409		
vCon-iCon:Sha-Pul	1.06	0.70 – 1.61		0.26	0.793		
vCon-vIps:Sha-Pul	0.83	0.68 – 1.03		-1.69	0.091	†	
vCon-iIps:Sha-Pul	0.96	0.67 – 1.38		-0.20	0.844		
vCon-iCon:Sha-Cen	0.84	0.56 – 1.27		-0.82	0.412		
vCon-vIps:Sha-Cen	0.86	0.69 – 1.06		-1.43	0.152		
vCon-iIps:Sha-Cen	0.86	0.60 – 1.24		-0.81	0.420		
Pr-Po:Sha-Pul	0.79	0.64 – 0.98		-2.15	**0.031**	*	
Pr-Po:Sha-Cen	0.70	0.57 – 0.86		-3.34	**0.001**	**	
vCon-iCon:Pr-Po:Sha-Pul	1.00	0.55 – 1.81		-0.02	0.988		
vCon-vIps:Pr-Po:Sha-Pul	1.11	0.81 – 1.51		0.66	0.511		
vCon-iIps:Pr-Po:Sha-Pul	1.23	0.73 – 2.07		0.79	0.429		
vCon-iCon:Pr-Po:Sha-Cen	1.64	0.90 – 2.96		1.63	0.103		
vCon-vIps:Pr-Po:Sha-Cen	0.98	0.72 – 1.34		-0.11	0.915		
vCon-iIps:Pr-Po:Sha-Cen	1.10	0.66 – 1.84		0.38	0.703		
Random effect	*N (obs)*	*Factor*		*Variance*	*Std*	*Corr*	
Participant	27 (31378)	Int		1.22	1.56		
		Pul-Sha		1.28	1.64	-0.60	
		Cen-Sha		1.27	1.63	-0.45	0.76
Task order	2	Int		1.00	1.00		

*Mixed-effects logistic regression. Units in odds ratios. An odds ratio of 1 reflects equal likelihood of responding correctly between the compared groups. Grey rows indicate results reported in the main text. ":" denotes interaction terms, reflecting the additional change in the outcome across the stated levels of the fixed effects. Cue-target location is a categorical factor with four levels: validly cued contralateral targets ('vCon'), invalidly cued contralateral targets ('iCon'), validly cued ipsilateral targets ('vIps'), and invalidly cued ipsilateral targets ('iIps'). Block is a categorical factor with two levels: before sonication ('Pr') and after sonication ('Po'). Condition is a categorical factor with three levels: sham ('Sha'), pulvinar ('Pul'), and central thalamic sonication ('Cen'). The reference levels for block and condition are pre-sonication ('Pr') and sham sonication ('Sha'), respectively. The reference level for cue-target location was validly cued contralateral targets ('vCon'). Random effects include a participant-level random intercept (baseline differences in the outcome across individuals) and a participant-level random slope for condition (individual differences in the

effect of condition on the outcome at baseline, before sonication). *Abbreviations:* Marg, marginal; Cond, conditional; Log L, log likelihood; CI, confidence intervals; Int, intercept; Pr, pre-sonication; Po, post-sonication; Sha, sham sonication; Pul, pulvinar sonication; Cen, central thalamic sonication; M, male; F, female; vCon, validly cued contralateral target; iCon, invalidly cued contralateral target; vIps, validly cued ipsilateral target; iIps, invalidly cued ipsilateral target; Obs, observations; Corr, correlation. † $p < 0.10$, * $p < 0.05$, ** $p < 0.01$, *** $p < 0.001$.

Supplementary Table 21: Main trial correct responses estimated marginal means

Cond	Block	Trial	Mean	CI (lower)	CI (upper)
Sha	Pr	iCon	77.57	69.81	83.80
Sha	Po	iCon	79.56	72.05	85.46
Sha	Pr	iIps	73.78	65.65	80.56
Sha	Po	iIps	77.83	70.34	83.86
Sha	Pr	vCon	75.70	69.22	81.19
Sha	Po	vCon	81.92	76.49	86.32
Sha	Pr	vIps	82.16	76.80	86.50
Sha	Po	vIps	86.32	81.87	89.81
Pul	Pr	iCon	81.22	74.05	86.75
Pul	Po	iCon	79.35	71.75	85.32
Pul	Pr	iIps	76.24	68.55	82.53
Pul	Po	iIps	79.65	72.62	85.24
Pul	Pr	vCon	78.65	72.80	83.53
Pul	Po	vCon	80.94	75.49	85.42
Pul	Pr	vIps	81.95	76.70	86.24
Pul	Po	vIps	84.56	79.84	88.33
Cen	Pr	iCon	78.43	70.58	84.64
Cen	Po	iCon	82.39	75.21	87.82
Cen	Pr	iIps	75.11	66.93	81.82
Cen	Po	iIps	74.42	66.31	81.13
Cen	Pr	vCon	79.51	73.50	84.44
Cen	Po	vCon	79.78	73.82	84.66
Cen	Pr	vIps	83.09	77.80	87.32
Cen	Po	vIps	82.24	76.78	86.64

*Units in percent probability of responding correctly. Model-based estimates of the mean outcome for each level, averaging over and controlling for the other fixed effects in the model. *Abbreviations:* Cond, ultrasound condition; CI, confidence interval; Sha, sham sonication; Pul, pulvinar sonication; Cen, central thalamic sonication; Pr, pre-sonication; Po, post-sonication; vCon, validly cued contralateral target; iCon, invalidly cued contralateral target; vIps, validly cued ipsilateral target; iIps, invalidly cued ipsilateral target.

Supplementary Table 22: Correct responses estimated marginal means contrasts

Contrast	Est	CI (lower)	CI (upper)	z	p	p_{adj}	
Pr | Val - Inv	1.21	1.08	1.35	3.24	**0.001**	**0.018**	*
Cen-Pul | Po-Pr | vCon	0.88	0.72	1.08	-1.19	0.232	0.558	
Pul-Sha | Po-Pr | iCon	0.79	0.45	1.38	-0.83	0.407	0.712	
Cen-Sha | Po-Pr | iCon	1.14	0.66	1.99	0.48	0.635	0.868	
Cen-Pul | Po-Pr | iCon	1.45	0.82	2.56	1.28	0.200	0.558	
Pul-Sha | Po-Pr | vIps	0.88	0.70	1.11	-1.09	0.274	0.558	
Cen-Sha | Po-Pr | vIps	0.69	0.55	0.86	-3.24	**0.001**	**0.018**	*
Cen-Pul | Po-Pr | vIps	0.78	0.63	0.97	-2.21	**0.027**	0.315	†
Pul-Sha | Po-Pr | iIps	0.98	0.61	1.57	-0.09	0.926	0.968	
Cen-Sha | Po-Pr | iIps	0.77	0.48	1.23	-1.08	0.279	0.558	
Cen-Pul | Po-Pr | iIps	0.79	0.49	1.27	-0.97	0.331	0.610	
Pul | Po-Pr | iCon - vCon	0.77	0.50	1.18	-1.19	0.235	0.558	
Pul | Po-Pr | vIps - vCon	1.05	0.84	1.30	0.41	0.684	0.868	
Pul | Po-Pr | vIps - iCon	1.36	0.88	2.10	1.38	0.168	0.558	
Pul | Po-Pr | iIps - vCon	1.06	0.73	1.53	0.30	0.768	0.868	
Pul | Po-Pr | iIps - iCon	1.37	0.81	2.33	1.17	0.241	0.558	
Pul | Po-Pr | iIps - vIps	1.01	0.69	1.47	0.06	0.953	0.968	
Cen | Po-Pr | iCon - vCon	1.27	0.83	1.93	1.09	0.275	0.558	
Cen | Po-Pr | vIps - vCon	0.93	0.75	1.14	-0.71	0.478	0.760	
Cen | Po-Pr | vIps - iCon	0.73	0.48	1.12	-1.43	0.152	0.558	
Cen | Po-Pr | iIps - vCon	0.95	0.66	1.36	-0.29	0.769	0.868	
Cen | Po-Pr | iIps - iCon	0.75	0.45	1.25	-1.10	0.272	0.558	
Cen | Po-Pr | iIps - vIps	1.02	0.71	1.47	0.12	0.905	0.968	
Cen-Pul | Po-Pr | iCon - vCon	1.64	0.90	3.01	1.61	0.107	0.540	
Cen-Pul | Po-Pr | vIps - vCon	0.89	0.66	1.20	-0.79	0.431	0.718	
Pul-Sha | Po-Pr | vIps - iCon	1.11	0.61	2.04	0.35	0.724	0.868	
Cen-Sha | Po-Pr | vIps - iCon	0.60	0.33	1.09	-1.67	**0.096**	0.540	†
Cen-Pul | Po-Pr | vIps - iCon	0.54	0.29	0.99	-1.99	**0.047**	0.411	†
Cen-Pul | Po-Pr | iIps - vCon	0.90	0.53	1.50	-0.42	0.677	0.868	
Pul-Sha | Po-Pr | iIps - iCon	1.24	0.60	2.58	0.57	0.567	0.863	
Cen-Sha | Po-Pr | iIps - iCon	0.68	0.33	1.39	-1.06	0.287	0.558	
Cen-Pul | Po-Pr | iIps - iCon	0.55	0.26	1.14	-1.61	0.108	0.540	
Pul-Sha | Po-Pr | iIps - vIps	1.11	0.66	1.88	0.39	0.695	0.868	
Cen-Sha | Po-Pr | iIps - vIps	1.12	0.67	1.89	0.44	0.661	0.868	
Cen-Pul | Po-Pr | iIps - vIps	1.01	0.60	1.70	0.04	0.968	0.968	

*Units in odds ratios. An odds ratio of 1 reflects equal likelihood of responding correctly between compared groups. Grey rows mark results presented in the main manuscript. *Abbreviations:* Est, estimate; CI, confidence interval; Sha, sham sonication; Pul, pulvinar sonication; Cen, central thalamic sonication; Pr, pre-sonication; Po, post-sonication; vCon, validly cued contralateral target; iCon, invalidly cued contralateral target; vIps, validly cued ipsilateral target; iIps, invalidly cued ipsilateral target; p_{adj}, p-value after correcting for multiple comparisons using the Benjamini-Hochberg method. † $p < 0.10$ before and/or after correction. * $p < 0.05$, ** $p < 0.01$, *** $p < 0.001$ after correction.

Response time on main trials

There was no effect of biological sex or age on response times, as indicated by the fixed effect terms (‘F-M’) and (‘Age’) in the model, respectively (see Supplementary Table 23). The random intercept for participant with the random slope for ultrasound condition captured some individual differences in response times at baseline (pre-sonication) between the sessions (and thus ultrasound conditions), as indicated by the random slopes for the ultrasound conditions for each participant (see Supplementary Fig. 14b, right, and Supplementary Table 23). No variability in response times based on task order was captured by the random intercept for task order included in the model (see Supplementary Fig. 14c and Supplementary Table 23).

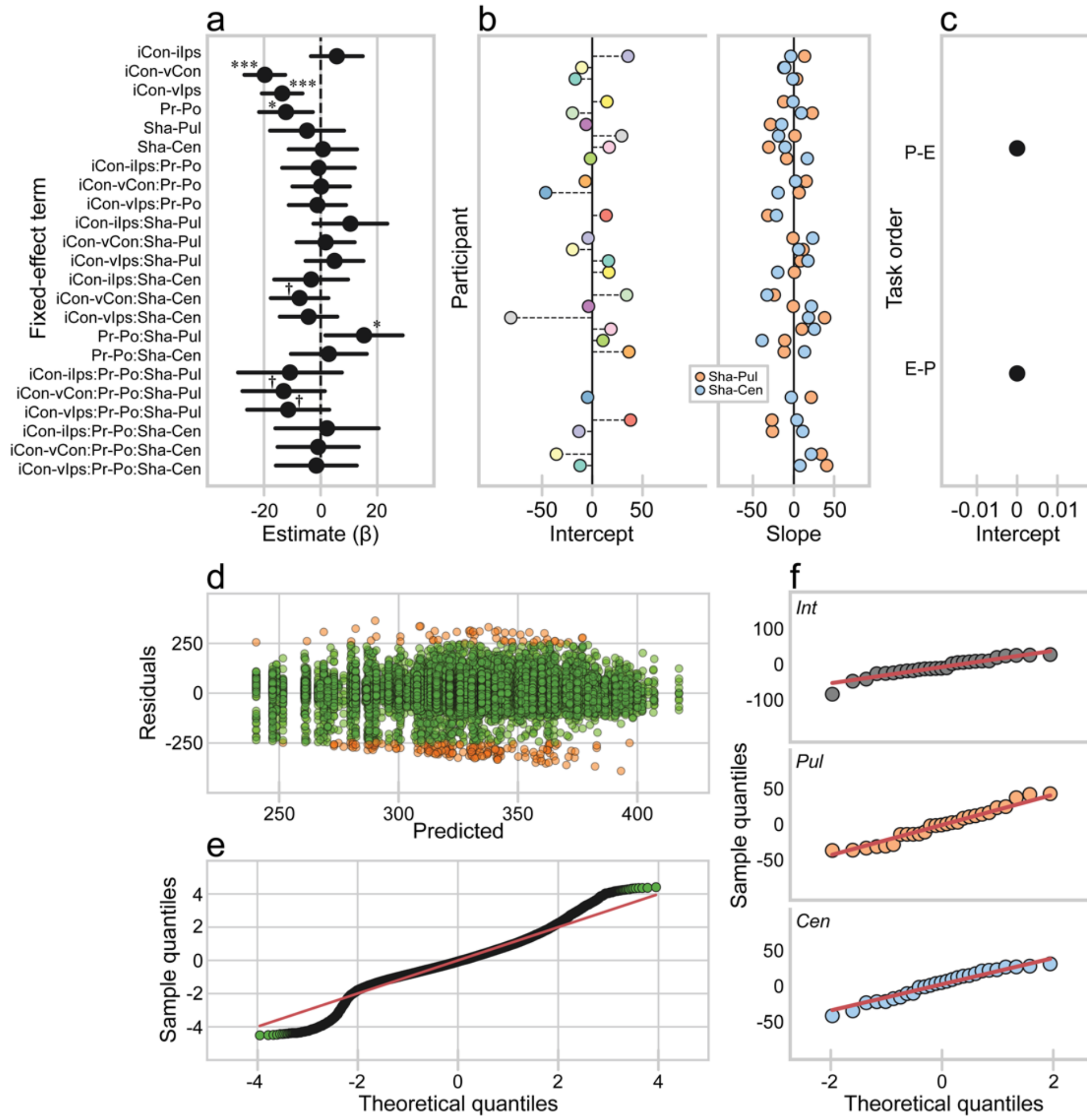

Supplementary Figure 14: **Summary of linear mixed-effects model examining whether the effects of sonication in the ultrasound conditions on response times from main trials during the Egly-Driver Task (EDT) depended on cue-target location: response time ~ condition × block × cue-target location + sex + age + (1 + condition | participant) + (1 | task order).** **a** Fixed effect estimates. Bars show 95% confidence intervals. **b & c** Random effects. **b** Random intercept for participant (left) with a random slope for ultrasound condition (right). The random intercept for each participant shows their baseline response time (when block is at pre-sonication and ultrasound condition is at sham) relative to the average baseline response time across participants in the fixed effects (0, black line). The random slope for ultrasound condition for each participant shows the difference in their baseline response time (when block is at pre-sonication) between the pulvinar and sham sonication conditions (orange) and the central thalamus and sham sonication conditions (blue) relative to the average across participants in the fixed effects (0, black line). **c** Random intercept for task order. Points show the average baseline response time (when block is at pre-sonication and ultrasound condition is at sham) for each task order group, PVT then EDT ('P-E') and EDT then PVT ('E-P'). **d & e** Model fitness metrics. Residuals were simulated and normalized using the DHARMa package in R. **d** Predicted values versus residuals. Observations with standardized residuals that exceeded ± 4 standard deviations from zero (orange) were left out of the final model. **e** QQ plot for theoretical quantiles versus residual (sample) quantiles. **f** QQ plot showing theoretical quantiles versus sample quantiles for the random intercept for participant (top, black) as well as the random slopes for the steps in condition from sham to pulvinar sonication (middle, orange) and from sham to central thalamus sonication (bottom, blue). *Abbreviations:* Int, intercept; Pr, pre-sonication; Po, post-sonication; Sha, sham sonication; Pul, pulvinar sonication; Cen, central thalamic sonication; vCon, validly cued contralateral target; iCon, invalidly cued contralateral target; vIps, validly cued ipsilateral target; iIps, invalidly cued ipsilateral target.

Supplementary Table 23: Response time ~ cue-target location × block × condition + sex + age + (1 + condition | participant) + (1 | task order)

**Model summary**							
	REML	*Marg R^2*	*Cond R^2*	*ICC*			
	275390.5	0.04	0.22	0.19			
Fit (ANOVA)	*AIC*	*BIC*	*Log L*	*Deviance*	*χ^2*	*df*	*p*
Null/restricted	276463	276536	-138222	276445			
Full	275557	275834	-137744	275489	956.2	25	**<0.001**
Fixed effect	*Est*	*SE*	*95% CI*		*t*	*p*	
Int	335.90	29.56	277.95 – 393.84		11.36	**<0.001**	***
iCon-vCon	-18.55	3.56	-25.53 – -11.57		-5.21	**<0.001**	***
iCon-iIps	6.68	4.53	-2.20 – 15.55		1.47	0.140	
iCon-vIps	-12.77	3.55	-19.72 – -5.81		-3.60	**<0.001**	***
Pr-Po	-9.54	4.69	-18.72 – -0.35		-2.03	**0.042**	*
Sha-Pul	-2.97	6.57	-15.85 – 9.92		-0.45	0.652	
Sha-Cen	1.73	6.01	-10.04 – 13.50		0.29	0.773	
F-M	16.06	16.51	-16.30 – 48.43		0.97	0.331	
Age	0.30	1.33	-2.30 – 2.90		0.23	0.820	
iCon-vCon:Pr-Po	-2.58	5.01	-12.39 – 7.24		-0.51	0.607	
iCon-iIps:Pr-Po	-1.82	6.34	-14.24 – 10.60		-0.29	0.774	
iCon-vIps:Pr-Po	-3.23	4.99	-13.01 – 6.55		-0.65	0.517	
iCon-vCon:Sha-Pul	0.31	5.06	-9.61 – 10.23		0.06	0.951	
iCon-iIps:Sha-Pul	7.76	6.44	-4.87 – 20.39		1.20	0.229	
iCon-vIps:Sha-Pul	3.43	5.05	-6.46 – 13.32		0.68	0.496	
iCon-vCon:Sha-Cen	-7.97	5.03	-17.82 – 1.89		-1.58	0.113	
iCon-iIps:Sha-Cen	-1.72	6.45	-14.35 – 10.91		-0.27	0.790	
iCon-vIps:Sha-Cen	-5.17	5.02	-15.00 – 4.67		-1.03	0.303	
Pr-Po:Sha-Pul	14.69	6.74	1.49 – 27.90		2.18	**0.029**	*
Pr-Po:Sha-Cen	-0.06	6.62	-13.03 – 12.92		-0.01	0.993	
iCon-vCon:Pr-Po:Sha-Pul	-12.69	7.18	-26.77 – 1.40		-1.77	**0.077**	†
iCon-iIps:Pr-Po:Sha-Pul	-9.35	9.05	-27.10 – 8.39		-1.03	0.302	
iCon-vIps:Pr-Po:Sha-Pul	-11.43	7.16	-25.47 – 2.60		-1.60	0.110	
iCon-vCon:Pr-Po:Sha-Cen	2.04	7.07	-11.82 – 15.89		0.29	0.773	
iCon-iIps:Pr-Po:Sha-Cen	0.23	9.01	-17.43 – 17.88		0.03	0.980	
iCon-vIps:Pr-Po:Sha-Cen	1.61	7.05	-12.21 – 15.42		0.23	0.820	
Random effect	*N (obs)*	*Factor*	*Variance*		*Std*	*Corr*	
Participant	27 (25547)	Int	662.41		25.74		
		Sha-Pul	548.33		23.42	-0.58	
		Sha-Cen	374.62		19.36	-0.30	0.46
Task order	2	Int	0.00		0.00		
Residual			2792.30		52.84		

*Linear mixed-effects model fit with REML. Units in milliseconds (ms). Grey rows indicate results reported in the main text. ":" denotes interaction terms, reflecting the additional change in the outcome across the stated levels of the fixed effects. Cue-target location is a categorical factor with four levels: validly cued contralateral targets ('vCon'), invalidly cued contralateral targets ('iCon'), validly cued ipsilateral targets ('vIps'), and invalidly cued ipsilateral targets ('iIps'). Block is a categorical factor with two levels: before sonication ('Pr') and after sonication ('Po'). Condition is a categorical factor with three levels: sham ('Sha'), pulvinar ('Pul'), and central thalamic sonication ('Cen'). The reference levels for block and condition are pre-sonication ('Pr') and sham sonication ('Sha'), respectively. The reference level for cue-target location was invalidly cued contralateral targets ('iCon'). Random effects include a participant-level random intercept (baseline differences in the outcome across individuals) and a participant-level random slope for condition

(individual differences in the effect of condition on the outcome at baseline, before sonication). *Abbreviations:* Marg, marginal; Cond, conditional; Log L, log likelihood; CI, confidence intervals; Int, intercept; Pr, pre-sonication; Po, post-sonication; Sha, sham sonication; Pul, pulvinar sonication; Cen, central thalamic sonication; M, male; F, female; vCon, validly cued contralateral target; iCon, invalidly cued contralateral target; vIps, validly cued ipsilateral target; iIps, invalidly cued ipsilateral target; Obs, observations; Corr, correlation. † $p < 0.10$, * $p < 0.05$, ** $p < 0.01$, *** $p < 0.001$.

Supplementary Table 24: Main trial response time estimated marginal means

Cond	Block	Trial	Mean	SE	CI (lower)	CI (upper)
Sha	Pr	iCon	350.90	9.22	332.82	368.97
Sha	Po	iCon	341.36	9.21	323.31	359.42
Sha	Pr	iIps	357.57	9.13	339.67	375.47
Sha	Po	iIps	346.22	9.10	328.38	364.05
Sha	Pr	vCon	332.35	8.69	315.30	349.39
Sha	Po	vCon	320.23	8.69	303.19	337.27
Sha	Pr	vIps	338.13	8.69	321.10	355.16
Sha	Po	vIps	325.36	8.68	308.34	342.39
Pul	Pr	iCon	347.93	8.92	330.46	365.41
Pul	Po	iCon	353.09	8.96	335.53	370.65
Pul	Pr	iIps	362.36	8.82	345.07	379.66
Pul	Po	iIps	356.35	8.77	339.16	373.53
Pul	Pr	vCon	329.69	8.35	313.33	346.05
Pul	Po	vCon	319.58	8.35	303.21	335.95
Pul	Pr	vIps	338.60	8.35	322.24	354.96
Pul	Po	vIps	329.09	8.34	312.74	345.44
Cen	Pr	iCon	352.63	9.37	334.26	371.00
Cen	Po	iCon	343.04	9.35	324.70	361.37
Cen	Pr	iIps	357.58	9.31	339.33	375.84
Cen	Po	iIps	346.40	9.27	328.23	364.56
Cen	Pr	vCon	326.11	8.85	308.77	343.44
Cen	Po	vCon	315.97	8.85	298.63	333.32
Cen	Pr	vIps	334.70	8.84	317.36	352.03
Cen	Po	vIps	323.48	8.84	306.15	340.81

*Units in milliseconds (ms). Model-based estimates of the mean outcome for each level, averaging over and controlling for the other fixed effects in the model. *Abbreviations:* Cond, ultrasound condition; CI, confidence interval; Sha, sham sonication; Pul, pulvinar sonication; Cen, central thalamic sonication; Pr, pre-sonication; Po, post-sonication; vCon, validly cued contralateral target; iCon, invalidly cued contralateral target; vIps, validly cued ipsilateral target; iIps, invalidly cued ipsilateral target.

Supplementary Table 25: Main trial response time estimated marginal means contrasts

Contrast	Est	SE	CI (lower)	CI (upper)	z	p	p_{adj}	
Pr | Inv - Val	21.57	1.41	18.80	24.34	15.27	**<0.001**	**<0.001**	***
Cen-Pul | Po-Pr | iCon	-14.75	6.73	-27.94	-1.56	-2.19	**0.028**	0.245	†
Pul-Sha | Po-Pr | vCon	2.01	2.52	-2.93	6.95	0.80	0.426	0.972	
Cen-Sha | Po-Pr | vCon	1.98	2.50	-2.93	6.89	0.79	0.429	0.972	
Cen-Pul | Po-Pr | vCon	-0.03	2.48	-4.90	4.84	-0.01	0.991	0.995	
Pul-Sha | Po-Pr | iIps	5.34	6.05	-6.52	17.21	0.88	0.378	0.972	
Cen-Sha | Po-Pr | iIps	0.17	6.11	-11.81	12.14	0.03	0.978	0.995	
Cen-Pul | Po-Pr | iIps	-5.17	6.12	-17.17	6.83	-0.84	0.398	0.972	
Pul-Sha | Po-Pr | vIps	3.26	2.44	-1.52	8.04	1.34	0.182	0.796	
Cen-Sha | Po-Pr | vIps	1.55	2.43	-3.21	6.31	0.64	0.523	0.972	
Cen-Pul | Po-Pr | vIps	-1.71	2.42	-6.45	3.03	-0.71	0.480	0.972	
Pul | Po-Pr | vCon - iCon	-15.26	5.15	-25.36	-5.17	-2.96	**0.003**	**0.047**	*
Pul | Po-Pr | iIps - iCon	-11.17	6.47	-23.85	1.50	-1.73	**0.084**	0.420	†
Pul | Po-Pr | iIps - vCon	4.09	4.64	-5.00	13.19	0.88	0.378	0.972	
Pul | Po-Pr | vIps - iCon	-14.67	5.14	-24.73	-4.60	-2.86	**0.004**	**0.047**	*
Pul | Po-Pr | vIps - vCon	0.60	2.46	-4.24	5.43	0.24	0.809	0.972	
Pul | Po-Pr | vIps - iIps	-3.50	4.62	-12.55	5.56	-0.76	0.449	0.972	
Cen | Po-Pr | vCon - iCon	-0.54	4.99	-10.32	9.24	-0.11	0.914	0.995	
Cen | Po-Pr | iIps - iCon	-1.59	6.40	-14.14	10.95	-0.25	0.803	0.972	
Cen | Po-Pr | iIps - vCon	-1.05	4.70	-10.27	8.16	-0.22	0.823	0.972	
Cen | Po-Pr | vIps - iCon	-1.63	4.98	-11.38	8.13	-0.33	0.744	0.972	
Cen | Po-Pr | vIps - vCon	-1.09	2.44	-5.86	3.69	-0.45	0.656	0.972	
Cen | Po-Pr | vIps - iIps	-0.03	4.69	-9.22	9.16	-0.01	0.995	0.995	
Cen-Pul | Po-Pr | vCon - iCon	14.72	7.17	0.67	28.78	2.05	**0.040**	0.280	†
Cen-Pul | Po-Pr | iIps - iCon	9.58	9.10	-8.25	27.41	1.05	0.292	0.972	
Pul-Sha | Po-Pr | iIps - vCon	3.33	6.55	-9.51	16.18	0.51	0.611	0.972	
Cen-Sha | Po-Pr | iIps - vCon	-1.81	6.60	-14.74	11.12	-0.27	0.784	0.972	
Cen-Pul | Po-Pr | iIps - vCon	-5.15	6.61	-18.10	7.81	-0.78	0.436	0.972	
Cen-Pul | Po-Pr | vIps - iCon	13.04	7.15	-0.97	27.06	1.82	**0.068**	0.397	†
Pul-Sha | Po-Pr | vIps - vCon	1.25	3.49	-5.60	8.10	0.36	0.720	0.972	
Cen-Sha | Po-Pr | vIps - vCon	-0.43	3.48	-7.24	6.38	-0.12	0.902	0.995	
Cen-Pul | Po-Pr | vIps - vCon	-1.68	3.47	-8.48	5.11	-0.48	0.628	0.972	
Pul-Sha | Po-Pr | vIps - iIps	-2.08	6.52	-14.87	10.70	-0.32	0.750	0.972	
Cen-Sha | Po-Pr | vIps - iIps	1.38	6.57	-11.49	14.26	0.21	0.833	0.972	
Cen-Pul | Po-Pr | vIps - iIps	3.46	6.58	-9.44	16.37	0.53	0.599	0.972	

*Units in milliseconds (ms). Grey rows mark results presented in the main manuscript. *Abbreviations:* Est, estimate; CI, confidence interval; Sha, sham sonication; Pul, pulvinar sonication; Cen, central thalamic sonication; Pr, pre-sonication; Po, post-sonication; vCon, validly cued contralateral target; iCon, invalidly cued contralateral target; vIps, validly cued ipsilateral target; iIps, invalidly cued ipsilateral target; p_{adj}, p-value after correcting for multiple comparisons using the Benjamini-Hochberg method. † $p < 0.10$ before and/or after correction. * $p < 0.05$, ** $p < 0.01$, *** $p < 0.001$ after correction.
