# Supplementary material for: Targeting central thalamus with transcranial ultrasound reduces arousal in healthy volunteers
Source: Commun Biol. 2026 Oct 26;9:1403. doi: 10.1038/s42003-026-10637-7 (PMC13710743; doi:10.1038/s42003-026-10637-7)
Supplement: Supplementary file 3 [file 42003_2026_10637_MOESM3_ESM.pdf]

Description of Additional Supplementary files

File name: Supplementary Data 1

Description: Contains Figure 2 source data

File name: Supplementary Data 2

Description: Contains Figure 3 source data
