# Supplementary material for: Targeting central thalamus with transcranial ultrasound reduces arousal in healthy volunteers
Source: Commun Biol. 2026 Oct 26;9:1403. doi: 10.1038/s42003-026-10637-7 (PMC13710743; doi:10.1038/s42003-026-10637-7)
Supplement: Supplementary file 4 [file 42003_2026_10637_MOESM6_ESM.pdf]

nature portfolio

Corresponding author(s): Amber R. Hopkins

Last updated by author(s): Jun 20, 2026

Reporting Summary

Nature Portfolio wishes to improve the reproducibility of the work that we publish. This form provides structure for consistency and transparency in reporting. For further information on Nature Portfolio policies, see our Editorial Policies and the Editorial Policy Checklist.

Statistics

For all statistical analyses, confirm that the following items are present in the figure legend, table legend, main text, or Methods section.

n/a	Confirmed	
☐	☒	The exact sample size (n) for each experimental group/condition, given as a discrete number and unit of measurement
☐	☒	A statement on whether measurements were taken from distinct samples or whether the same sample was measured repeatedly
☐	☒	The statistical test(s) used AND whether they are one- or two-sided<br>*Only common tests should be described solely by name; describe more complex techniques in the Methods section.*
☐	☒	A description of all covariates tested
☐	☒	A description of any assumptions or corrections, such as tests of normality and adjustment for multiple comparisons
☐	☒	A full description of the statistical parameters including central tendency (e.g. means) or other basic estimates (e.g. regression coefficient) AND variation (e.g. standard deviation) or associated estimates of uncertainty (e.g. confidence intervals)
☐	☒	For null hypothesis testing, the test statistic (e.g. F, t, r) with confidence intervals, effect sizes, degrees of freedom and P value noted<br>*Give P values as exact values whenever suitable.*
☒	☐	For Bayesian analysis, information on the choice of priors and Markov chain Monte Carlo settings
☐	☒	For hierarchical and complex designs, identification of the appropriate level for tests and full reporting of outcomes
☐	☒	Estimates of effect sizes (e.g. Cohen's d, Pearson's r), indicating how they were calculated

Our web collection on statistics for biologists contains articles on many of the points above.

Software and code

Policy information about availability of computer code

Data collection	Stimuli and tasks were created and administered using PsychoPy (Version 2021.2.3112).
Data analysis	Mixed-effects modeling was performed using the lme4 (Version 1.1.36), lmerTest (Version 3.1.3), and emmeans (Version 1.11.0), and DHARMa (Version 0.4.7) packages in R (Version 4.4.3). Data cleaning and visualization were completed in Python (Version 3.11.11).

For manuscripts utilizing custom algorithms or software that are central to the research but not yet described in published literature, software must be made available to editors and reviewers. We strongly encourage code deposition in a community repository (e.g. GitHub). See the Nature Portfolio guidelines for submitting code & software for further information.

Data

Policy information about availability of data

All manuscripts must include a data availability statement. This statement should provide the following information, where applicable:

- Accession codes, unique identifiers, or web links for publicly available datasets
- A description of any restrictions on data availability
- For clinical datasets or third party data, please ensure that the statement adheres to our policy

The raw anonymized data from with work are available at the OSF repository associated with this publication: https://osf.io/pn8eq/overview?view_only=5f6b10f6638d4d8db56ece1b05c5a972.

Research involving human participants, their data, or biological material

Policy information about studies with human participants or human data. See also policy information about sex, gender (identity/presentation), and sexual orientation and race, ethnicity and racism.

Reporting on sex and gender	Individuals were asked to report their biological sex at intake. No gender information was collected. Sex and gender were not considered in the study design or recruitment procedures. Participants were recruited from a convenience sample through flyers posted around the UCLA campus. Twenty-seven participants completed the entire protocol and are included in the analyses, including two females and 25 males. Consent has been obtained for sharing individual-level data. Since there were so few biological females in our study, we lack the statistical power to perform post-hoc analyses to examine whether biological sex influences the effects of sonication with TUS in the different conditions and make any meaningful conclusions. To better adhere to the Nature journal guidelines for reporting on sex and gender in research, we explicitly state biological sex count in the abstract, re-ran all mixed-effects models in the analyses adding biological sex as a covariate, and explicitly discuss the lack of representation of biological sexes in the discussion section.
Reporting on race, ethnicity, or other socially relevant groupings	No race, ethnicity, or other socially relevant grouping information was acquired from the participants or considered in the experimental design.
Population characteristics	Right-handed and English-speaking adults (18 years of age or older) with normal or corrected-to-normal vision. Individuals were excluded from participation if they self-disclosed current or recent use of any psychoactive medications or recreational drugs (e.g., antidepressants or stimulants), a contraindication for entering the magnetic resonance imaging (MRI) environment (e.g., MR-incompatible implants), possible or planned pregnancy (in the short-term), or had hair longer than 0.25 inches over their left temporal region and were unwilling to shave it. The final sample included 27 individuals (age = 23.07 +/- 3.33, two females).
Recruitment	Participants were recruited using flyers posted around the University of California, Los Angeles (UCLA) campus. Participants were self-selected based on interest, availability, and eligibility. This introduced a bias towards young individuals (university setting) and individuals interested in neuroscientific research or brain stimulation. This limits the generalizability of our results to the broader populations, especially for older adults.
Ethics oversight	All recruitment and experimental procedures were approved by the Institutional Review Board at the University of California, Los Angeles (UCLA). Written informed consent was obtained from all participants prior to participation

Note that full information on the approval of the study protocol must also be provided in the manuscript.

Field-specific reporting

Please select the one below that is the best fit for your research. If you are not sure, read the appropriate sections before making your selection.

☐ Life sciences ☒ Behavioural & social sciences ☐ Ecological, evolutionary & environmental sciences

For a reference copy of the document with all sections, see nature.com/documents/nr-reporting-summary-flat.pdf

Behavioural & social sciences study design

All studies must disclose on these points even when the disclosure is negative.

Study description	Sham-controlled, single-blinded experiment with quantitative data.
Research sample	Right-handed and English-speaking adults (18 years of age or older) with normal or corrected-to-normal vision. Individuals were excluded from participation if they self-disclosed current or recent use of any psychoactive medications or recreational drugs (e.g., antidepressants or stimulants), a contraindication for entering the magnetic resonance imaging (MRI) environment (e.g., MR-incompatible implants), possible or planned pregnancy (in the short-term), or had hair longer than a ¼ inch over their left temporal region and were unwilling to shave it. The final sample included 27 individuals (age = 23.07 +/- 3.33, two females).
Sampling strategy	We used a convenience sampling procedure whereby any individuals who replied to the flyer and fit the eligibility criterion were enrolled in the experiment. No sample size calculation was performed. We aimed to acquire data from 30 participants because similar sample sizes are reported in previous work applying transcranial ultrasound stimulation in healthy volunteers (see e.g. Mueller et al., 2014; Legon et al., 2018; Atkinson-Clement et al., 2024).
Data collection	For every participant, we acquired an anatomical image (T1-weighted) image using a T1-weighted MPRAGE sequence using a 3T Siemens Prisma fit MRI scanner. Behavioral tasks were administered (and their data acquired) using PsychoPy (Version 2021.2.3) on a computer. The participant and one or two experimenters were present during each session. Participants were blinded to the experimental conditions and hypotheses but researchers were not.
Timing	July 2021 through February 2023. There were no gaps between collection periods.
Data exclusions	Individual sessions from participants were left out of the analyses on an analysis-by-analysis basis. Specifically, an individual session

Data exclusions	from a participant was excluded from the mixed-effects model if that participant was substantially influential in the model estimates (based on Cook's distance) and the participant's behavior in that session was erroneous when compared to the rest of the participants based on Tukey's criterion. This was done to ensure that no extreme values or changes in the outcome after sonication could drive the results. All data from all other sessions from these participants were included in the mixed-effects model to retain as much data as possible and improve estimates of the random effects. In total, for the Psychomotor Vigilance Task, one session from one participant was left out of the models for response time, response speed, and lapses in attention; and three sessions from three separate participants were left out of the model for the slowest 10% of responses (see Supplementary Fig. 1). For the Egly-Driver task, one session from one participant was left out of the model comparing correct responses between trial types and one session from one participant was left out of the model examining correct responses on main trials (see Supplementary Fig. 9).
Non-participation	Ten participants voluntarily withdrew from the study. Three dropped out after their MRI visit (did not complete any sonication sessions). Seven dropped out after at least one sonication session. No participants reported adverse experiences from sonication.
Randomization	The experiment took a 3 (ultrasound condition: sham, pulvinar, and central thalamic sonication) x 2 (task block: pre- and post-sonication) within-subjects design. Each participant contributed data to every combination of ultrasound condition and timepoint, making the experiment a fully crossed, repeated-measures (within-subjects) design. The order in which participants underwent the conditions and tasks was randomized.

Reporting for specific materials, systems and methods

We require information from authors about some types of materials, experimental systems and methods used in many studies. Here, indicate whether each material, system or method listed is relevant to your study. If you are not sure if a list item applies to your research, read the appropriate section before selecting a response.

Materials & experimental systems

n/a	Involved in the study	
☒	☐	Antibodies
☒	☐	Eukaryotic cell lines
☒	☐	Palaeontology and archaeology
☒	☐	Animals and other organisms
☒	☐	Clinical data
☒	☐	Dual use research of concern
☒	☐	Plants

Methods

n/a	Involved in the study	
☒	☐	ChIP-seq
☒	☐	Flow cytometry
☐	☒	MRI-based neuroimaging

Plants

Seed stocks	n/a
Novel plant genotypes	n/a
Authentication	n/a

Magnetic resonance imaging

Experimental design

Design type	n/a
Design specifications	n/a
Behavioral performance measures	n/a

Acquisition

Imaging type(s): Structural T1-weighted images (acquired for defining neuroanatomical regions to target with ultrasound and neuronavigation)

Field strength: 3T

Sequence & imaging parameters: TR = 2,400 ms, TI = 1,060 ms, TE = 2.12 ms, flip angle = 88, voxel size = 1×1×1 mm isotropic, matrix size = 256×256, 192 slices

Area of acquisition: Whole brain

Diffusion MRI: ☐ Used ☒ Not used

Preprocessing

Preprocessing software: The raw T1-weighted data was converted from DICOM to NIFTI format using dcm2niix.

Normalization: Linear and non-linear registrations between native structural (T1) and standard structural (MNI) images were performed using flirt and fnirt in FSL.

Normalization template: MNI

Noise and artifact removal: n/a

Volume censoring: n/a

Statistical modeling & inference

Model type and settings: n/a

Effect(s) tested: n/a

Specify type of analysis: ☐ Whole brain ☐ ROI-based ☒ Both

Anatomical location(s): Masks for each target region were generated in standard Montreal Neurological Institute (MNI) space by drawing 5-mm spheres over the predetermined target voxels (see Fig. 1e). Since we target the left central thalamus and the left pulvinar in the same individuals, we defined the target region for the central thalamus over more anterior aspects of the left central thalamus to maximize the distance between the target regions defined for the central thalamus and pulvinar and avoid co-modulation of our ultrasound targets.

Statistic type for inference (See Eklund et al. 2016): n/a

Correction: n/a

Models & analysis

n/a	Involved in the study	
☒	☐	Functional and/or effective connectivity
☒	☐	Graph analysis
☒	☐	Multivariate modeling or predictive analysis
